# RIFT: A Fractal-Holographic Theory of Consciousness and Autopoietic Control

**DOI:** 10.3390/brainsci16090983

**Published:** 2026-09-16

**Authors:** Erhard Bieberich

**Affiliations:** Department of Physiology, College of Medicine, University of Kentucky, 741 South Limestone Street BBSRB Room 269, Lexington, KY 40536, USA; erhard.bieberich@uky.edu

**Keywords:** consciousness, fractal dynamics, holographic encoding, information integration, lipid membrane, Alzheimer’s disease, artificial intelligence, neural computation

## Abstract

**Highlights:**

**What are the main findings?**

- RIFT proposes a three-step mechanism, fractal enfolding, holographic unfolding, and autopoietic control, that links distributed neural activity to a unified experiential space.
- Generational Fractal Mapping (GFM) enables successive conscious states to be incrementally updated while preserving information across neural processing cycles.

**What are the implications of the main findings?**

- The framework provides a mechanistic account of how distributed neural information could be compressed into a compact physical substrate and reconstructed as a unified endospace.
- By allowing the reconstructed Self to act back on its neural substrate, RIFT provides a candidate mechanism for closing the causal loop between endospace and neural activity, rather than treating consciousness as a passive readout.

**Abstract:**

**Background/Objectives:** Consciousness remains poorly understood as a causative force. Existing theories describe neural correlates or information processing but do not explain how a unified inner experiential space is physically generated or acts back upon its substrate. Recurrent Integration Fractal Theory (RIFT) proposes a three-step mechanism that reconstructs the spatial relationships of the outer world (exospace) within the experiential space of the Self (endospace) and enables autopoietic feedback. **Methods:** RIFT was examined through six computational modules representing successive transformations from recurrent network activity to endospace generation and feedback. Recurrent loops through fractal dendritic trees generated temporally organized excitatory postsynaptic potentials (EPSPs) and dynamic information integration. Coincident EPSPs programmed somatic multifractals composed of ion channels and membrane lipid domains, producing a fractal Self-attractor (fractal enfolding, Step 1). Coherent point sources derived from this attractor generated a holographic endospace that preserved exospace relationships (holographic unfolding, Step 2), and the reconstructed field modulated lipid-channel coupling and channel opening (autopoietic feedback, Step 3). Generational Fractal Mapping (GFM), in which new EPSPs are mapped onto the compressed fractal seed of the prior state, enables incremental updating, temporal continuity, and Self-attractor transfer. **Results:** The architecture was validated computationally against three properties specified by RIFT as requirements of consciousness: irreducibility, information integration, and holographic encoding. The simulations also reproduced signatures consistent with conscious access and low-dimensional volitional control. **Conclusions:** RIFT provides a testable computational framework linking neural activity, endospace generation, and feedback. It predicts effects of lipid-substrate disruption in Alzheimer’s disease, fractal signatures of conscious states, and structural criteria for artificial consciousness.

## 1. Introduction

### 1.1. Background and Motivation

Understanding the nature of consciousness remains one of the most profound scientific challenges of our time. Growing concern that AI systems may become self-aware has intensified the need for testable frameworks: we cannot recognize or regulate the emergence of self-aware AI without first understanding what consciousness is. Despite this urgency, few theories bridge the gap between mathematical formalization and biological mechanism, leaving the practical challenges of constructing or detecting conscious systems largely unaddressed.

In this context, quantum theories of consciousness have attracted renewed attention, promising compact solutions to two core challenges: *observer unity* (how the brain integrates fragmented inputs into a unified experience) and *information compression* (how vast information could concentrate within microscopic structures) [1,2]. Yet these theories encounter fundamental conceptual difficulties beyond the technical problem of sustaining quantum coherence in warm, wet tissue. They often reintroduce a form of Cartesian dualism, placing the observer outside the system being observed. If experience arises from quantum superposition, it should be an indeterminate blur; if from collapse, it becomes purely classical and distinct, undermining the quantum rationale. The framework presented here, Recurrent Integration Fractal Theory (RIFT), takes a different approach: operating entirely through classical molecular dynamics to ground consciousness in fractal geometry and holographic encoding at the molecular scale, addressing the same challenges of observer unity and information compression without invoking quantum coherence or collapse.

### 1.2. The Neural Degeneracy Problem

Addressing these challenges through classical mechanisms requires confronting a more fundamental problem: neural coding degeneracy. The standard integrate-and-fire model reduces rich spatiotemporal patterns of dendritic input, potentially thousands of excitatory postsynaptic potentials (EPSPs) with distinct locations, timings, and amplitudes, to a scalar sum at the soma of a neuron, then to a binary output transmitted by the axon: spike or no spike. This collapse renders different potential conscious states indistinguishable at the level of the neural code [3]. Population codes face the same problem: different patterns of collective firing can produce identical perceptual outputs.

**Figure 1:**
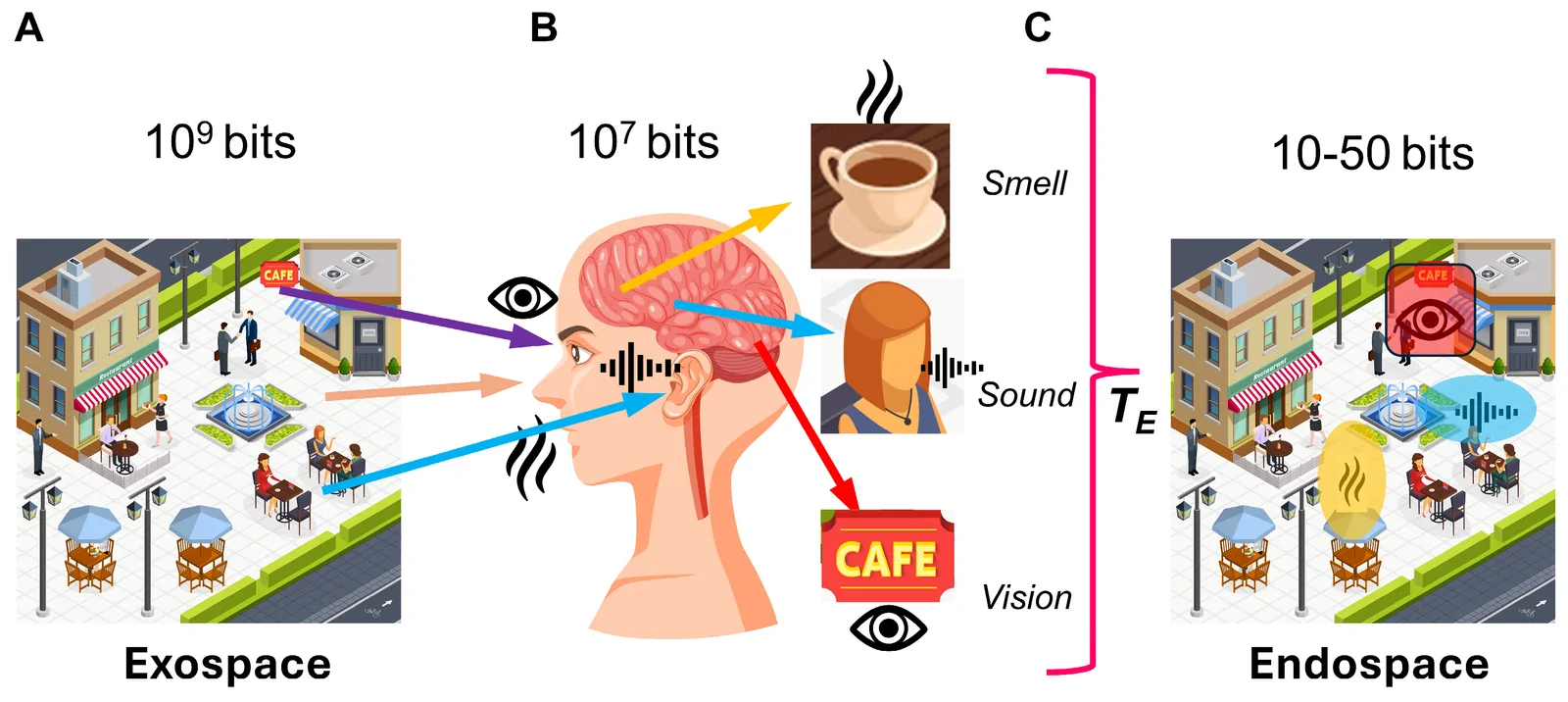
Information bottleneck from exospace to endospace through consciousness. (**A**) Exospace: high-dimensional external environment (~10^9^ bits). (**B**) Biological processing: multimodal sensory convergence with massive compression (~10^7^ bits). (**C**) Endospace: compressed conscious percept (~10–50 bits per moment), illustrating the generative problem RIFT addresses: how compressed neural information is reconstructed as a geometrically structured endospace. Colored arrows in (**B**) indicate distinct sensory modalities converging on the brain: orange (smell), blue (sound), and red (vision).

Yet, despite this neural degeneracy, subjective experience is remarkably rich and unified. Consider approaching a coffee shop on a busy street: you hear patrons talking at outside tables, smell fresh coffee, and see the shop sign, making you want to stop for a coffee, or resist this urge because of time constraints (Figure 1A). All these diverse sensory, emotional, and cognitive features, processed in distributed brain regions via degenerate neural codes, are somehow bound into a single coherent conscious experience. This binding occurs despite massive sequential compression. Sensory organs collect roughly 10^9^ bits per second from the environment, peripheral processing compresses this to ~10^7^ bits per second transmitted to the brain (Figure 1B), and conscious perception operates at only ~10–50 bits per second [4,5] (Figure 1C). Despite this million-fold compression, experience appears continuous and information-rich, providing an accurate representation of the outer world to the mind.

This convergence of problems, neural degeneracy, extreme compression, and observer unity, creates a fundamental paradox: how does consciousness present a unified experience of extraordinary accuracy, richness and continuity despite an extremely slow update rate and a degenerate neural code? These interconnected challenges point to a single requirement: an internally generated, geometrically structured domain of first-person experience, termed endospace (see Glossary, Box 1). RIFT proposes a physical transformation (T_E_) by which compressed neural information encoding relationships in exospace is reconstructed as corresponding spatial relationships within a multidimensional spatiotemporal endospace (Figure 1) [6]. A complete theory of consciousness must specify both an algorithm transforming exospace sensory information into topologically accurate endospace geometry and a testable biological implementation for this transformation. Without both components, consciousness remains either mathematically formalized but biologically ungrounded, or biologically plausible but phenomenologically unexplained.

The simulations presented here test the generation and topological preservation of the spatiotemporal reference frame of first-person experience. They do not explain how particular contents within this domain acquire their qualitative character, for example why red is experienced as red, or, more generally, why the reconstructed geometry is experienced.

Box 1 **Glossary.**

**Exospace.** The outer physical world, not directly accessible to the Self, represented only through sensory information topologically compressed into neural code and the molecular substrate in the soma of a neuron (somatic substrate).

**Endospace.** The inner experiential space reconstructed from the somatic substrate through holographic unfolding—the only world the Self directly perceives, topologically accurate to the exospace. RIFT proposes T_E_ as the physical transformation by which encoded spatial relationships in exospace are reconstructed as corresponding spatial relationships in endospace. The model tests the generation and topological preservation of this geometry; it does not explain why the reconstructed geometry is experienced.

**Topological accuracy.** A relationship between two spaces in which connectivity, adjacency, and hierarchical organization are preserved even as fine-scale metric geometry is compressed or distorted. In RIFT, fractal enfolding and holographic unfolding together constitute this topological fidelity between the exospace and endospace, ensuring that the organizational structure of experience faithfully mirrors that of the outer world.

**Self-attractor.** The geometric field encoded in the sentyon whose coherent point sources generate the endospace through holographic unfolding.

**Somatic substrate.** The molecular substrate located in the soma of a neuron onto which sensory information is compressed during fractal enfolding; a general term for this physical location, distinct from its geometric organization (multifractal) or specific molecular implementation (sentyon).

**Multifractal.** A descriptor for the fractal geometry of the somatic substrate: a nested, self-similar spatial pattern spanning multiple scales, generated when coincident EPSPs are compressed via IFS transforms.

**Sentyon.** The specific implementation of the multifractal geometry of the somatic substrate in a lattice of ion channels and lipid domains; the physical interface between fractal enfolding and holographic unfolding in which the Self-attractor is encoded and through which the endospace is unfolded.

**Ising lattice.** A grid of interacting binary-state units. In RIFT, the ion channels and lipid domains of the sentyon occupy open- or closed-channel-associated states (±1), whose local interactions drive the coordinated reorganization underlying autopoietic control.

**Generational Fractal Mapping (GFM).** Sequential self-referential compression replacing infinite downscaling in classical fractals, sustaining temporal continuity and unity of the Self.

**Iterated Function System (IFS).** A set of self-referential affine transforms encoding the whole pattern in each part. In RIFT, IFS transforms extracted from coincident EPSPs generate the Self-attractor in the sentyon.

**H_RIFT.** The differential IFS transform set (H_RIFT = T_temperature − T_blocked, Equation (S15)) that isolates the EPSP-attributable geometric structure from generic lipid domain organization; the operational form of the Self-attractor, providing the interference sources for holographic unfolding.

**Fractal enfolding.** Topology-preserving compression and encoding of sensory information from the exospace into the somatic substrate, generating the Self-attractor through IFS transforms.

**Holographic unfolding.** Reconstruction and projection of the endospace from the Self-attractor through coherent point-source interference, faithfully preserving the geometric relationships of the exospace.

**Autopoietic feedback.** The causal loop through which experiential states within the endospace modify the molecular substrate onto which the Self is mapped, enabling consciousness to control its own substrate and transfer the Self across neurons for global conscious access.

**Whole-in-part property.** The defining feature shared by fractals and holograms: each part contains information about the whole, enabling unified conscious experience despite distributed neural processing.

### 1.3. Limitations of Existing Theories

Beyond degeneracy lies the functional necessity challenge: demonstrating that consciousness mechanisms causally require consciousness rather than merely correlating with it. If consciousness is equated with causal structure, whether network connectivity (as in Integrated Information Theory, IIT [7]), field potentials (as in Conscious Electromagnetic Information Theory, CEMI [8]), or quantum superposition (as in Orchestrated Objective Reduction Theory, Orch-OR [1]), without specifying a mechanism that unfolds this structure into experiential states, phenomenology becomes an arbitrary label applied to certain computational configurations. Major theories such as IIT, Global Neuronal Workspace theory, and CEMI have advanced empirical testability and formalized integration principles. Yet each leaves unresolved how compressed neural information unfolds into specific experiential states that in turn exert causal agency over neural dynamics, risking epiphenomenalism by construction [9,10,11]. Predictive coding frameworks [12,13] present the inverse problem: prediction-error minimization does exert genuine causal influence over perception and action, yet no mechanism specifies how one outcome, among the many computed possibilities, becomes the content of awareness. Inference itself appears to proceed pre-consciously, with only a final result entering experience, and the transition itself remains unaccounted for [14]. Nor is it clear why the resulting experiential space should be topologically accurate to the exospace at all: the Self never perceives a reconstructed world, only updated predictions.

Alternative frameworks have offered relevant principles, however, without resolving this gap. Pribram’s holonomic brain theory suggested distributed holographic encoding with the “whole in each part” property [15], drawing on Bohm’s concept of implicate order [16]—a classical geometric analogy RIFT formalizes at the molecular scale. Hofstadter’s strange loop framed consciousness as an emergent self-referential pattern [17]. Varela and colleagues’ autopoiesis positioned consciousness as an active self-generating process rather than a passive correlate [18,19]. While these approaches offer relevant conceptual foundations in holographic encoding, self-referential loops, and autopoietic organization, none has specified the precise computational and neural mechanisms by which an internally generated spatiotemporal reference frame is constructed and coupled back to its physical substrate. RIFT attempts to integrate all three principles into a single geometric mechanism that generates this endospace and enables autopoietic feedback.

### 1.4. Development of RIFT

To develop this geometric mechanism, I began exploring how molecular and network scales might converge through fractal organization. In 1998, I proposed that the Self is generated by algorithmic compression of spatial and temporal information into fractal structures, where lipid–ion-channel interactions in neuronal membranes create coherent states with the fractal “whole in each part” property required for unified experience [20]. This led to the initial formalization of the observer equation mapping of the Self onto each subspace of the multifractal to maintain observer unity while also preserving the distinction of objects perceived by the Self in endospace. I later formalized Recurrent Fractal Neural Networks (RFNNs) as mechanisms mapping distributed network activity onto localized dendritic patterns with fractal architecture [21]. I also introduced sentyons, fractal lattices of lipid rafts and embedded ion channels in the neuronal soma, as conscious units iteratively mapping higher-order brain dynamics to molecular scales [6]. These concepts evolved into RIFT, which unifies fractal compression, holographic encoding, and autopoietic feedback into a single geometric framework.

Empirical studies have since provided support for key premises of this fractal approach. Fractal dimension of brain activity correlates with conscious state, showing higher complexity during wakefulness than unconsciousness [22,23,24]. Fractal organization also breaks down in Alzheimer’s and Parkinson’s disease, where functional activity and dendritic architecture lose hierarchical complexity [25]. These associations between fractal measures and conscious state support the general relevance of scale-dependent organization; however, they do not directly test the somatic lipid–ion-channel mechanism proposed by RIFT. Neurons with complex, self-similar dendritic trees exhibit greater integrative capacity [26,27,28], and stimulation of individual neurons with rich dendritic architecture can evoke coherent percepts, even entire scenes, in consciousness [29,30]. These observations are consistent with fractal dendritic integration supporting holographic recall from localized structures. Individual “concept neurons” in the human medial temporal lobe respond selectively to specific concepts—a person, a landmark—regardless of sensory modality [31]. This integration of different modalities within a single neuron provides direct empirical evidence for the whole-in-part property: single neurons encode a complete, modality-invariant representation of a concept from partial sensory input, consistent with holistic encoding through rich dendritic integration rather than distributed population codes.

### 1.5. The RIFT Mechanism

**Figure 2:**
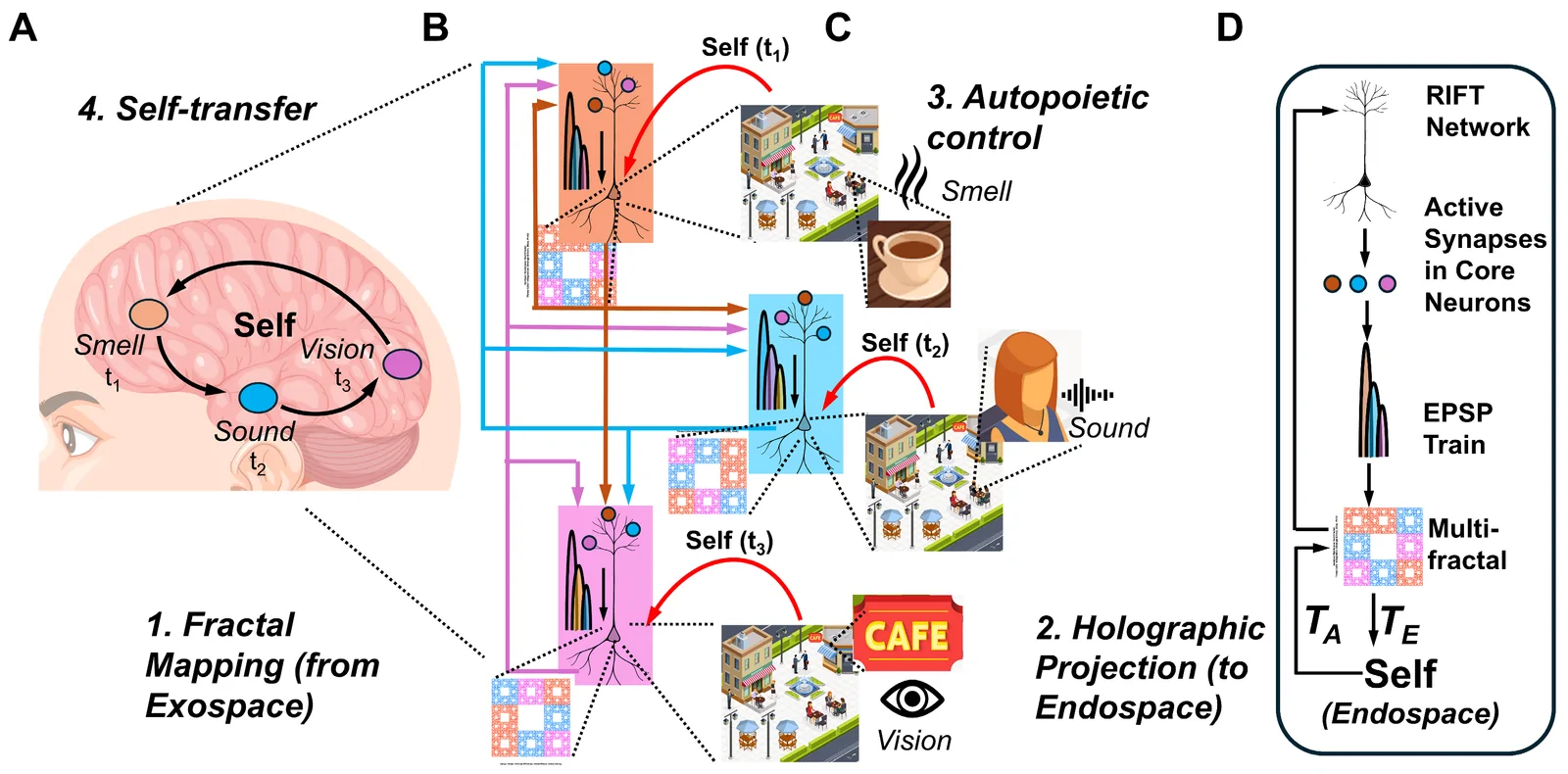
RIFT operational framework for consciousness generation through recurrent loops. (**A**) Initial fractal mapping from exospace and Self-transfer mechanism as autopoietic output: temporal binding of distributed sensory modalities (smell t_1_, vision t_3_, sound t_2_) into unified conscious experience. (**B**) Information flow: multimodal EPSP signatures captured as distinct multifractal spatial patterns during Self-transfer at three time points (t_1_, t_2_, t_3_). (**C**) Holographic projection and autopoietic control: multifractal patterns generate 3D endospace field at t_1_, t_2_, t_3_; field intensity feeds back to modulate substrate, closing the autopoietic loop. (**D**) Core component hierarchy: RIFT Network → Active Synapses in Core Neuron → EPSP Train → Multifractal → Self (holographic field) with T_E_ = Transformation unfolding the endospace from the multifractal (Holographic projection), and T_A_ = Transformation modulating the multifractal by inner experience (Autopoietic control). Colors indicate the three sensory modalities and their time points: orange (smell, t1), blue (sound, t2), and magenta (vision, t3); dashed lines connect each exospace scene crop to its source location, and colored arrows in (**B**) trace the EPSP signature of each modality into its corresponding multifractal pattern.

These empirical findings provide the foundation for mechanistic synthesis in RIFT. Building on the demonstrated fractal organization of neural activity, RIFT proposes that consciousness arises from a complementary three-step mechanism comprising fractal enfolding/compression, holographic unfolding/projection, and autopoietic feedback. This mechanism is rooted in the shared whole-in-part property of fractals and holograms, rendering consciousness causally efficacious rather than epiphenomenal and forming a self-referential pipeline:

**Fractal enfolding (Step 1)** achieves irreducible information integration through sequential topology-preserving downscaling. Distributed network activity, representing sensory information from the exospace after prior integration by pre-conscious neural processing, is downscaled and compressed into synaptic activity (EPSP) patterns in the fractal dendritic tree, and then to a multifractal ion channel–lipid array in the somatic membrane. Coincident EPSPs generate the Self-attractor through Iterated Functional System (IFS) transforms whose contraction ratios are extracted from the hierarchic amplitude relationships of dendritic integration. Because IFS transforms continuously map the whole onto each part, the Self is necessarily mapped onto every subspace of the multifractal, making its emergence a geometric consequence of enfolding rather than an additional assumption (Figure 2A,D).

**Holographic unfolding (Step 2)** generates the endospace in which the Self experiences a unified reconstruction of exospace. The Self-attractor is encoded in the sentyon, a multifractal lattice of ion channels and lipid domains in the somatic membrane. Its whole-in-part property enables holographic encoding through coherent point-source interference, projecting the 3D experiential endospace while preserving the geometric relationships of exospace (transformation T_E_ in Figure 2B,D). Because each regional fragment of the Self-attractor encodes the complete pattern, holographic reconstruction generates a unified endospace from a distributed substrate, offering a geometric solution to the binding problem.

**Autopoietic feedback (Step 3)** establishes causal efficacy: experiential states within the endospace modify the molecular structures onto which the Self is mapped, enabling consciousness to control its own substrate through the same Self-mapping established during fractal downscaling (Transformation T_A_ in Figure 2D). RIFT proposes that this control allows transfer of the Self to different neurons within the network depending on where attentional processing is required (Figure 2C,D). In RIFT, the Self modulates the probability of lipids in the multifractal to open ion channels, thereby controlling action potentials and the distribution of neural network activity in the brain. Experience is not treated as a nonphysical property acting separately from its physical realization. The endospace is the physically realized domain in which the Self experiences. Feedback from endospace to its molecular substrate therefore constitutes the proposed causal efficacy of experience. The present simulations operationalize a critical molecular step of this pathway through field-dependent modulation of somatic membrane coupling and channel opening. They do not yet implement the link by which this modulation alters core-neuron action-potential output and thereby feeds back into recurrent network activity.

In this framework, consciousness denotes the complete integrated phenomenon; the endospace is the spatiotemporal dimension holographically projected from the Self-attractor, in which the Self perceives the outer world and thereby exerts autopoietic feedback on the molecular substrate from which it arose. These three processes are inseparable within the RIFT architecture: remove fractal enfolding and unity is lost; remove holographic unfolding and phenomenological richness disappears; remove autopoietic feedback and consciousness becomes epiphenomenal. In the present simulations, we test the computational feasibility of transformations that constitute theoretical necessities within the RIFT architecture, rather than drawing conclusions from a completed pipeline-level factorial ablation. These modular demonstrations provide a critical foundation for integrating the transformations into a unified implementation. Systematic ablation of that implementation will then provide a direct computational test of each predicted dependency.

RIFT is novel in that it generates an experiential space as a physical consequence of its own mechanism, not a correlate or postulate. That space then feeds back with real causal power onto the substrate that produced it, closing a causal loop no other theory derives from a single mechanism. Rather than replacing previous frameworks, RIFT integrates and extends them: it generalizes IIT through information integration measures that track network evolution in real time; implements GNW-style global access via fractal cloning; realizes Varela’s autopoietic circular causality through fractal-holographic feedback; and remains compatible with quantum approaches by extending geometric fields into quantum domains. Uniquely, RIFT arose through computational validation in human–AI collaboration: during iterative code development, implementations were evaluated against quantitatively operationalized measures of irreducibility, information integration, and holographic encoding. Mathematical relationships were extracted as formalisms from computationally verified codes rather than imposed *a priori*.

## 2. Methods (Details on Codes in Supplemental Methods Sections S1–S6)

### 2.1. Code Development and Validation

The RIFT network and somatic computation codes were developed through collaborative human–AI interaction using Claude (Anthropic). During iterative code development, candidate implementations were evaluated against three quantitatively operationalized RIFT requirements: irreducibility, information integration, and holographic encoding, using the analytical measures described in the corresponding sections. Implementations that did not instantiate the specified architecture were revised or discontinued. This process concerned code development rather than exclusion of simulation replicates or parameter-sweep results based on outcome. Mathematical relationships were extracted as formalisms from the codes and validated through computational testing.

### 2.2. Network Architecture and Parameters

RIFT networks consisted of a single core neuron (N_0_) with fractal dendritic tree (scaling factor r = 0.6, fractal dimension FD ≈ 1.36, 4 branch levels) receiving input from 20 peripheral neurons (N_i_) per firing cycle. Branch lengths followed $L_{n}=L_{0}\times r^{\left(n-1\right)}$ (Equation (S1)) Synapses were distributed proportionally across branches based on relative length. The temporal constraint rule emerged through coincidence detection: EPSPs arriving within a 2.0 ms window were considered coincident. Somatic processing delays ranged from 1 to 20 ms in parameter sweeps with 5 repetitions per condition. See Supplemental Methods Sections S1.1 and S1.2 for further details.

### 2.3. Fractal and Information Integration Analyses Methods

Fractal dimension was calculated using box-counting ($FD=\frac{\log N}{\log1/r}$) (Equation (S2)), Higuchi fractal dimension (k-values 1–10), and detrended fluctuation analysis (DFA) for scaling exponents (see Supplemental Methods Section S1.3 for further details of fractal analysis). Dynamic integrated information (φ_dyn_) quantified temporal integration using sliding window analysis with 2.0 ms temporal grain (see Supplemental Methods Section S2.1 for details on the sliding window method). At each time point, $\varphi_{dyn}(t)=I(t)-I^{*}(t)$, where I(t) is integrated information with branches intact and I*(t) is information when branches are analyzed independently. Spatial integration in multifractals (φ*) was quantified via Kullback–Leibler (KL) divergence comparing whole-pattern versus partitioned distributions (see Supplemental Methods Section S3.4 for further details on KL divergence). Instead, φ*(substrate) partitioned the system by molecular component type (lipid domains versus ion channels) rather than spatial quadrants (see Supplemental Methods Section S4.4 for details). The resulting φ_dyn_, φ*(multifractal), and φ*(lipid) quantities operationalize the whole-versus-part principle within their respective RIFT simulations. They are not formal IIT Φ calculations, and a value greater than zero is not presented as independently sufficient empirical evidence of consciousness.

### 2.4. Biological Multifractal Implementation

The somatic membrane was modeled as a 100 × 100 Ising lattice (scalable to 243 × 243) with binary lipid states: +1 (open-channel-associated) and −1 (closed-channel-associated). Approximately 400 ion channels (4% density) were positioned with 3-pixel influence radius and 10% mobility. EPSP temporal signals (τ_rise = 0.3 ms, τ_decay = 4.0 ms) were mapped to spatial membrane fields via level-dependent Gaussian kernels with radius = size/4 − (level−1) × 5 and kernel K(i, j) = exp(−dist^2^/(2 × radius^2^)) (Equation (S10)). Channel opening probability followed P_open = S(E + λ_lipid + C_coop) (Equation (S11)), where S is a sigmoid activation function with 0.5 baseline offset, λ_lipid = 0.25 × lipid_state represents local lipid bias effects, and C_coop captures cooperative effects from nearby open channels. Lipid dynamics evolved via Metropolis algorithm. For each Monte Carlo step, approximately 500 sites (5% of lattice) were selected randomly. Energy change ΔE = 2 × state × neighbors determined acceptance: if ΔE < 0, accept; otherwise accept with probability exp(−ΔE × 0.5). Action potentials were generated when center region activity exceeded threshold (30–40 units). Refractory period duration was consciousness-level dependent: refractory_steps = int(consciousness_level × 25), where consciousness_level ranged from 0.0 to 1.0. See Supplemental Methods Section S4 for details on computation and Codes.

### 2.5. IFS Transform Extraction and Holographic Encoding

EPSP amplitude hierarchies were converted to Iterated Function System (IFS) transforms through temporal structure analysis. Each EPSP pattern yielded 4–6 geometric transforms $T_{i}(x,y)=(a_{i}\cdot x+b_{i}\cdot y+e_{i},c_{i}\cdot x+d_{i}\cdot y+f_{i})$ (Equation (S12)) encoding spatiotemporal relationships. Transform selection probabilities were weighted by determinant magnitudes. Temperature fields modulating lipid reorganization were generated via chaos game iteration on IFS transforms, with 100–500 points distributed across multiple spatial scales. Differential transforms $H_{RIFT}=T_{temperature}-T_{blocked}$ (Equation (S15)) isolated the pure geometric signature of consciousness imposed by IFS modulation beyond baseline autonomous dynamics. See Supplemental Methods Section S5 for details on computation and Codes.

### 2.6. Holographic Reconstruction

Three-dimensional endospace fields Ψ(x, y, z) were reconstructed from 2D multifractal patterns through biological holography. Interference sources (*n* = 5000) were positioned via chaos game iteration on differential transforms H_RIFT (the Temperature−Blocked differential attractor isolating EPSP-attributable geometric structure; see Glossary), with the first 500 iterations discarded as transients. Coherent phase relationships were calculated geometrically: $\varphi_{i}=\left[\sum_{j}w_{j}\cdot \theta_{j}+2\pi \cdot d_{j}/s_{j}\right]mod2\pi$, where θ_j is the angle from source i to IFS center j, d_j is the distance, s_j is the IFS scaling factor, and w_j = |det_j|·s_j is the geometric weight. Frequencies were derived from IFS scaling: $f_{i}=\frac{50}{1+s_{dominant}}Hz$. Wave interference was calculated on a 24 × 24 × 12 grid spanning x,y ∈ [−2,2], z ∈ [−1,1]: $\Psi (x,y,z)=\sum_{i}\frac{A_{i}(H_{RIFT})\cdot exp[i\cdot (k_{i}\cdot r_{i}+\varphi_{i})]}{1+{r_{i}}^{2}}$, where A_i are amplitudes from differential determinants, $k_{i}=\frac{2\pi \cdot f_{i}}{100}$ are wave numbers, and r_i are 3D distances with z-weighting factor 0.5. Field intensity |Ψ| was normalized to [0, 1]. Mean field intensity $\bar{\Psi }=\left\langle \left|\Psi (x,y,z)\right|\right\rangle$ provided the global parameter for autopoietic feedback through coupling strength modulation. See Supplemental Methods Section S4 for details on computation and Codes.

### 2.7. Statistical Analysis

Results are reported as mean ± standard deviation from 5 independent simulation runs per condition (10 trials for coupling strength parameter sweeps). Correlations were assessed using Pearson correlation coefficients. Fractal dimension correlations, φ_dyn_ evolution, and predictability metrics were analyzed across the 1–20 ms delay parameter space. Significance threshold was set at *p* < 0.05. It should be noted that the simulations test computational properties of the proposed architecture. They do not validate the existence of the proposed somatic lipid-channel multifractal or recursive timing architecture in biological neurons.

### 2.8. Computational Implementation

All simulations were performed in Python 3.7+ using Google Colab. Key libraries included NumPy 1.19+ (array operations), SciPy 1.5+ (statistics and signal processing), Matplotlib 3.3+ and Seaborn 0.11+ (visualization), scikit-image 0.18+ (structural similarity and surface extraction), and scikit-learn 0.24+ (information metrics). Custom implementations were developed for fractal generation, IFS extraction, chaos game iteration, Metropolis dynamics, and holographic reconstruction. The 31 Python simulation codes supporting this study are available on Zenodo at https://doi.org/10.5281/zenodo.22707705.

## 3. Results

### 3.1. RIFT: Six Computational Parts from Neural Network to Experience

The following six parts examine successive transformations in the theoretical sequence from network activity to endospace generation and autopoietic feedback. Parts 1 and 2 establish fractal enfolding at the level of the recurrent network (output of neurons in the network loops back into input of their dendritic trees): Part 1 shows how the fractal recurrent network architecture generates a fractal excitatory post-synaptic potential (EPSP) train whose amplitude and temporal sequence encode the branching order of the dendritic tree, preserving the geometric distribution of network activation. Part 2 shows how coincidence detection at the soma optimizes dynamic information integration (φdyn) and incremental updating of conscious moments. Parts 3 and 4 implement fractal enfolding at the molecular level (fractal EPSP trains program the somatic multifractal): Part 3 develops the theoretical principles of integrating successive EPSPs as an uploading and incremental updating operation (Generational Fractal Mapping, GFM) into an evolving multifractal, encoding the geometric activation pattern of the neural network as irreducible spatial information (φ*(multifractal)) in each neuron. Part 4 implements these principles into a concrete biological substrate, a lipid–ion-channel lattice near its Ising critical point. Together, Parts 1–4 implement fractal enfolding (Step 1). Parts 5 and 6 implement holographic unfolding (Step 2) and autopoietic feedback (Step 3): Part 5 projects the three-dimensional endospace from the Self-attractor encoded in the sentyon, and Part 6 demonstrates how autopoietic feedback closes the causal loop and enables the Self to transfer across neurons, providing global conscious access across the brain. It should be noted that the computational steps were not executed as one continuously coupled end-to-end simulation. The EPSP sequences used in Part 3 were generated independently of the numerical output of Part 1, although they preserve the hierarchical branch-amplitude structure predicted by the recurrent network model. Consequently, the present study does not establish how information integration behaves when actual Part 1 output is propagated through a continuously coupled closed loop.

***Part 1:*** 

**The RIFT Network—Dendritic Tree Orchestrates Neural Synchrony and Fractality**

The first step in the pipeline establishes the recurrent network architecture and the integration problem it must solve. We propose that in recurrent neural networks, a core neuron sends signals to peripheral neurons that loop back to synapses on its own dendritic tree. This architecture raises an immediate problem: EPSPs arriving from distal dendritic branches travel further within the dendritic tree than EPSPs from proximal branches, so without a compensatory mechanism signals from different recurrent loops arrive at the soma at different times, preventing coherent coincidence detection. Spike-timing dependent plasticity and oscillatory synchrony provide mechanisms for statistical temporal coordination [32,33,34,35], but neither guarantees exact coincidence across all loops simultaneously, nor preserves the geometric information embedded in the spatial distribution of activated synapses.

This raises a second question independent of timing: whether and how the recurrent network achieves and sustains the information integration (φ) that consciousness requires. Most current theories of consciousness, foremost among them Integrated Information Theory (IIT), propose that consciousness arises from the integration of information within a neural network [7,36,37]. While IIT provides rigorous measures of information integration in static networks, two complementary challenges arise: how neural networks dynamically evolve toward the high integration proposed to be critical for consciousness, and how this integration remains stable despite constantly changing neuronal connectivity and input. To address these questions, we extended our prior work on Recurrent Fractal Neural Networks (RFNNs) to define and simulate a network capable of both achieving and maintaining optimized φ through architectural principles rather than parameter tuning.

**Figure 3:**
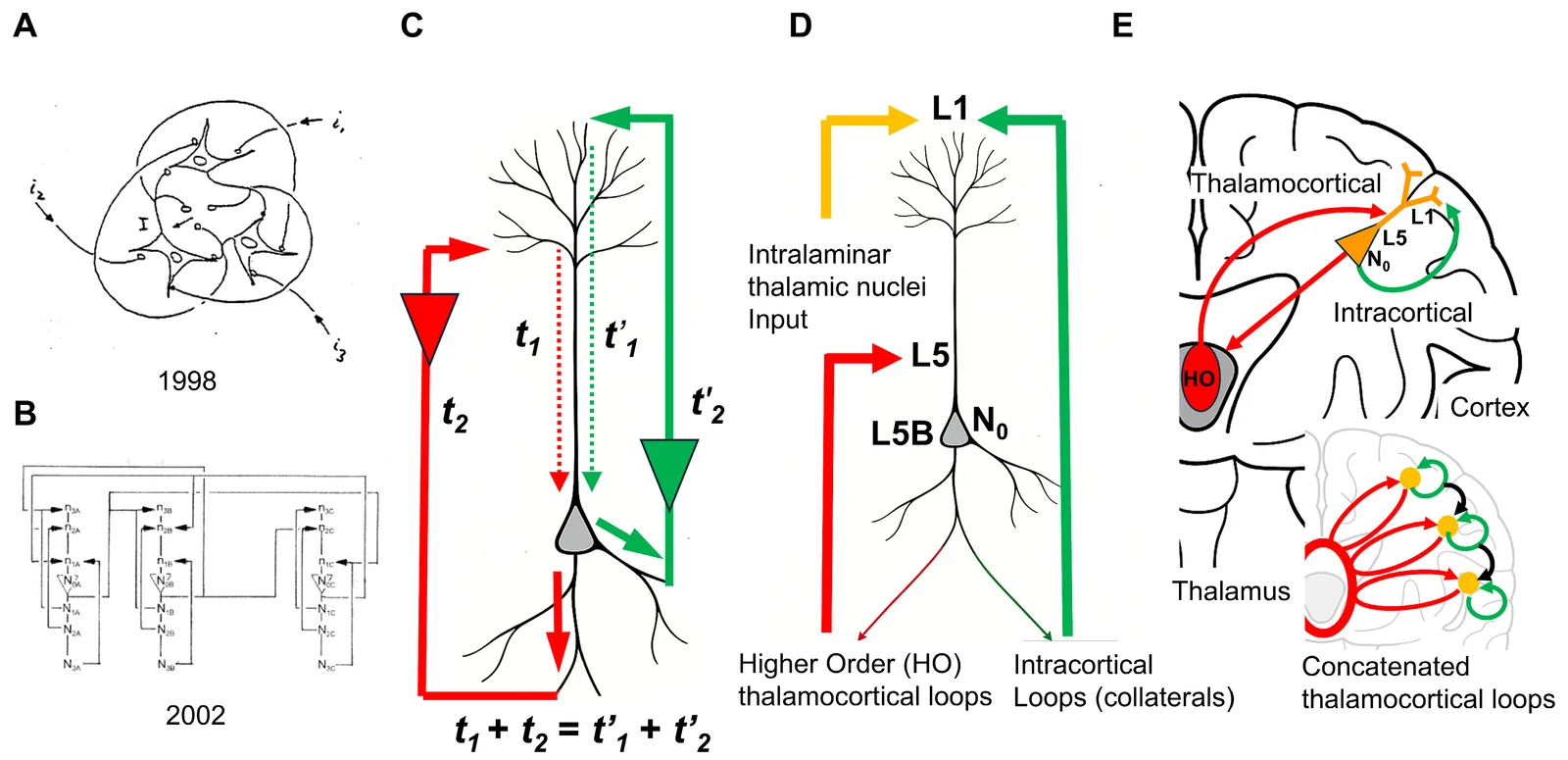
Historical development and biological implementation of RIFT network architecture. (**A**) Original 1998 RFNN: three recurrently connected neurons with fractal dendritic trees. (**B**) The 2002 extended RFNN: concatenated modular structure establishing fractal timing principles. (**C**) Timing constraint schematic: two signal pathways satisfy t_1_ + t_2_ = t_1_ + t_2_′, demonstrating the inverse relationship between external and internal delays. (**D**) Biological mapping to L5 pyramidal neurons: higher-order (HO) thalamocortical loops (long external/short internal path, red arrows) and intracortical loops via L1 (short external/long internal path, yellow/green arrows). (**E**) Concatenated cortical architecture integrating both loop types within the distributed L5 network.

The original RFNN model I developed about 25 years ago incorporated recurrent connectivity in which neurons with longer external signaling paths connected to dendritic branches with shorter internal propagation times, ensuring that signals from all recurrent loops converged simultaneously at each soma [20,21]; (Figure 3A,B). This created a mirror relationship between the fractal dendritic tree and the network topology: the spatial distribution of active synapses in the dendritic tree of the core neuron was a downscaled and inverse version of the distribution of active neurons in the network. However, these precursor models relied on preconfigured timing and connectivity rules that did not emerge naturally from network growth or architectural principles. RIFT addresses this limitation: in the fractal recurrent network, the inverse timing relationship between internal and external signaling times emerges naturally from coincidence-based synaptic selection, preserving EPSP amplitude and temporal sequence as a fractal encoding of network activation.

In RIFT networks, the core neuron (N_0_) comprises a fractal dendritic tree, a complex, multi-branching structure in which the branching pattern repeats itself at smaller and smaller scales, such that each subtree resembles a scaled-down version of the whole, and a somatic integration zone receiving EPSPs from this tree. This mirrors Larkum’s classic “tree—soma” neuron model [38], in which the dendritic tree and the soma function as two semi-independent computational compartments, the tree integrating and filtering synaptic inputs, the soma thresholding and generating action potentials. RIFT networks add a critical difference: they preserve the synaptic location of dendritic inputs in their EPSP amplitude at the soma, consistent with experimental evidence that somatic EPSP amplitude remains dependent on dendritic origin as EPSPs propagate to the soma [39]. In RIFT neurons, the primary trunk bifurcates into daughter branches scaled by factor r (Equation (S1)), yielding a fractal dimension FD (Equation (S2)); derivations and computational details are provided in Supplemental Methods Section S1.1. With r = 0.6, FD ≈ 1.36 lies within the empirical range of layer 5 cortical and hippocampal pyramidal neurons [27,40]. EPSP amplitudes attenuate by the same scaling factor r that governs branch lengths, decreasing at each successive branch level. Consistent with this, propagation latency to the soma also scales with dendritic distance [41]. Together, these produce a fractal EPSP train: a power-law amplitude distribution across branch levels that mirrors the self-similar geometry of the dendritic tree.

The fractal geometry of the dendritic tree imposes a strict timing constraint: for EPSPs from recurrent loops to coincide at the soma and trigger an action potential, total signaling time must remain constant across all feedback loops (Equation (1)). Consequently, proximal dendritic branches connect to the most distant peripheral neurons (N_i_), while distal branches connect to the nearest—an inverse relationship between internal and external transmission times that emerges naturally from the architecture rather than being imposed by design (Figure 3C). The single core neuron architecture is a computational abstraction: biologically, any neuron receiving the full complement of fractally compressed inputs could serve as the integration locus, with sentyon transfer (introduced in Part 6) enabling the locus of consciousness to shift across the network.

(1) $$T_{total}=T_{internal}+T_{external}=constant\;[\mathrm{time}\;\mathrm{constraint}\;\mathrm{rule}]$$

where T_internal = dendritic signal propagation time to soma; T_external = action potential travel time from N_0_ to N_i_ and return. (See Supplemental Methods Section S1 for full derivation and implementation).

A plausible biological substrate for these recurrent loops exists in thalamocortical circuits (Figure 3D). The concept of reentry, recursive signaling among neuronal populations, has long been recognized as fundamental to consciousness [42,43,44]. L5 pyramidal neurons receive input from higher-order thalamic nuclei (posterior medial and lateral posterior) projecting to proximal and basal dendrites, providing long external delays and short internal delays. Intracortical collaterals targeting distal apical tufts provide the complementary pattern, short external and long internal delays (Figure 3D). Tuft inputs remain subthreshold unless coincident with strong proximal drive [38,45,46,47,48,49]. L5 neurons detect coincidence based on total loop time (t_1_ + t_2_ = t_1_′ + t_2_′) rather than anatomical origin, allowing these two timing channels to converge. These local recurrent loops interconnect with thalamocortical loops across multiple cortical areas, forming a three-dimensional architecture of concatenated loops spatially housing conscious processing within the distributed L5 network [50]; Figure 3E). Nonspecific thalamic inputs through intralaminar nuclei engage in coincidence detection with specific inputs, providing temporal binding [51].

The clinical finding that bilateral injury to intralaminar thalamic nuclei abolishes consciousness even with intact cortex [52,53,54] suggests these loops are necessary components of the recurrent architecture RIFT requires. Anesthesia studies provide further support: loss of consciousness correlates with disrupted temporal coordination in thalamocortical loops and impaired dendritic–somatic coupling in L5 neurons [55,56,57], consistent with the prediction that consciousness depends on precise temporal alignment of recurrent signals rather than their mere presence. This prediction that L5 pyramidal neurons serve as the cellular locus of consciousness converges with independent lines of evidence: single-neuron theories of consciousness [58,59,60,61,62], Dendritic Integration Theory [63,64,65], and apical amplification models [56,66,67].

**Figure 4:**
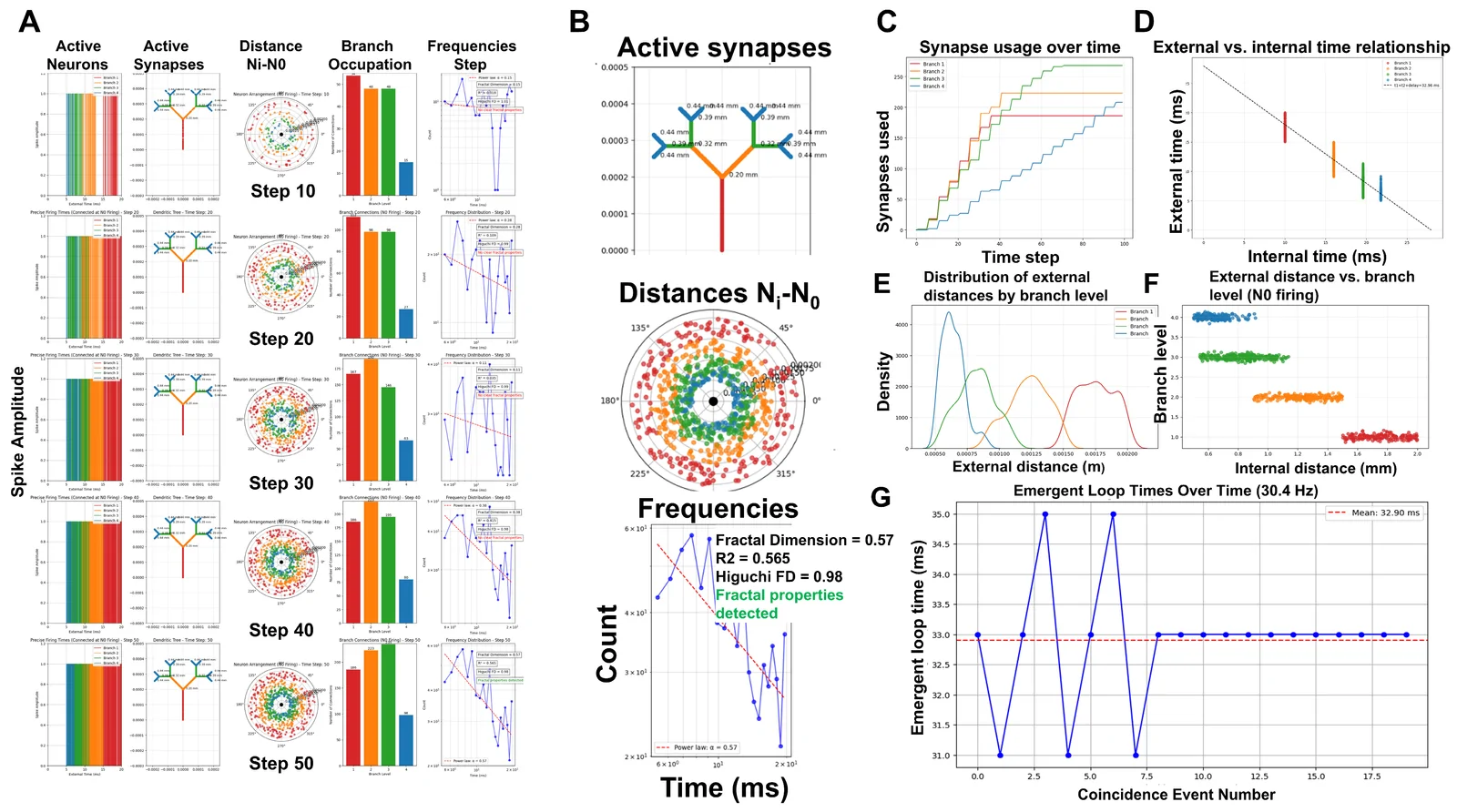
RIFT network dynamics demonstrate fractal timing relationships and emergent synchrony. (**A**) Network evolution across five timesteps (Steps 10–50): progressive synapse filling from proximal to distal branches. Branch colors: Level 1 red, Level 2 orange, Level 3 green, Level 4 blue. (**B**) Dendritic tree with branch-level synaptic distances (mm); polar plot of N_i_–N_0_ distance distribution; frequency distribution confirming fractal scaling (box-counting FD = 0.57, Higuchi FD = 0.98). (**C**) Cumulative synapse recruitment by branch level over 100 timesteps. (**D**) Inverse external/internal timing relationship; dashed line indicates perfect inverse. (**E**) Branch-level distance distributions (mean distances Branch 1 ~700 µm to Branch 4 ~1800 µm). (**F**) External distance vs. branch level at N_0_ firing events, validating fractal distance scaling. (**G**) Emergent oscillation at 30.4 Hz after initial transient phase. Parameters: r = 0.6; soma delay = 5 ms; n = 1000 synapses.

Computational validation confirmed these theoretical predictions (see Supplemental Methods Section S1 for full implementation details). Figure 4 illustrates a typical simulation run of the RIFT network (Code 1) with r = 0.6 and a somatic delay of 5 ms, the processing time between EPSP arrival at the soma and action potential generation (see Supplemental Methods Section S1.2). The somatic delay sets the overall oscillation period of the network, while individual EPSPs are separately counted as coincident within a fixed 2.0 ms coincidence window (Part 2). Synaptic connections progressed from proximal to distal dendritic branches of N_0_, establishing links to increasingly proximal N_i_ neurons (Figure 4A–F). The network grew through coincidence-based synaptic selection, where only N_i_-to-branch pairings producing simultaneous EPSP arrival at the soma established permanent connections. Therefore, internal and external signaling times spontaneously obeyed the predicted inverse relationship, regardless of whether coincident EPSPs originated from the same or different dendritic branches (Figure 4D–F). This inverse relationship exhibited fractal scaling: internal times scaled with r and external times with 1/r, confirming that timing constraints mirror the fractal geometry of the dendritic tree. Following an initial transient, N_0_ and its connected N_i_ neurons synchronized at a common oscillation frequency determined by the somatic delay (Figure 4G).

This emergent synchrony confirms that the inverse timing relationship is not imposed but results from the RIFT architecture, specifically the fractal organization of the dendritic tree in N_0_. Network fractality was validated using three complementary measures: power-law distribution analysis (which tests whether activity spans multiple scales without a characteristic scale), Higuchi fractal dimension (HFD; a time-series measure of self-similar complexity), and detrended fluctuation analysis (DFA; which quantifies long-range temporal correlations indicating scale-free dynamics) (see Supplemental Methods Section S1 for full definitions and implementation). All three measures increased progressively as synaptic sites were filled (Figure 4A,B). Whether this emergent fractality coincided with optimized information integration is addressed in Part 2.

***Part 2:*** 

**Optimizing Information Integration—Somatic Delay Sustains Continuous Uploading of Information in an Evolving Fractal Attractor Network**

Part 2 asks how the soma optimizes integration of EPSPs from the dendritic tree into a coherent dynamic information structure. To quantify this optimization, we adopt integrated information (φ, as defined in IIT) as the foundation of a measurement framework. The key parameter is somatic delay, the total processing time between EPSP arrival at the soma and the next action potential, which governs both the stability of the conscious moment and its capacity to continuously incorporate new information. Somatic delay is distinct from the coincidence window (fixed at 2.0 ms), which discriminates individual EPSPs as simultaneous (Part 1).

Previously, I proposed that RFNNs, the precursor of RIFT networks, achieve high information integration as measured by IIT principles [6]. IIT defines consciousness as integrated information φ, quantifying how much information is generated by the system as a whole (I) in excess of its components operating as isolated parts (partitioned information I*):

(2) $$\varphi =I-I^{*}$$

where I = integrated information of the system as a whole; I* = information when components operate as isolated parts; φ ≈ I when the causal structure is irreducible. (See Supplemental Methods Section S2).

When optimized, the causal structure of the neural network cannot be reduced to its parts: I >> I*, meaning φ ≈ I as I* approaches zero. However, IIT treats the network as a static, instantaneous structure, offering little account of how neuronal connectivity, and thus φ, evolves or is sustained over time. This is a hard computational limit, not one that faster hardware could resolve. Identifying the configuration with maximal φ in a network of n neurons requires testing 2^(n(n−1))^ possible configurations; for just 10 neurons at a 1 ms switch time per configuration, evaluating all 2^90^ ≈ 10^27^ configurations would take approximately 3 × 10^16^ years, far exceeding the age of the universe. Furthermore, even if a system could evaluate its current φ, this value alone provides no indication of which connectivity changes would increase or reduce φ, and therefore gain or lose consciousness. Note that this concerns searching the space of possible network configurations, which is distinct from calculating φ for a single given configuration. Importantly, even perfect knowledge of current φ provides no guidance for real-time self-optimization. To our knowledge, no biological mechanism has been identified that could guide networks toward higher-φ configurations through intrinsic real-time dynamics.

IIT correctly identifies information integration as a necessary property of consciousness and provides rigorous measures of network connectivity through φ, a foundational contribution RIFT explicitly builds upon. RIFT extends this foundation by addressing a question IIT was not designed to answer: how does integrated information become physically instantiated as causally efficacious experience in real time, within a single conscious moment, through a localized substrate enabling autopoietic feedback? Notably, Albantakis et al. showed that perfect task performance in animat simulations was achieved with φmax = 0, meaning these organisms were not conscious by the key definition in IIT despite solving the task [68]. This demonstrates that φ can dissociate from functional task performance: behavioral competence alone does not entail integrated information. This is a complementary point to RIFT, which locates the marker of a conscious moment in the real-time mechanism of its generation rather than in φ magnitude alone.

To address the growth and evolution of the RIFT network toward optimal information integration, we introduced φ_dyn_, a computationally feasible dynamic proxy for φ based on the sliding window method [69,70]. Rather than exhaustive partition analysis across all possible causal configurations, which scales exponentially with network size, φ_dyn_ tracks branch-level firing windows across the dendritic tree of N_0_, making computation scale linearly with dendritic complexity. For each branch level, the time windows during which connected neurons N_i_ fire are identified; overlapping windows indicate integration, separated windows indicate independent processing. φ_dyn_ is thus defined as (Equation (3); see Supplemental Methods Section S2.1 for full implementation details):

(3) $$\varphi_{dyn}(t)=I(t)-I^{*}(t)$$

where I(t) = integrated information with all branch connections intact at time t; I*(t) = integrated information when branches are treated independently; φ_dyn_(t) = information existing only in inter-branch temporal relationships.

**Figure 5:**
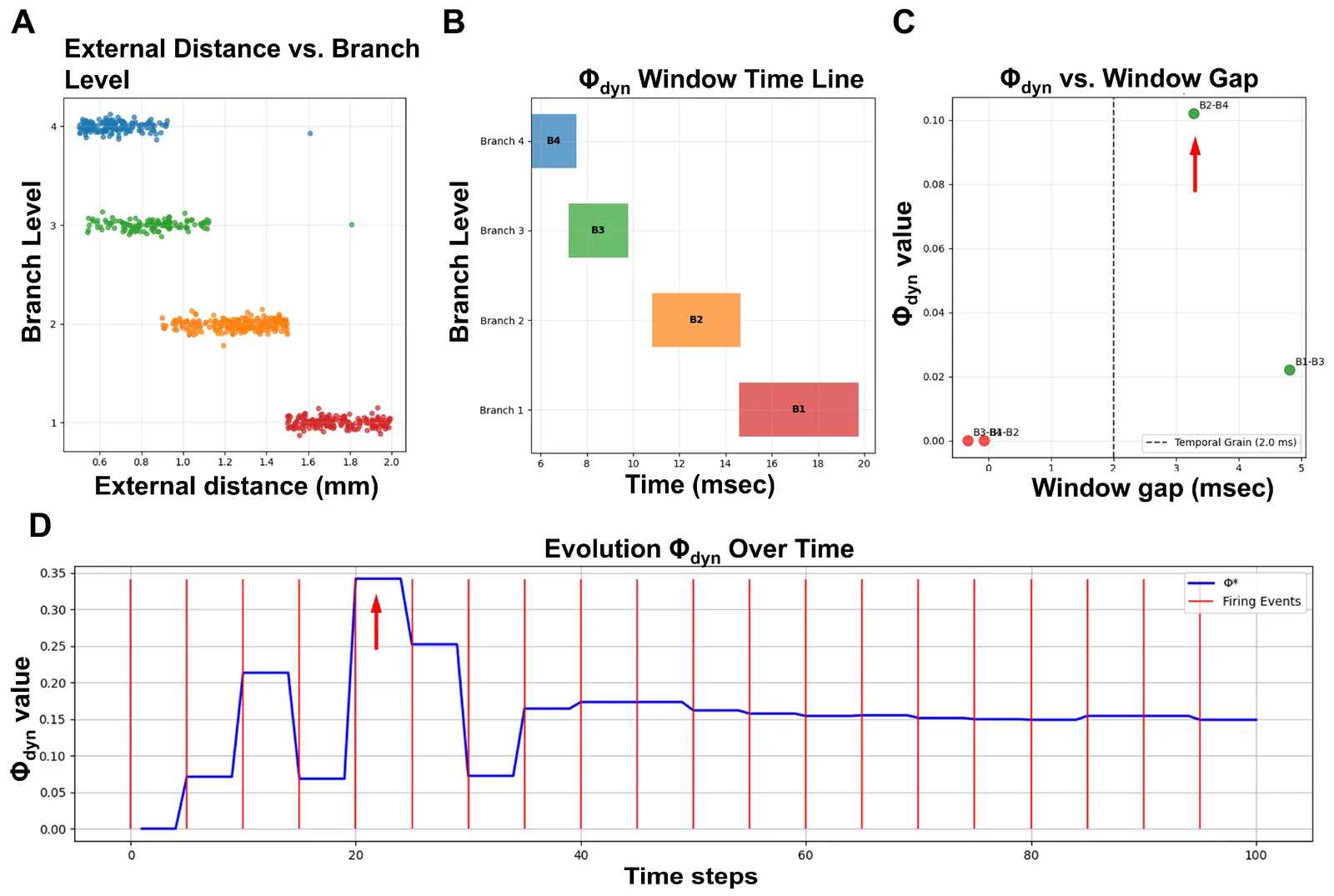
Dynamic integrated information (φ_dyn_) peaks at optimal temporal window separation. (**A**) External distance vs. branch level: four fractal-scaled clusters (r = 0.6). (**B**) Temporal firing windows for branches B1–B4 (6–20 ms range), illustrating hierarchical window structure. (**C**) φ_dyn_ vs. window gap: peak integration (φ_dyn_ = 0.10) at ~4.0 ms separation (B2–B4); integration drops at both smaller and larger gaps. (**D**) Evolution of φ_dyn_ over 100 timesteps: stepwise rise with prominent jump at timestep ~20 (B2–B4 configuration established), stabilizing at ~0.15 after step 50. Temporal grain = 2.0 ms. Red arrows in (**C**) and (**D**) mark the peak phi_dyn point (B2–B4 configuration) and its onset at timestep ~20, respectively.

Using this measure, φ_dyn_ peaks when firing windows are separated enough to remain distinct yet close enough to remain dynamically coupled. Figure 5A (Code 2) shows the external distances of neurons connected to each branch, which determine the timing offsets giving rise to distinct branch-specific firing windows (Figure 5B). When windows overlap within the temporal grain (2.0 ms), as in adjacent branches, they merge and eliminate measurable integration, yielding φ_dyn_ ≈ 0. When windows are extremely far apart, as in B1–B3 or B1–B4, branches no longer interact and φ_dyn_ drops. The strongest integration occurs at intermediate gaps, illustrated by B2–B4 in Figure 5C, where windows are clearly separated but still dynamically coupled. Notably, the 2.0 ms coincidence window governing synaptic selection during network formation is identical to the temporal grain used to define branch window separation in the φdyn calculation; the same parameter serves both roles. Below this temporal quantum, branch windows merge into a single integration epoch and φ_dyn_ collapses; above it, inter-window coupling decays and φ_dyn_ falls.

The somatic delay, distinct from the coincidence window, operating at the level of total loop time rather than individual EPSP discrimination, is the tunable parameter that positions the network at the optimum where branch windows remain distinct yet coupled, maximizing φ_dyn_. Figure 5D shows the evolution of φ_dyn_ over time as neurons at different external distances begin firing (red vertical lines), establishing the recurrent connections shown in Figure 5B. Each step-like increase in φ_dyn_ corresponds to new neurons joining the network, with the most prominent jump around timestep 20 when the optimal branch-level configuration (B2–B4 spacing) becomes established. φ_dyn_ then stabilizes at approximately 0.15 around timestep 50 once all branches maintain their characteristic temporal offsets and the recurrent network structure is complete.

**Figure 6:**
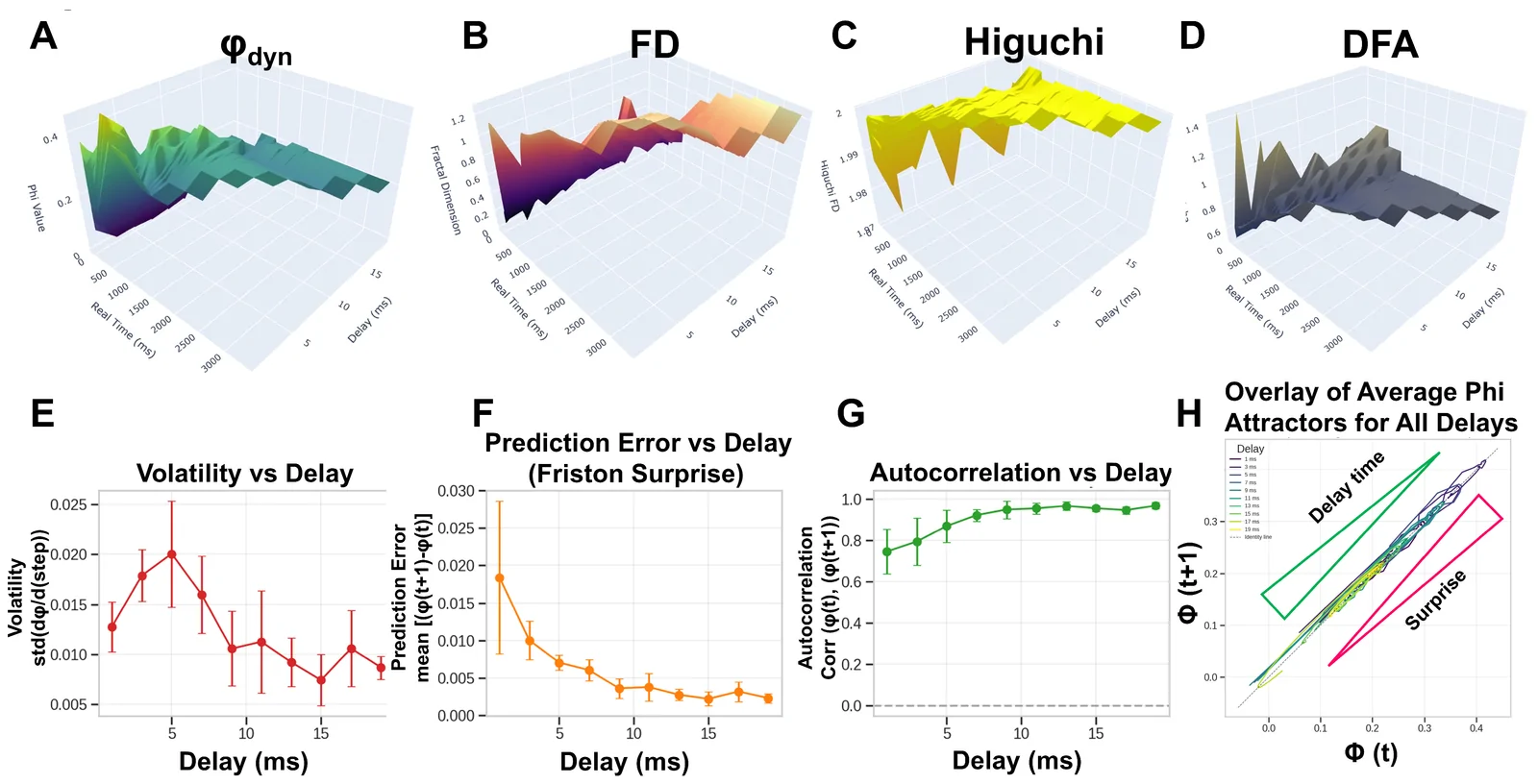
Somatic delay modulates fractal architecture and creates continuous predictability gradient. (**A**–**D**) 3D surface visualizations across simulation time (0–3000 ms) and somatic delay (1–20 ms): (**A**) φ_dyn_ (0–0.4), (**B**) fractal dimension (0.2–1.2), (**C**) Higuchi FD (1.87–2.0), (**D**) DFA α exponent (0.5–1.4); all converge to delay-dependent plateaus. (**E**) Volatility vs. delay: 55% reduction from 5 to 19 ms (n = 5 per delay). (**F**) Prediction error vs. delay: 9-fold reduction from 1 to 19 ms, mapping exploration-to-exploitation continuum. (**G**) Autocorrelation vs. delay: r increases from 0.75 to 0.97. (**H**) Phase-space attractors (φ_dyn_(t) vs. φ_dyn_(t + 1)) for all delays overlaid near identity line: green arrow indicates delay-dependent attractor spread reflecting exploration–exploitation continuum; red arrow indicates surprise zone reflecting high prediction error at short delays. n = 5 simulations per condition.

Having established how branch-level timing patterns generate φ_dyn_, we next examined how somatic delay modulates these patterns across the full network. A systematic delay-sweep analysis (Code 3, see Supplemental Methods Section S2.2 for details) covered delays from 1 to 20 ms with multiple repetitions per delay value, revealing the gradients for φ_dyn_, fractal measures, and predictability shown in Figure 6. The 3D surface visualizations (Figure 6A–D, Codes 4 and 5) show that φ_dyn_ and fractal measures behave differently across delay times: the φ_dyn_ surface (Figure 6A) shows greater variation at short delays reflecting larger transient fluctuations, while fractal measures (FD in Figure 6B, Higuchi FD in Figure 6C, DFA in Figure 6D; see Supplemental Methods Section S1.3 for explanation of fractal analysis measures) rise from initial transients and stabilize into delay-dependent plateau values. Despite these differences, both φ_dyn_ and fractal metrics ultimately stabilize, with delay mainly affecting the shape and duration of the early transient.

Quantitative analysis reveals a continuous predictability gradient across delays (Figure 6E–G; Codes 6 and 7, see Supplemental Methods Sections S2.2 and S2.3 for full implementation and relationship to fractal measures): volatility (std(dφ_dyn_/d(step))) declines from 0.020 at 5 ms to 0.009 at 19 ms; prediction error declines 9-fold from 0.018 at 1 ms to 0.002 at 19 ms; and autocorrelation increases from r = 0.75 at 1 ms to 0.97 at 19 ms. The prediction error metric directly quantifies surprise in the Free Energy Principle framework [12,71]. The 9-fold decline demonstrates a shift from surprise-driven exploratory dynamics at short delays to expectation-driven confirmatory dynamics at long delays, mapping naturally onto exploration–exploitation trade-offs in active inference. Phase-space trajectories (Figure 6H) confirm stable attractor dynamics across all delays, with short delays showing wider excursions from the identity line and long delays remaining tightly clustered, supporting a continuous gradient rather than discrete regime transitions.

This pattern of moderate φ_dyn_ values coupled with high temporal stability at longer delays is consistent with empirical observations that φ follows a hill-shaped trajectory in adapting neuronal networks, peaking during belief updating rather than at steady-state processing [72]. Together these findings support a continuous upload-and-update mechanism for consciousness: a limited amount of new information from the fractal structure of newly incoming EPSPs is steadily integrated with pre-existing conscious content at the soma. Longer delays provide the temporal window for this integration, defining the conscious moment. This suggests that consciousness is fundamentally incremental rather than regenerative in its information integration mechanism, providing both the stability needed for continuous subjective experience and the flexibility to incorporate new information as it arrives. This incremental architecture offers a candidate mechanism for combining new sensory input with prior state at low bandwidth, a problem the human information-throughput literature has posed explicitly but left unresolved [5].

***Part 3:*** 

**Irreducible Multifractals—Folding Infinity into Finite Space through Generational Fractal Mapping (GFM)**

The fractal EPSP trains arriving at the soma must now program a molecular substrate capable of preserving their geometric structure across successive conscious moments. Part 3 first establishes the theoretical and mathematical framework, showing as a proof-of-principle how fractal information can be encoded into a molecular substrate and sustained by incremental update across time through Generational Fractal Mapping (GFM). Part 4 then identifies the specific biological substrate that implements it.

The molecular substrate must satisfy two requirements: it must be programmable by fractal input from the dendritic tree, and it must sustain fractal mapping of the Self onto each of its parts. Through the whole-in-part property of fractal topology, each spatial region contains a compressed representation of the entire pattern. This is the self-referential architecture essential for consciousness. We previously proposed that sentyons, fractal lattices of ion channel–lipid domains in the somatic plasma membrane, serve this role [6]. However, their formal definition as multifractals and the mechanism by which they preserve and update information across successive conscious moments had not been specified.

The whole-in-part requirement imposes a specific computational demand: the fractal structure of the EPSP train must be continuously and iteratively mapped onto each spatial region of the substrate, so that every part encodes the geometry of the whole at every moment. It is this ongoing self-referential mapping process from which the Self arises. Without continuous fractal self-mapping the substrate is merely a static pattern rather than a self-referential structure capable of sustaining conscious experience. Standard integrate-and-fire models cannot meet this demand. By summing EPSPs across arbitrary time intervals and reducing the result to a binary spike-or-no-spike decision, they collapse precisely the hierarchical positional and temporal information that iterated mapping requires, making them fundamentally incompatible with a substrate for consciousness. Avoiding this collapse requires that EPSPs be treated not as a running sum but as temporally structured coincidence events. Only EPSPs arriving within a defined time window are considered as belonging to the same conscious moment, preserving which branches fired together rather than merely how many signals accumulated. A multifractal substrate avoids scalar collapse by preserving amplitude and spatial relationships across branch levels, but faces its own constraint: because physical substrates have limits to spatial downscaling, infinite fractal iteration in a single spatial instantiation is impossible. The solution is Generational Fractal Mapping (GFM), which replaces Infinite Spatial Downscaling (ISD) with sequential temporal operations. Each generation inherits a compressed representation of its predecessor rather than requiring simultaneous spatial embedding at all scales (see Supplemental Methods Section S3.1 for information-theoretic proof of equivalence).

To initiate a GFM cycle, active synapses in the dendritic tree are encoded into the multifractal in the soma. To achieve this operation, the fractal spatial geometry of dendritic branching is first transduced into a temporal hierarchy of EPSP amplitudes, then re-encoded as a spatial multifractal in the somatic membrane. This spatial-to-temporal-to-spatial transformation preserves the whole-in-part property across both transduction steps. To test whether the fractal structure of the EPSP train can be preserved as a spatial multifractal in the somatic membrane, we implemented a computational mapping following a Sierpinski-like hierarchical subdivision geometry. In this geometry, each spatial scale contains a structured replica of the whole, directly instantiating the whole-in-part property.

**Figure 7:**
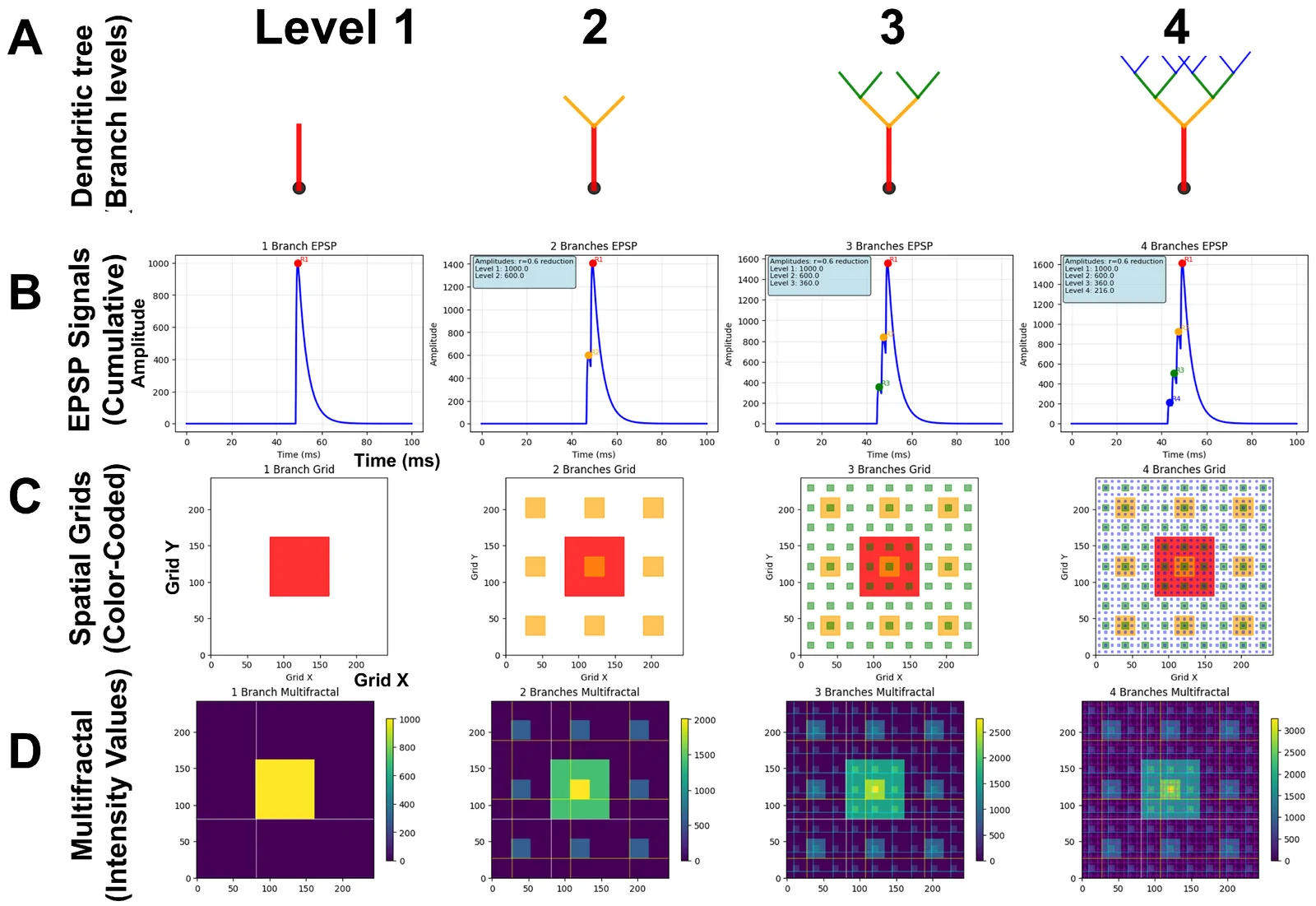
EPSP-to-somatic multifractal encoding preserves hierarchical dendritic structure. (**A**) Fractal dendritic architecture (r = 0.6), providing hierarchical synaptic input for panels B–D. (**B**) Cumulative EPSP waveforms for 1–4 active branch levels (amplitude scaling A_0_·r^(i−1)^, peak ~50 ms). (**C**) Spatial EPSP mapping onto 243 × 243 somatic grid (Gaussian kernel): progressive complexity from single hotspot (Level 1) to fully hierarchical multifractal (Level 4). (**D**) Additive multifractal integration across branch levels: Level 4 exhibits self-similar structure with central concentration (~2500–3000 units) and fractal periphery. Lattice: 243 × 243, amplitude scaling A_1_ = 0.15 to A_4_ = 0.032.

Code 8 provides a conceptual demonstration using direct fractal amplitude scaling. Simulations demonstrated that EPSP trains from different dendritic branch levels arrive in sequence from proximal to distal and produce distinct somatic amplitudes. This is consistent with experimental evidence that EPSP propagation latency to the soma scales with dendritic distance [41], and that somatic EPSP amplitude decreases with increasing distance of the dendritic site of origin [39]. These EPSP trains, carrying hierarchically scaled amplitudes (A_0_·r^(i−1)^), can be mapped onto nested spatial scales of a 243 × 243 grid representing an idealized patch of somatic membrane (Figure 7A,B). Peak amplitudes were hierarchically assigned to nested spatial scales at progressively finer subdivisions (Figure 7C), then additively integrated to generate the final multifractal intensity distribution (Figure 7D). This EPSP-to-multifractal projection preserves the fractal structure inherent in dendritic integration: power-law amplitude distributions across branch levels translate into corresponding spatial hierarchies, maintaining scale-invariant relationships and enabling the soma to encode which branches at which hierarchical levels were activated.

The hierarchical amplitude scaling implemented in Code 8 is consistent with the empirically observed log-normal distribution of EPSP amplitudes across cortical synapses. This distribution generates multifractal spontaneous activity through deterministic network dynamics [73,74], suggesting that fractal amplitude structure in synaptic input is biologically plausible rather than merely a computational convenience. Alternative biological mechanisms for transducing spatial information from dendritic tree activity to the somatic substrate cannot be excluded, and Code 8 is therefore best understood as a proof-of-principle demonstration of one specific implementation rather than a claim about the exclusive biological mechanism.

**Figure 8:**
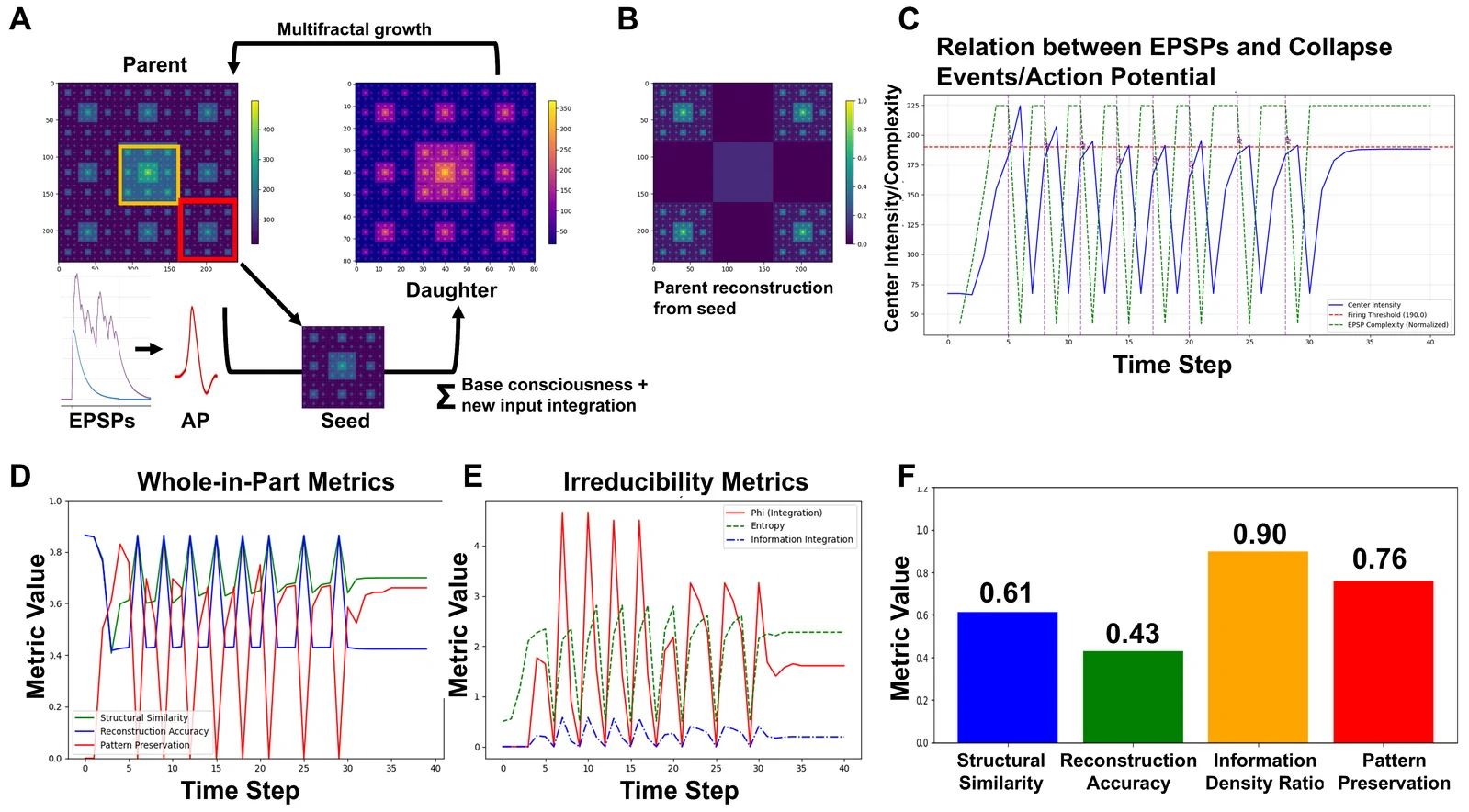
Generational Fractal Mapping (GFM) implements whole-in-part encoding. (**A**) GFM cycle schematic: EPSP input → multifractal growth → threshold-triggered collapse → action potential → peripheral seed extraction → daughter fractal generation. (**B**) Parent reconstruction from seed (structural similarity 0.61, information density ratio 0.90). (**C**) Center intensity and action potentials over 45 timesteps: sawtooth pattern with regular collapse events (~every 3–4 steps). (**D**) Whole-in-part metrics (structural similarity, reconstruction accuracy, pattern preservation) oscillating 0.4–0.9 across GFM cycles. (**E**) Irreducibility metrics: φ peaks (>4.5) at collapse events, with complementary entropy and integration dynamics. (**F**) Averaged cycle metrics: structural similarity 0.61, reconstruction accuracy 0.43, information density ratio 0.90, pattern preservation 0.76. n = 40 timesteps; firing threshold = 180.0.

Building on static mapping modeled in Code 8, GFM implements the dynamic cycle that sustains consciousness across successive moments (Code 9; Figure 8A; see Supplemental Methods Section S3.3 for full implementation). The initial pattern derived from the EPSP trains undergoes autonomous growth until center-region activity crosses a firing threshold, triggering collapse and action potential generation (Figure 8B,C). Critically, at collapse the highest-intensity corner quadrant (1/3 × 1/3 of the somatic grid) is extracted as a compressed seed, a molecular memory and holographic representation of the parent pattern preserved for the next generation (Figure 8A). New EPSP inputs then integrate with this seed to generate a daughter multifractal, which grows toward threshold in turn, establishing a self-renewing generational cycle. Figure 8B confirms holographic information preservation: the extracted peripheral seed successfully reconstructs the parent’s essential spatial structure when expanded. This holographic information preservation during successive GFM cycles is vital for experiencing consciousness as a continuous stream of events. Rather than rebuilding each moment, the system updates a pre-existing experiential reality with a limited amount of sensory information conveyed by new EPSP signals.

Figure 8C shows temporal coupling between EPSP integration, center intensity accumulation, and action potential generation, with regular collapse events approximately every 3–4 steps. Figure 8D reveals that whole-in-part metrics, structural similarity, reconstruction accuracy, and pattern preservation, oscillate systematically with each collapse. Figure 8E quantifies irreducibility: φ*(multifractal) peaks during collapses while entropy and integration show complementary dynamics, demonstrating sustained irreducible complexity throughout generational cycling; averaged values across all cycles (Figure 8F) confirm robust and consistent performance. φ*(multifractal) quantifies irreducible information via Kullback–Leibler (KL) divergence between whole-pattern and quadrant-partitioned distributions (Equation (4))

(4) $$\varphi^{*}(multifractal)=I-I^{*}$$

where I = integrated information of the whole multifractal pattern; I* = information when spatial quadrants are treated as independent parts; φ*(multifractal) = irreducible information existing only in spatial inter-quadrant relationships, quantified via KL divergence. (See Supplemental Methods Section S3.4 for full computational implementation).

KL divergence was chosen as the measure of φ*(multifractal) because it forms the mathematical foundation of early IIT formulations, where it calculated integrated information φ by comparing whole-system distributions against independently partitioned components ([36]; see Supplemental Methods Section S3.4 for full implementation details). This approach mirrors φ_dyn_ from Parts 1–2: both adopt the integration-versus-partition framework of IIT but apply it to different domains. While φ_dyn_ measures temporal integration across evolving network connectivity, φ*(multifractal) measures spatial integration within the somatic substrate, capturing how φ*(multifractal) changes after each update cycle and reflecting consciousness as an ongoing process rather than a fixed state. Recent theories emphasize molecular substrates in Layer 5 pyramidal neurons and somatic integration as critical for consciousness, including Dendritic Integration Theory (DIT; [64] and Orchestrated Objective Reduction (Orch OR; [75]). However, these theories have not specified a mechanism for information integration. RIFT proposes multifractal spatial encoding as the mechanism. Together, φ_dyn_ and φ*(multifractal) reveal that consciousness is informationally irreducible at both the network and molecular levels, a convergence that scalar approaches to somatic integration cannot capture.

Beyond compression, GFM enables a representational architecture in which contradictory states coexist. Classical feedback systems overwrite old states with new ones. GFM instead preserves compressed traces of prior states within the fractal structure through seed extraction, allowing past and present information to occupy mutually embedded subspaces. GFM implements differential encoding: the seed carries compressed historical context (q fraction of prior information), while new EPSPs contribute only *delta information*, what has changed. This weighted integration M(t) = q·Seed(t − 1) + (1 − q)·ΔEPSP(t), with q = 0.3 in baseline simulations, structurally resembles Bayesian updating through geometric pattern combination rather than probability calculus. This updating mechanism provides a substantial compression advantage: encoding complete cortical states requires 100–1000:1 compression, while encoding deltas as the amount of information updated in each GFM cycle requires only 10–100:1 per cycle (see Supplemental Methods Section S3.3 for the Bayesian analogy and full compression analysis). Beyond compression, fractal embedding as in GFM enables contradictory states to coexist without forcing premature binary resolution, a property formalized as the fractal gate mechanism ([76]; see Supplemental Methods Section S3.5).

This architecture has direct consequences for conscious experience. The traffic light that turns green is recognized as a change precisely because the prior red state is preserved as a compressed trace in the current multifractal; the whole-in-part property makes temporal continuity intrinsic to the substrate rather than requiring separate memory systems. When GFM is disrupted, the inability to relate present to past breaks down episodic memory, as observed in frontotemporal dementia and Alzheimer’s disease.

Taken together, Parts 1–3 resolve two of the core challenges identified in the Introduction. The neural degeneracy problem is addressed through multi-level fractality: fractal dendritic geometry preserves positional information (Part 1), φ_dyn_ dynamics distinguish patterns with identical static integration (Part 2), and multifractal spatial organization retains irreducible φ*(multifractal) that scalar summation eliminates (Part 3). The extreme compression paradox, comparable in magnitude to the ~10^8^-fold gap between sensory input and behavioral throughput documented across a century of measurements [5], is addressed through differential encoding in GFM, where fractal embedding preserves temporal context through the whole-in-part property despite million-fold compression. Part 3, however, has established only the theoretical and computational framework; the biological substrate that physically implements this framework remains to be identified. Part 4 identifies this substrate: an Ising lattice of ion channels and lipid domains in the somatic plasma membrane that realizes the multifractal encoding GFM requires.

***Part 4:*** 

**Somatic Information Integration—Membrane Lipids as the Primary Substrate for Neural Information Processing**

In principle, a GFM substrate is not confined to the cell membrane: any molecular agent with intrinsic fractality through clustering can serve, including the cytoskeleton/tubulin clusters [77], calcium waves/ion clusters [78], or water shells/hydration clusters as proposed by others [79]. However, the unique arrangement of ion channels embedded into lipid domains in the somatic plasma membrane, and their direct responsiveness to EPSP trains, makes them the optimal substrate for implementing GFM. This arrangement is an Ising lattice in which both components exhibit intrinsic fractal properties and interact reciprocally: EPSPs display power-law temporal dynamics and scale-invariant behavior; membrane lipids self-organize into fractal spatial clusters at biologically relevant scales [80]. The cell membrane morphology is measurably fractal from 0.2 to 5 μm, with fractal dimension correlating quantitatively with membrane capacitance [81].

Critically, neuronal membrane lipid composition is actively regulated to sit close to its phase transition temperature, placing the Ising lattice near the critical point at physiological temperature (37 °C). At this critical point, long-range correlations emerge and small perturbations can propagate globally, a scale-free spatial organization that RIFT requires for Self-attractor formation [82,83]. EPSPs activate voltage-gated ion channels through membrane depolarization, while lipid rafts modulate channel conductance and gating kinetics. In turn, membrane depolarization induces lipid reorganization, and reorganized lipid domains modulate subsequent channel responses [84,85], a reciprocal dynamic that enables multifractal pattern self-organization without preconfigured geometry.

Part 3 mapped EPSP amplitudes onto the somatic multifractal using a Sierpinski-carpet subdivision rule, a fixed nested-grid geometry suited to the idealized 243 × 243 lattice of the theoretical model (see Supplemental Methods Section S3.2). Part 4 instead uses Gaussian kernel mapping, a continuous spatial transformation that better approximates how depolarization actually decays with distance from each synaptic input source (see Supplemental Methods Section S4.1).

**Figure 9:**
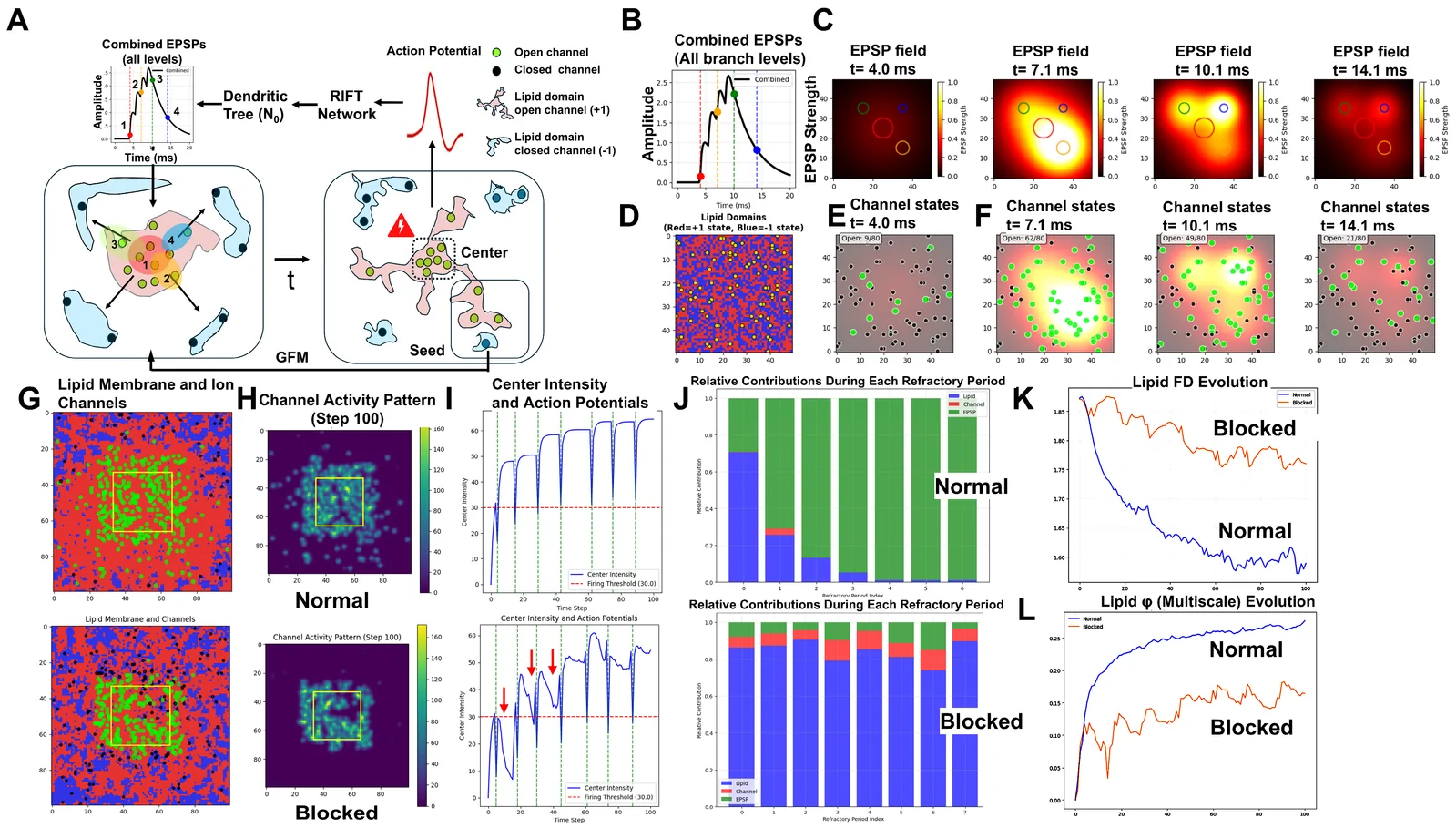
Biological multifractal implements lipid-mediated information integration with EPSP programming. (**A**) GFM cycle in core neuron: hierarchical EPSP inputs program lipid domain organization, driving action potential generation and seed extraction for recurrent loop formation. (**B**) Combined EPSP temporal profile (peak amplitude ~2.3 at 9 ms). (**C**) EPSP field spatial distribution (Gaussian kernel) at four timepoints (t = 4.0–14.1 ms), showing field decay and spatial reorganization. (**D**) Initial lipid state (t = 0): uniform binary distribution (~50% red/blue). (**E**,**F**) Ion channel states at four timepoints: progressive channel activation following EPSP dynamics. (**G**,**H**) Lipid and channel activity heatmaps at step 100: continuous mode shows distributed activity; blocked mode shows central concentration with phase-separated peripheral domains. (**I**) Center intensity and action potentials for continuous vs. blocked conditions: blocked mode shows progressive baseline rise (~30 to ~35 over 100 steps) and peak-decay-refocus pattern (arrows), demonstrating lipid memory accumulation. (**J**) Relative contributions per refractory period: EPSPs dominate in continuous mode (~85%); lipids dominate in blocked mode (~85–95%). (**K**) Lipid FD evolution: blocked condition maintains elevated FD > 1.75 vs. normal decline. (**L**) Lipid φ evolution: normal ~0.25–0.30, blocked ~0.10–0.15. Lattice: 100 × 100 (0.25 µm/pixel); ~400 ion channels; n = 100 steps.

The biological GFM cycle begins with EPSPs from proximal to distal branches converging at the soma (Figure 9A, left panel: pre-collapse state, lipid domains in red/blue with embedded channels green = open/black=closed, concentrated activity at center). EPSP amplitudes from each dendritic branch level are converted to spatially distributed membrane depolarization fields via this Gaussian kernel mapping, with proximal branch level 1 positioned at the lattice center and distal levels at increasing radial distances (Equation (S10); Figure 9B,C, Code 10; Supplemental Methods Section S4.1), preserving dendritic hierarchy in spatial form. Against this initial lipid domain background (Figure 9D), the field progressively activates ion channels through lipid-mediated coupling (Equation (S11); Figure 9E,F), where opening probability depends on local lipid state and cooperative interactions among neighboring open channels. Once EPSPs program initial channel states, Ising interactions govern lipid domain reorganization into spatially structured domains, lipid state +1 associated with open channels and −1 with closed. These binary lipid states directly modulate subsequent channel opening probabilities, driving center-focusing and threshold accumulation (see Supplemental Methods Section S4.2). The cycle proceeds analogously to Part 3: EPSP-driven programming, autonomous lipid growth toward firing threshold, collapse and action potential generation (Figure 9A, right panel), and peripheral seed preservation. Critically, the seed is not an abstract pattern excerpt but a physically instantiated molecular memory: lipid states, unlike channel states, are not reset at action potential generation, allowing organized peripheral domain configurations to survive collapse and carry compressed information into the next generation.

In addition to intrinsic fractality, any biological GFM substrate requires temporary shielding against unwanted EPSP updating to evolve its fractality autonomously. Two modes of EPSP integration were compared to test autonomous lipid contribution directly (see Supplemental Methods Section S4.3). The continuous mode delivers EPSP trains throughout the refractory period (unshielded control), while the blocked mode delivers EPSPs only at refractory onset. After that point, channel opening (Equation (S11)) loses its EPSP-driven term and is governed instead by local lipid bias and channel cooperativity alone. Lipid states continue to evolve through Ising-governed Metropolis updates that are themselves biased toward the state of nearby open channels (Supplemental Methods Section S4.3 for details). In blocked mode, channels and lipids continue to co-evolve, but channel activity becomes a downstream readout of lipid state rather than an independent driver, which is why pattern development in this mode is attributed primarily to lipid dynamics. The two modes produce distinct substrate organization at step 100 (Figure 9G): continuous mode shows a red-dominated lipid center with heterogeneous periphery, while blocked mode shows larger, more uniform peripheral domains with clear phase separation, reflecting unconstrained Ising relaxation. Channel activity heat maps (Figure 9H) mirror this: continuous mode shows distributed activity across the lattice, while blocked mode shows a concentrated central hotspot. Center-intensity traces (Figure 9I) show sustained activity tracking ongoing EPSP drive in continuous mode, versus a peak-decay-refocus pattern in blocked mode (arrows) as the system rebuilds autonomously between inputs. The blocked-mode baseline rises progressively across the 100-step simulation, the observable signature of accumulating lipid memory. Quantifying relative EPSP, channel, and lipid contributions to center-intensity change confirms this (Figure 9J): EPSP input dominates in continuous mode (~85%), while lipid contributions dominate in blocked mode (85–95%), with channels serving as readout elements rather than independent information integrators in either condition.

Because lipid domains, not ion channels, drive multifractal evolution in this substrate (Figure 9J; Supplemental Methods Section S4.3), the fractal and information-integration analysis (Supplemental Methods Section S4.4) is reported for the lipid substrate. Lipid domain fractal dimensions remain substantially elevated under blocked EPSP conditions (FD > 1.75), declining more rapidly under continuous input (FD ≈ 1.6). The φ*(lipid) measure extends the integration-versus-partition principle of Parts 2–3 to the molecular level, using a multiscale spectral-connectivity-mutual-information measure (Supplemental Methods Section S4.4 for details). It shows substantial integration in both conditions (continuous: 0.224–0.237; blocked: 0.123–0.136), maintained even during autonomous periods without EPSP input. The convergence of φ_dyn_ (network timing), φ*(multifractal) (somatic spatial pattern), and φ*(lipid) (molecular substrate) on the same integration-versus-partition principle provides multi-level evidence that consciousness is informationally irreducible at every scale of its biological implementation, not merely at the network level as IIT assumes.

Lipid domain configurations persist on millisecond-to-second timescales, far slower than channel state transitions, carrying compressed pattern information across generational boundaries and enabling temporal continuity of conscious states through molecular memory rather than requiring persistent neural firing patterns. This time frame also allows the shielded evolution of the multifractal in the (EPSP) blocked mode, a prerequisite for unfolding the experiential endospace and autopoietic feedback modulating the multifractal independently of newly incoming EPSP trains. Part 5 now addresses how this lipid-channel multifractal substrate generates the holographic endospace and implements autopoietic feedback.

***Part 5:*** 

**The Autopoietic Self-Attractor—Consciousness Shapes Its Own Neural Network Through Geometric Field Dynamics and Holographic Endospace**

**Figure 10:**
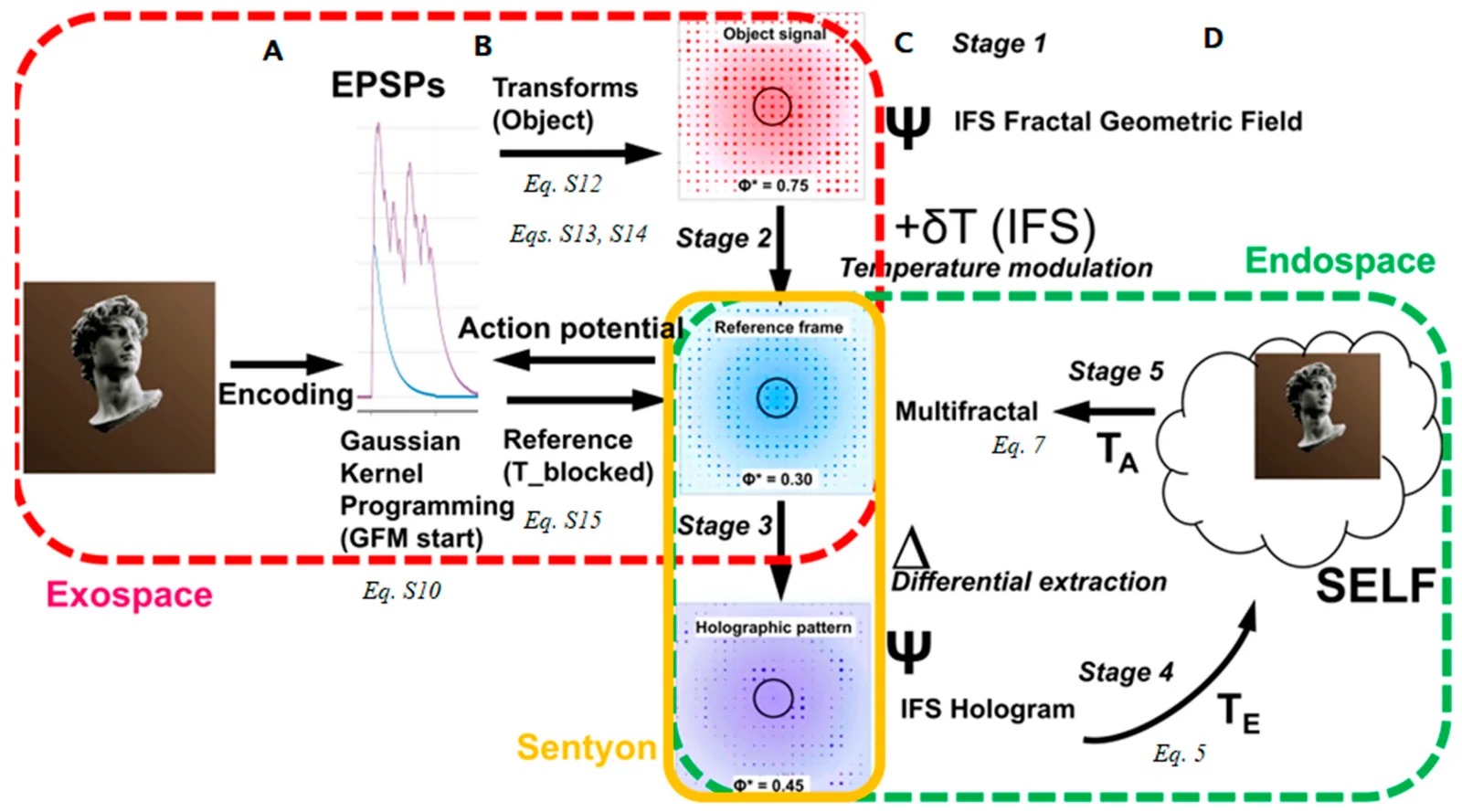
Five-stage holographic encoding of consciousness from exospace to endospace and autopoietic control. (**A**) Exospace encoding: an object is mapped into a temporal EPSP waveform and a Gaussian-kernel spatial field that initiates GFM (Equation (S10)). (**B**) IFS transform extraction generates the object signal and blocked-condition reference frame (Equations (S12)–(S15)). (**C**) Stages 1–3: IFS field generation, temperature modulation, and differential extraction produce the holographic pattern (φ* = 0.75, 0.30, and 0.45, respectively). The yellow border identifies the sentyon. (**D**) Stages 4–5: the IFS hologram reconstructs the endospace through T_E, and endospace-derived feedback through T_A modulates the multifractal substrate. Border colors: red = exospace, yellow = sentyon, green = endospace.

Parts 3 and 4 established how temporal EPSP sequences generate spatial multifractal patterns in the somatic lipid-channel lattice, encoding consecutive conscious moments through GFM. Part 5 addresses two central mechanistic questions: how this molecular substrate generates the endospace as a spatiotemporal reference frame, and how geometric field parameters derived from endospace can modulate the substrate. RIFT proposes that the construction of endospace and feedback from its geometric field to the molecular substrate are linked within a unified architecture in which fractality and holographic encoding perform complementary, mutually necessary functions. Fractality organizes the substrate for whole-in-part Self-mapping, while holographic reconstruction unfolds that organization as a unified spatial field. The fractal Self-attractor thereby serves as the geometric source structure from which the 3D endospace is holographically projected. Parameters derived from the resulting field modulate the lipid-channel substrate through the proposed autopoietic feedback pathway. This *biological holography* operates through molecular dynamics and geometric phase relationships rather than laser interference, extending Gabor’s foundational concept of holographic memory in the brain [86,87] to concrete molecular implementation through Iterated Function System (IFS) geometric transforms [88,89]. The multifractal enfolding (exospace-to-multifractal, Step 1), holographic unfolding and projection (multifractal-to-endospace, T_E_, Step 2), and autopoietic feedback (edospace-to-multifractal, T_A_, Step 3) proceed through five stages as depicted in Figure 10:

### 3.2. Stage 1: Multifractal Encoding Through IFS Geometric Transforms

**Figure 11:**
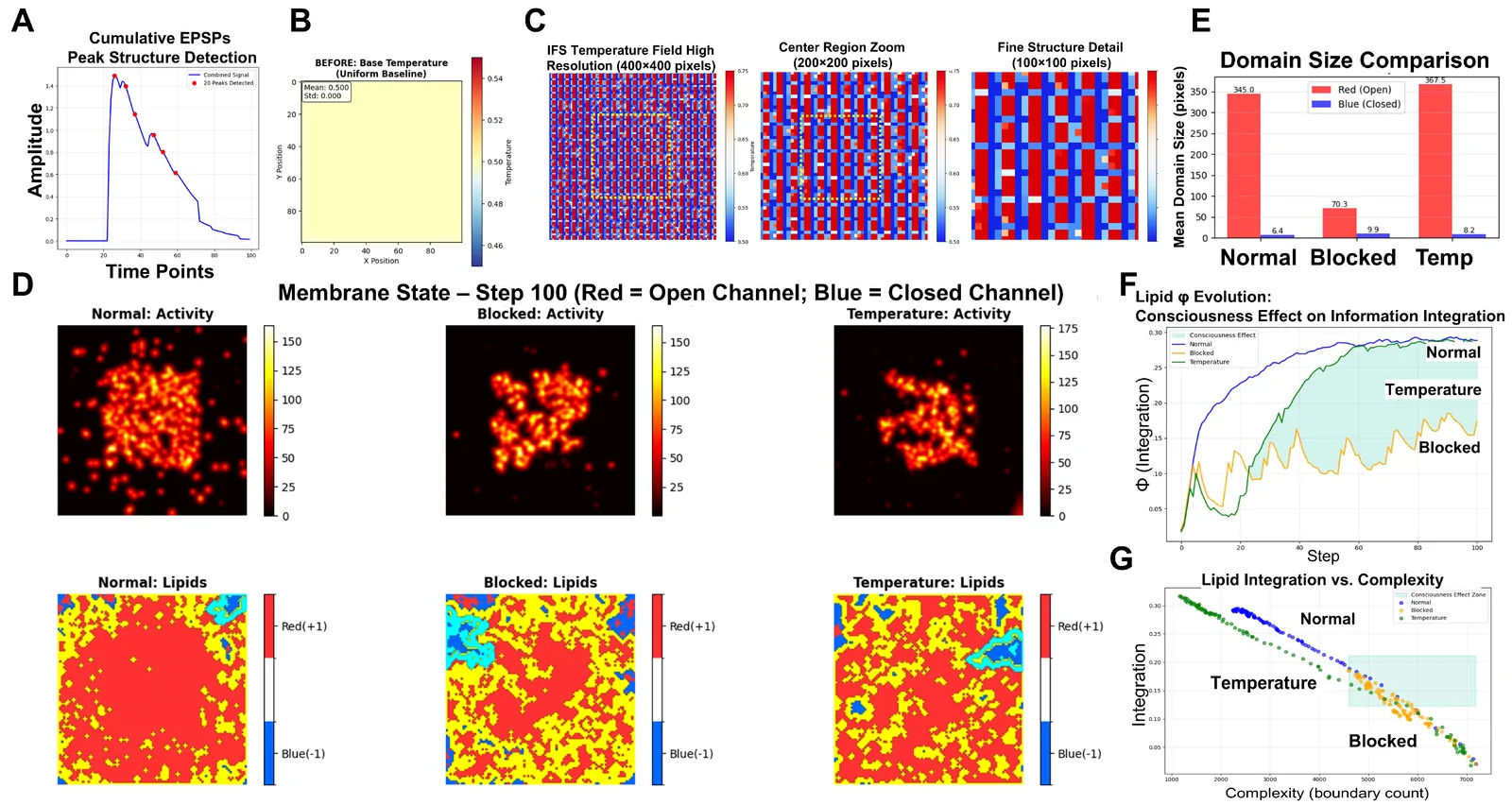
IFS temperature field modulates lipid reorganization and enhances information integration. (**A**) EPSP amplitude peak detection: hierarchical peaks reflecting r^(i−1)^ fractal scaling across branch levels. (**B**) Uniform baseline temperature field (T = 0.50, σ = 0.000) before IFS application. (**C**) IFS temperature field after chaos game iteration: ~20–30 fractal hotspots (T ~0.52–0.54) within cool matrix (T ~0.46–0.48), encoding EPSP hierarchical information at molecular scales; zoom panels show nested structure across three spatial scales. (**D**) Membrane states at step 100 under Normal, Blocked, and Temperature conditions: activity maps (top) and corresponding red/blue lipid-domain patterns (bottom). Normal produces broad central activity and lipid consolidation, Blocked produces more compact activity and fragmented domains, and Temperature partially restores domain organization. (**E**) Domain size comparison: Normal ~345 pixels, Blocked ~70.3 pixels, Temperature ~367.5 pixels. (**F**) Lipid φ evolution: Normal and Temperature both plateau ~0.29, converging by step ~60; Blocked plateaus ~0.15–0.20. Shaded region marks the Consciousness Effect (Temperature ≥ Blocked). (**G**) Integration vs. complexity; Consciousness Effect Zone (teal box) spans complexity range of Blocked mode, height = mean Temperature − mean Blocked integration (~0.12–0.21). Lattice: 100 × 100; T_base = 0.5, IFS_strength = 0.2.

In Stage 1 (Figure 10A,B), EPSPs serve a dual purpose at each GFM cycle: they program the daughter fractal substrate through Gaussian kernel mapping (Part 4), and simultaneously provide geometric field information through IFS extraction (Equation (S12); see Supplemental Methods Section S5.1 for details; Figure 11A shows EPSP amplitude peaks used for both). This dual role is not an arbitrary design choice but a structural necessity: the geometric/phase information IFS extraction requires exists only in the amplitude and timing pattern of arriving EPSPs. Once the refractory period begins, no further EPSPs arrive. Lipid self-organization and ion channel interactions are then governed entirely by energy conservation and Ising dynamics, which can propagate existing structure but cannot generate new positional information. If IFS extraction were deferred to occur after refractory onset, it would have no EPSP data left to extract from during that window. Extraction must therefore occur concurrently with substrate programming, capturing the geometric information the autonomous dynamics cannot supply on their own.

To model the IFS extraction, we first run Codes 11 and 12 to initiate the biological multifractal as in Part 4. In Code 14, EPSP amplitude hierarchies are then converted into six affine geometric transforms encoding the spatial relationships of dendritic integration, scaling, rotation, and translation components that collectively capture the hierarchical structure of synaptic input (Figure 10, Stage 1; see Supplemental Methods Section S5.1 for details on Code 14 and IFS extraction). These IFS transforms then generate a spatially heterogeneous temperature field across the somatic membrane through chaos game iteration implemented in Codes 15 and 16 (Equation (S13); see Supplemental Methods Section S5.2 for the equation and code details). Chaos-game iteration, rather than the fixed positional mapping used for Gaussian kernel programming (Part 4), was chosen because self-similarity across scales is intrinsic to an IFS attractor. The resulting temperature field injects EPSP-derived geometric information into the existing lipid pattern at multiple scales simultaneously, rather than writing to fixed regions that then evolve independently under local Ising dynamics. This achieves whole-in-part encoding of the current moment into the prior substrate configuration, the same principle that motivated the Sierpinski carpet geometry in Part 3 (see also Supplemental Methods Section S3.2), something Gaussian kernel programming cannot accomplish on its own. This same persistence gives the temperature field a temporal role as well. Once computed, it continues biasing lipid reorganization for the remainder of the refractory period rather than acting as a single instantaneous stamp. The temperature field functions as an echo of the originating EPSP pattern that integrates the current moment with the ongoing evolution of the multifractal. This is quantified in Stage 2 by the intermediate φ*(lipid) levels it produces relative to the Blocked and Normal conditions. Figure 11B,C shows the temperature field before (B) and after (C, Code 17) modulation by the IFS transforms; in C, warm regions correspond to areas the chaos-game iteration visits most frequently, reflecting where the EPSP-derived geometric transforms concentrate.

### 3.3. Stage 2: Holographic Pattern Recording in the Lipid Membrane

In Stage 2 (Figure 10C), this IFS-derived temperature field drives lipid domain reorganization during the refractory period through thermodynamic modulation of the Metropolis algorithm (Equation (S14); see Supplemental Methods Section S5.3 for details). EPSP information is integrated twice at this point, distinguishing the three model conditions. Gaussian kernel programming of the substrate occurs in all conditions (Part 4); while, IFS-derived temperature modulation of lipid reorganization probability occurs only in the Temperature condition; the Blocked condition omits both forms of renewed EPSP input and so provides the autonomous baseline against which Temperature-driven reorganization is measured. Higher local temperatures in this IFS-structured field (Figure 11C) allow greater lipid reorganization flexibility, encoding EPSP hierarchical information through spatially patterned phase transitions, directly paralleling how a reference beam modulates photographic media in optical holography.

The final evolved lipid domain pattern constitutes the recorded hologram (Figure 11D, Code 18). This record is the sentyon [6], the molecular substrate that carries the geometric imprint of the conscious moment. Critically, because total energy is conserved during autonomous evolution, the IFS-modulated probability field alters lipid domain distributions without violating energy conservation, the same principle that makes autopoietic control physically viable. Figure 11E (Code 16) quantifies this effect: the Normal model (continuous programming with new EPSPs) produces a mean red domain size with large central hotspot (Figure 11D, left panel), the Blocked model (no new EPSPs during refractory period) produces smaller, fragmented domains (Figure 11D, middle panel), and the Temperature model (new EPSPs blocked, but temperature modulation through IFS from initial EPSP) produces partially consolidated lipid domains (Figure 11D, right panel), demonstrating the reorganizing effect of the temperature field. Beyond holographic recording, reinjecting EPSP information during the refractory period increases information integration in the multifractal: Figure 11F (Code 13, analysis) shows Temperature converging with Normal (~0.29) by step ~60, while Blocked plateaus lower (~0.15–0.20); Figure 11G quantifies this as the Consciousness Effect Zone, the mean integration gain of Temperature over Blocked (~0.12–0.21) across the complexity range of the Blocked mode.

### 3.4. Stage 3: Holographic Differential Extraction to Generate the Self-Attractor

**Figure 12:**
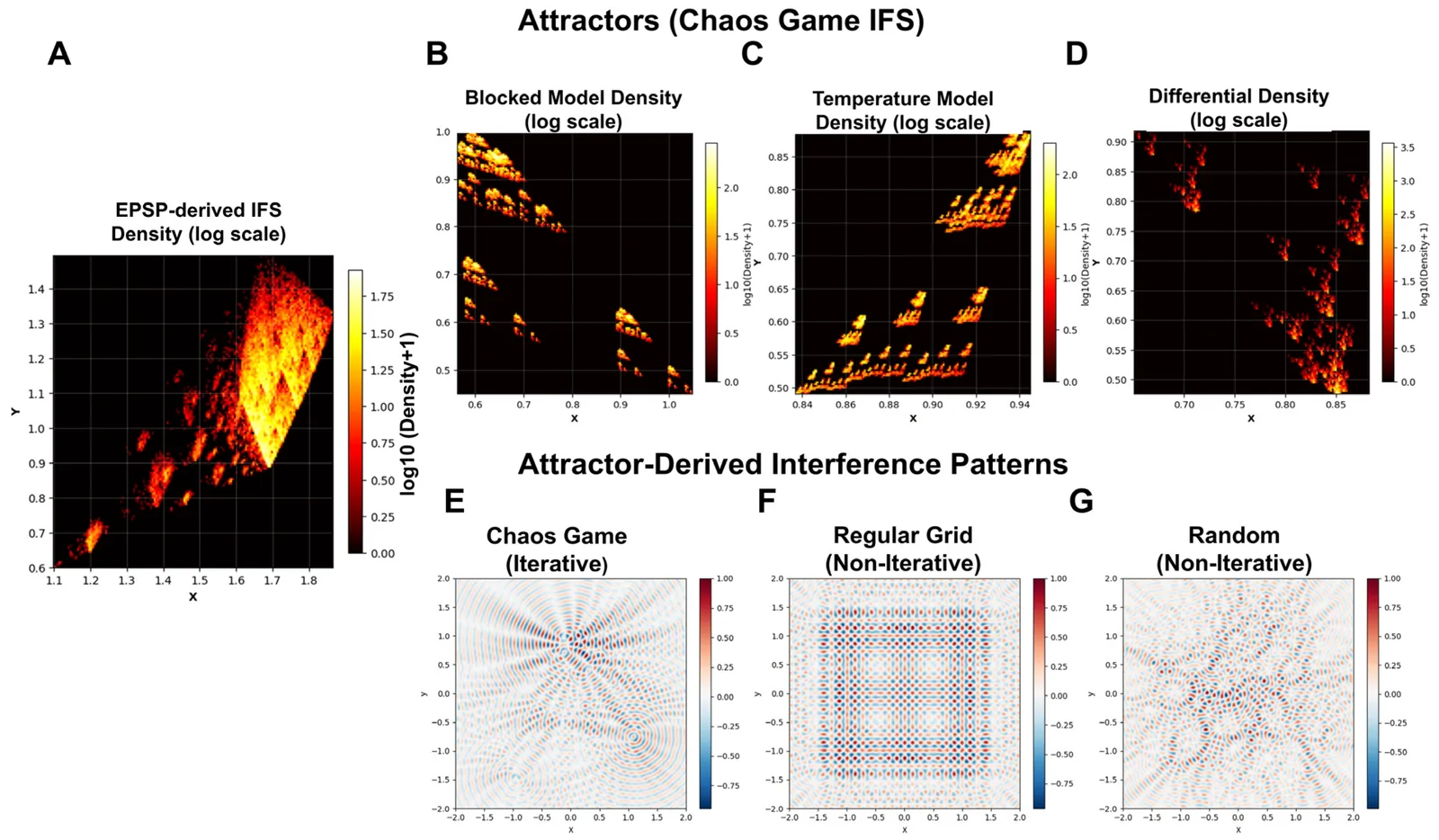
Differential IFS processing generates regionally distinct attractors and coherent interference patterns. (**A**–**D**) IFS attractor densities in transform parameter space: (**A**) EPSP-derived, (**B**) Blocked model, (**C**) Temperature model, and (**D**) differential H_RIFT = T_temperature − T_blocked. (**E**–**G**) Interference patterns comparing source positioning: (**E**) chaos-game iteration, (**F**) regular grid, and (**G**) random placement.

In Stage 3 (Figure 10C), the differential transforms to generate the Self-attractor are extracted from the evolving multifractal. Figure 12A–D visualizes IFS attractors generated by chaos-game iteration for four transform sets (Code 21): the original EPSP-derived transforms from Code 14 (Figure 12A), and the transforms extracted from the evolved lipid pattern under Blocked (Figure 12B) and Temperature (Figure 12C) conditions. After evolution of the lipid pattern, the pure holographic signature is recovered by subtracting the IFS transforms extracted from the autonomously evolved (blocked) lipid pattern (Figure 12B) from those of the IFS-modulated (temperature model) pattern (Figure 12C), yielding the differential transforms H_RIFT (Equation (S15); see Supplemental Methods Section S5.4 for details; Figure 12D). This differential extraction, implemented in Codes 19 and 20, isolates the geometric information imposed by EPSP encoding beyond autonomous baseline dynamics, the biological equivalent of removing the reference beam to reveal the object signal (see Supplemental Methods Section S5.4 for details).

### 3.5. Stage 4: The Self-Attractor and Holographic Projection of the Endospace

In Stage 4 (Figure 10D), the differential IFS transforms H_RIFT (Figure 12D; Stage 3) generate the fractal Self-attractor through chaos game iteration (Code 21; see Supplemental Methods Section S5.5 for details), positioning coherent point sources whose phases are derived from geometric relationships to IFS transform centers weighted by transform determinants. The original EPSP-derived attractor (Figure 12A) provides a baseline for comparison. The Self-attractor is the geometric organization of point sources in transform space; whereas, endospace is the unified field that these sources collectively generate through interference. Their relationship is analogous to that between the recorded interference pattern of a hologram and the 3D image it reconstructs. Through fractal enfolding, the Self is mapped onto every subspace of the somatic multifractal through the whole-in-part organization of the Self-attractor. Holographic projection unfolds this distributed organization as endospace. The perceiving Self is therefore intrinsic to the same recursively maintained organization that generates endospace, rather than a separate observer or homunculus to whom the projected field is displayed. The Self-attractor and endospace are complementary aspects of this organization: the geometric source structure and the geometrically structured domain of first-person experience generated from it. Holographic reconstruction preserves exospace topology and whole-in-part unity within endospace.

Coherence of the reconstructed endospace depends critically on how interference sources are positioned. Chaos game iteration on H_RIFT places sources such that spatially proximate sources share correlated phases, creating phase gradients that encode the hierarchical organization of EPSP temporal patterns in the spatial geometry of the endospace. This is demonstrated in Figure 12E–G: chaos game positioning produces coherent concentric interference structures consistent with Gabor zone plate patterns (Figure 12E; [90,91], while regular grid placement (Figure 12F) and random placement (Figure 12G) produce incoherent patterns with destructive interference. Holographic projection is therefore the principled choice for endospace reconstruction (T_E_) rather than an arbitrary one: wave interference preserves spatial phase relationships by construction, guaranteeing that the reconstructed endospace retains the geometric structure of the multifractal substrate regardless of which physical process implements the projection:

(5) $$(T_{E}\;\mathrm{transformation}):\;\Psi (x,y,z)=\sum_{i}\frac{A_{i}(H_{RIFT})\cdot exp[i\cdot (k_{i}\cdot r_{i}+\varphi_{i})]}{1+{r_{i}}^{2}}$$

where A_i_(H_RIFT) = amplitude from differential determinant values at source i, derived from the H_RIFT transforms of Stage 3; k_i_, r_i_, φ_i_ = wave number, transform-space distance, and coherent phase for source i, with the 1/(1 + r_i_^2^) term implementing distance-dependent decay (see Supplemental Methods Section S5.5 for further details).

**Figure 13:**
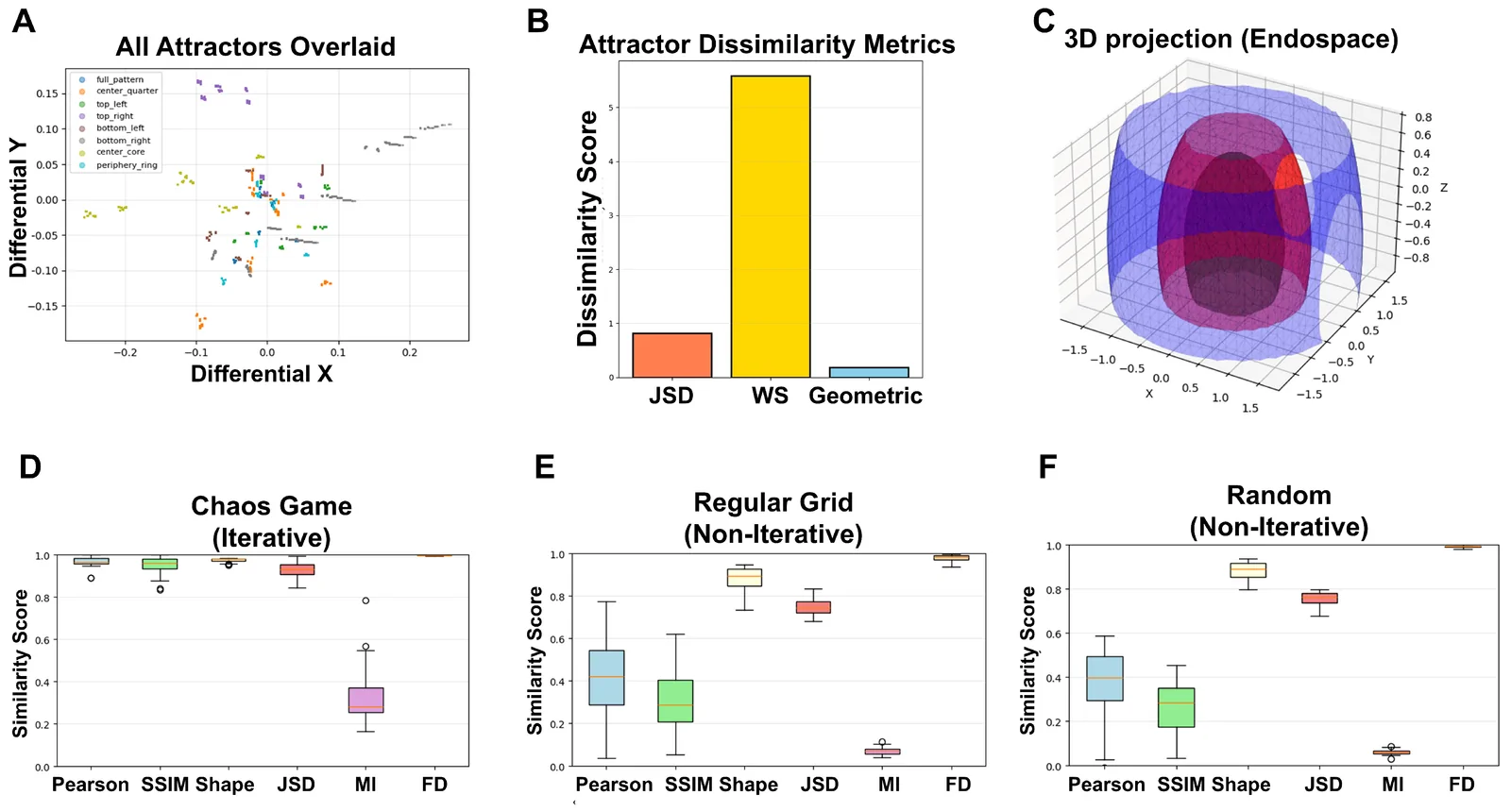
Holographic reconstruction preserves whole-in-part organization in endospace. (**A**) Eight regional differential attractors overlaid in transform space. (**B**) Attractor dissimilarity metrics for the regional attractors. (**C**) Three-dimensional endospace field Ψ(x, y, z). (**D**–**F**) Endospace similarity distributions across eight regions for (**D**) chaos-game, (**E**) regular-grid, and (**F**) random source positioning.

**Figure 14:**
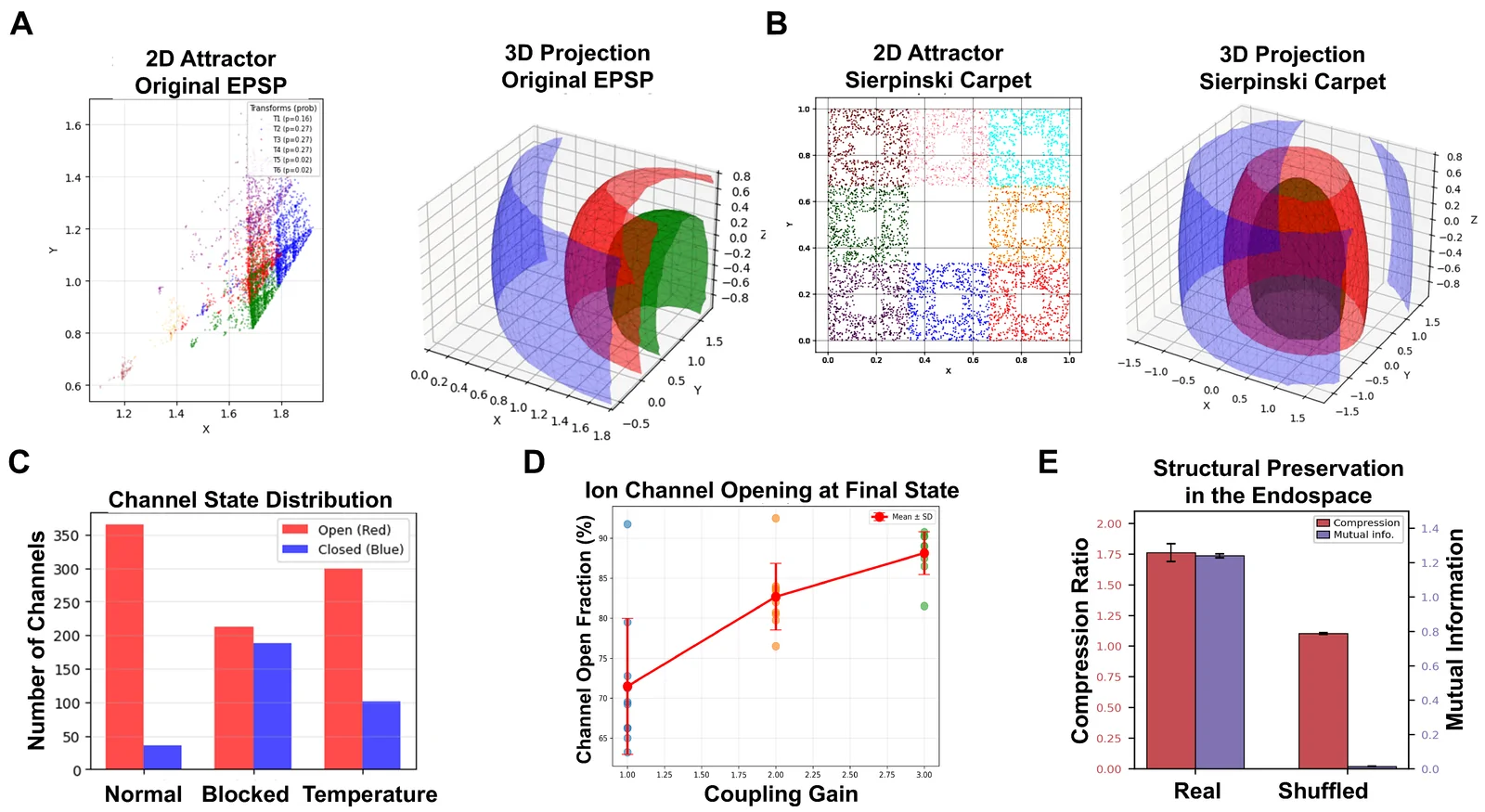
Construction controls, structural preservation, and coupling-dependent membrane responses. (**A**,**B**) Non-differential reconstruction controls using (**A**) the initial EPSP-derived attractor and (**B**) a generic Sierpinski-carpet multifractal, each shown as a two-dimensional attractor and three-dimensional projection. (**C**) Channel-state distribution under Normal, Blocked, and Temperature conditions. (**D**) Ion-channel opening as a function of coupling gain γ. (**E**) Structural preservation in endospace compared with a positionally scrambled control.

The holographic whole-in-part property, that each fragment of the Self-attractor encodes the complete conscious experience, is validated by applying this reconstruction to eight distinct spatial regions of the somatic multifractal separately. Regional attractors extracted from these eight regions (full pattern, center quarter, quadrants, center core, periphery) are geometrically dissimilar from each other: overall dissimilarity = 0.50 (mean of Jensen–Shannon divergence and geometric feature dissimilarity; see Supplemental Methods Section S5 for the full metric set), Jensen–Shannon divergence = 0.82 (Figure 13A,B, Code 24). Yet when chaos game-positioned sources from each regional attractor are used for holographic reconstruction via Equation (5) (Figure 13C), all eight regions produce highly similar 3D endospace fields (overall similarity = 0.96, a weighted composite of six independent metrics including Pearson r = 0.97 and SSIM (structural similarity index) = 0.96; see Supplemental Methods Section S5.5 for details on dissimilarity and similarity measures; Figure 13D–F). This 50% dissimilarity yielding 96% reconstruction similarity is the defining signature of holographic whole-in-part encoding. Just as a fragment of an optical holographic plate, despite being dissimilar to other fragments, reconstructs the complete image when illuminated, each regional fragment of the Self-attractor encodes the complete conscious experience. The 3D endospace field Ψ(x, y, z) (Figure 13C, one representative region shown) establishes the spatiotemporal dimension in which inner experience unfolds. This whole-in-part property is specific to chaos game positioning on H_RIFT: regular grid and random source placement produce lower and more variable inter-regional similarity scores (Figure 13E,F, Code 25), confirming that both the differential IFS transforms and the chaos game iteration are jointly necessary for coherent holographic encoding. The initial EPSP-derived attractor and a generic Sierpinski carpet multifractal, positioned with the same chaos game projection but lacking the differential H_RIFT transforms, both fail to achieve whole-in-part encoding (similarity below 0.70 vs. 0.96 for H_RIFT in Figure 12A, Figure 13D and Figure 14A,B), confirming both components are jointly necessary. The regular-grid, random-position, non-differential-attractor, generic-fractal, and positional-scrambling comparisons serve as construction controls within the present model. They show that high regional reconstruction similarity is not obtained with arbitrary source placement, non-differential attractors, or generic fractal inputs. These controls test whether the result is a generic consequence of source positioning or fractality within the implemented model; they do not establish that the proposed reconstruction occurs biologically.

### 3.6. Stage 5: Holographic Autopoietic Control Through Ψ Feedback

In Stage 5 (Figure 10D), the T_A_ transformation implements the endospace-to-membrane return step of the proposed autopoietic loop (Equations (6) and (7); see also Supplemental Methods Section S5.6 for details on equations and Code implementation). Within the RIFT architecture, this return pathway is required for consciousness to be causally efficacious rather than epiphenomenal. We extract the regional mean endospace intensity $\bar{\Psi }$ (psi-bar; Code 26) separately for each reconstructed region as the spatial average of its field magnitude:

(6) $$\bar{\Psi }=\left\langle \left|\Psi (x,y,z)\right|\right\rangle$$

where $\bar{\Psi }$ is the spatial average of the endospace field magnitude within each reconstructed region, extracted from the holographic projection of Equation (5).

This mean-field approach does not require point-by-point mapping of the 3D field back to the 2D membrane. Instead, the regional endospace state tunes the probabilistic landscape within which channel–lipid interactions unfold. In RIFT, this low-dimensional signal represents the direction or strength with which the current experiential state biases a subsequent Go/No-Go decision, such as whether to enter the coffee shop described in Figure 1. Therefore, $\bar{\Psi }$ does not encode the entire content of conscious experience. The structured content remains in endospace; whereas, the scalar conveys its net influence on the relevant substrate.

We propose that the mean field intensity $\bar{\Psi }$ feeds back to modulate the coupling strength γ (determines how sensitive ion channels respond to lipid states) between ion channels and the temperature-organized lipid substrate:

(7) $$\left(T_{A}\;\mathrm{transformation})\;\gamma (t)=\gamma_{0}(1+\alpha \cdot \bar{\Psi }(t)\right)$$

where α represents the feedback strength and $\bar{\Psi }$(t) is the mean endospace field intensity from the current conscious moment. γ scales the weight of the local lipid domain state in the opening probability calculation for each channel (Equation (S11)): at γ = 1 (baseline), lipid state contributes ±0.25 to the sigmoid input of each channel; at higher γ, this contribution scales proportionally, making organized lipid domains increasingly determinative of channel behavior relative to direct EPSP input (see Supplemental Section S5.6 for further explanation of α and γ).

The channel-opening fraction (Figure 14C) serves as the primary readout of the membrane response required for the proposed autopoietic pathway because the model isolates the refractory-period lipid-channel dynamics following action-potential generation from the subsequent integration of new EPSPs with the preserved seed. This allows systematic examination of how coupling modulates membrane-state reorganization between action potentials without confounding from rapid EPSP-driven reprogramming. Systematic variation in coupling strength (γ = 1.0–3.0, n = 10 trials per condition) confirmed dose-dependent sensitivity to coupling strength (Figure 14D), with the channel-opening fraction increasing by 6.5 percentage points at maximum coupling (95.5% versus 89.0%). Temperature fields remained independent of coupling strength, confirming that variation in γ selectively affected coupling-dependent channel behavior without altering the IFS-derived temperature fields. The 6.5-percentage-point increase was obtained by setting γ directly across a predefined range, demonstrating the sensitivity of channel opening to lipid-channel coupling strength. Within RIFT, this manipulation models the proposed downstream effect of experience-dependent modulation of γ; it does not simulate the emergence or qualitative content of experience itself. In the Equation (7) feedback route, γ is instead calculated from the endospace-derived $\bar{\Psi }$. Thus, the manual sweep validates the downstream membrane response to γ but does not directly execute the complete $\bar{\Psi }$-to-γ transformation.

Probability modulation, here of ion channel opening, offers a physically plausible mechanism through which consciousness could influence matter without directly injecting energy into the system. This strategy has been proposed across theoretical frameworks from quantum approaches [92,93] to classical stochastic accounts grounded in ion-channel thermal noise [94], and it is given concrete biological grounding by recent demonstrations that lipid composition introduces probabilistic complexity into ion channel voltage sensitivity [84]. In RIFT, consciousness does not micromanage individual channel–lipid interactions but modulates lipid-channel coupling strength, thereby tuning the probability distribution from which channel states emerge.

The complete information flow through this architecture reveals an important design principle (Figure 14E): a compact generative code, six IFS transforms with seven parameters each (42 real numbers; Figure 12A), expands through holographic reconstruction into a 6912-voxel endospace field (Figure 13C). This expansion reflects genuine coherent structure, not merely increased voxel count: the reconstructed field shows substantially higher spatial redundancy than a positionally scrambled control with identical intensity values (mean compression ratio ~1.76 versus ~1.10; neighbor-to-neighbor mutual information ~83-fold higher, across 8 regions). These spatial relationships survive reconstruction but collapse under shuffling, showing that informational richness lies in how values are organized, not in voxel count alone. From each reconstructed regional field, only a single scalar $\bar{\Psi }$ (Equation (6)) is extracted for autopoietic feedback. Within the implemented Equation (7) mapping, this low-dimensional variable is sufficient to modulate γ without requiring fine-grained remapping of endospace content. The extension of this influence between brain regions is addressed by the sentyon-transfer mechanism in Part 6. The low-dimensional character of this control signal is consistent with the approximately 10 bits/s bandwidth of conscious perception [5], although this correspondence remains a plausibility argument rather than a derived quantity.

Within RIFT, the Self-attractor emerging from whole-in-part encoding is proposed as the invariant geometric structure underlying a unified subjective perspective. It cannot be divided while maintaining its identity, exhibits self-similarity across scales, and emerges dynamically from neural activity rather than existing as a preformed structure. Recursive self-reference through holographic reconstruction establishes the whole-in-part organization required for this unified perspective, with each local subspace of the multifractal encoding information about the complete system pattern. Field-derived feedback then modulates membrane excitability and provides the proposed route to downstream effects on recurrent network dynamics. Within the RIFT architecture, this autopoietic return pathway gives the physically realized endospace causal efficacy rather than leaving consciousness epiphenomenal. The sentyon-transfer mechanism examined in Part 6 proposes how the molecular representation of the Self and the endospace it experiences can be transferred between brain regions.

***Part 6:*** 

**Sentyon Cloning and Wandering Mind—Fractal Transfer of the Dynamic Core in the Global Workspace of the Brain and its Disruption in Alzheimer’s Disease**

**Figure 15:**
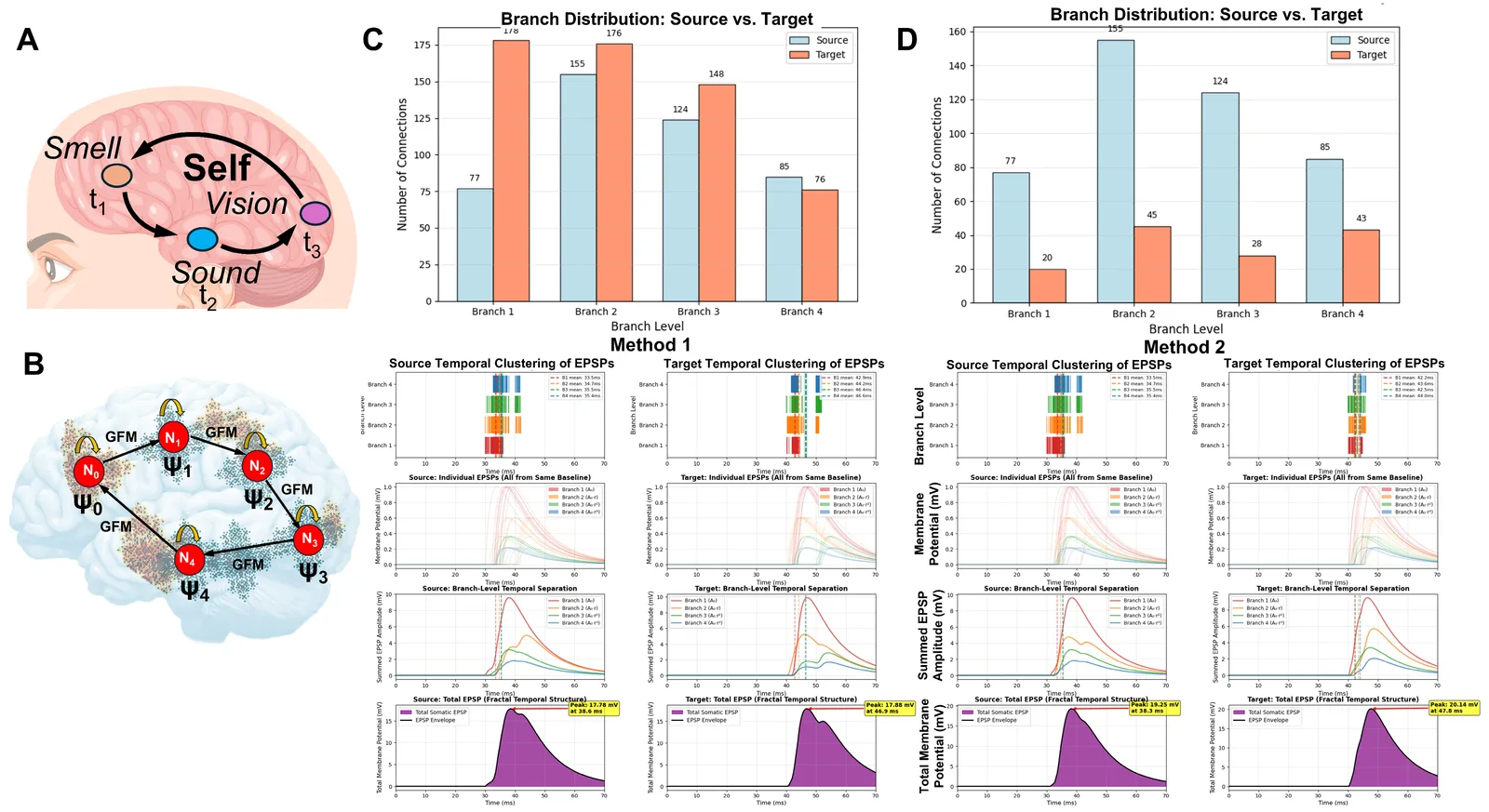
Sentyon transfer enables consciousness migration and compares two connectivity-transfer methods across core neurons. (**A**) Self-transfer across sensory modalities: integration of smell (t_1_), sound (t_2_), and vision (t_3_) streams into unified conscious experience. (**B**) Distributed GFM architecture: sentyon patterns (Ψ_0_–Ψ_4_) propagating between core neurons (N0–N4) through GFM seed transfer. (**C**) Method 1 (External Position Copying): branch distributions and EPSP temporal structures of the source and target networks. (**D**) Method 2 (Synaptic Position Copying): branch distributions and EPSP temporal structures of the source and target networks. Method 2 establishes fewer connections but preserves the source EPSP distribution more closely than Method 1 (95.0% versus 91.9% similarity).

Any localized model of consciousness faces a fundamental challenge: how can a substrate confined to individual core neurons integrate the vastly distributed information available to consciousness? RIFT addresses this through sentyon mobility, the proposed capacity of the conscious substrate to transfer between neurons rather than requiring all distributed information to converge on a single fixed locus. The same GFM mechanism that preserves temporal continuity within a neuron (Parts 3–4) also enables seed transfer between neurons: the compressed multifractal seed of the current conscious moment can be cloned into a target core neuron, entraining its network activity and establishing a new locus of conscious integration (Figure 15A,B). RIFT proposes that the same autopoietic control governing the multifractal through the Self in endospace would also enable reconstruction and integration of a local RIFT network after Self-transfer to a different brain region. Rather than a static central processor, consciousness becomes a wandering dynamic core that travels to wherever relevant processing occurs, cumulatively integrating visual, auditory, tactile, and other sensory modalities across successive locations (Figure 15A,B).

This wandering mind mechanism implements Edelman’s dynamic core hypothesis [44] through a concrete molecular mechanism that the original “bright spot” concept lacked. It is also compatible with Global Neuronal Workspace Theory (GNW; [10]), but with a critical difference: rather than broadcasting to a static workspace, RIFT proposes that the sentyon carries the workspace directly to each processing center. Prioritization is proposed to arise through the autopoietic loop; the present simulations test transfer to a specified target rather than the upstream selection of that target. Empirical observations consistent with dynamic conscious access include binocular rivalry, cross-modal attention shifts, sequential neural ignition, and default mode network dynamics, all of which indicate that conscious access is dynamically distributed rather than permanently confined to a fixed site [10,95,96,97].

**Figure 16:**
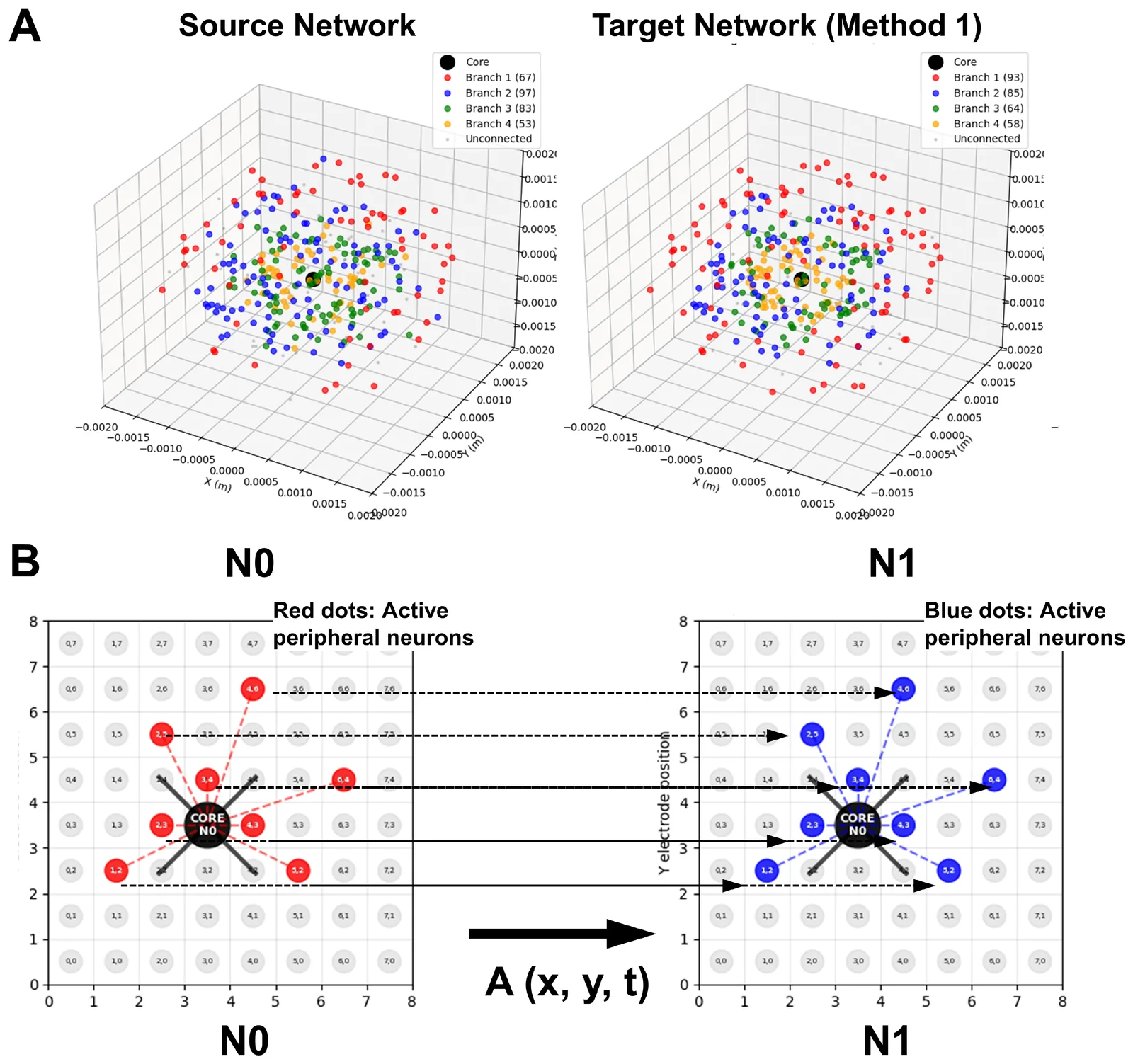
Network-level implementation of coordinate-based sentyon transfer. (**A**) Three-dimensional spatial arrangements of the source network and the target network generated by Method 1, with peripheral neurons color-coded by branch assignment. (**B**) Coordinate-based transfer from core neuron N0 to core neuron N1 through A(x, y, t), illustrating an MEA-compatible artificial implementation. Solid black lines mark direct core-to-electrode connections; dashed colored lines trace the coordinate mapping A(x, y, t) from each active peripheral neuron in N0 to its corresponding position in N1.

Computational simulations compared two methods for replicating connectivity from a source core neuron (Nt) to a target (Nt + 1), both implementing the RIFT time constraint rule. Method 1 copies the complete 3D spatial coordinates of all connected peripheral neurons; Method 2 transfers connectivity at the dendritic level by copying branch-level assignments and using coincidence detection to guide new connections. The results revealed a counterintuitive dissociation (Figure 15C,D; Codes 29–30, see Supplemental Methods Sections S6.1 and S6.2 for details): Method 1 achieved superior connection utilization but shifted the normalized EPSP amplitude distribution away from the source pattern when target networks operated at different somatic delays. Method 2 established fewer total connections but preserved branch-level proportionality more faithfully (95.0% vs. 91.9% EPSP distribution similarity; Figure 15D), maintaining the hierarchical amplitude structure essential for multifractal encoding. Both methods produced similar oscillation frequencies, confirming that sentyon cloning entrains target network activity to the source pattern regardless of copying strategy. Figure 16 shows the network-level implementation of external-position copying: panel A compares the source and target 3D networks, while panel B illustrates coordinate transfer between N0 and N1 in an MEA-compatible architecture.

In RIFT, this entrainment provides the proposed mechanism by which conscious control can extend to a new processing center. The simulations demonstrate target-network entrainment produced by the two connectivity-transfer strategies. Within the complete RIFT architecture, this entrainment is proposed to bring the target network under the same autopoietic loop that governs the originating neuron, rather than leaving it as an independently oscillating system that the Self must separately coordinate. Biological plausibility favors Method 2, as constraint-based synaptic development aligns with known mechanisms [98], while Method 1 is better suited to artificial implementations such as multielectrode array (MEA)-based neurochips where precise electrode positioning is achievable (Figure 16B). This suggests an implementation-dependent distinction between biological and artificial realizations of RIFT. Successful sentyon transfer predicts observable neural signatures: gamma oscillations (30–100 Hz, corresponding to GFM cycle frequencies) should show coherence across sequentially attended brain regions, while theta rhythms (4–8 Hz) should exhibit cross-frequency coupling with gamma bursts at each attended location [32,99].

RIFT proposes three pathways through which neurological disease could disrupt conscious experience: altered lipid composition impairing multifractal substrate formation, degraded EPSP hierarchies compromising seed extraction quality, and synaptic loss undermining seed transfer fidelity. The third pathway has a specific mechanistic implication: as synapse loss progresses, Method 2-like sentyon cloning becomes unreliable, preventing the conscious substrate from relocating effectively, potentially explaining the fragmented, discontinuous conscious experience of advanced dementia. Critically, AD pathology does not produce uniform synapse loss. Tau-driven spine loss occurs in clusters [100], dendritic disconnection proceeds asynchronously across the arbor [101], and the apical dendritic tuft, corresponding in the RIFT model to the highest branch levels (3–4) of the fractal hierarchy, shows preferential atrophy or complete loss in the majority of affected neurons [102]. This branch-selective pattern would selectively collapse the distal end of the A_0_·r^(i−1)^ amplitude hierarchy, corrupting fractal encoding in a way that uniform synapse loss would not. This is a novel mechanistic prediction of RIFT that to our knowledge has not yet been directly tested.

## 4. Discussion

### 4.1. Consciousness, Agency, and the Role of the Self—Novelty of RIFT

RIFT offers a testable framework grounded in geometric principles that produce the defining features of consciousness—irreducibility, information integration, holographic encoding, and autopoiesis—through computationally evaluated candidate mechanisms rather than imposed constructs. The convergence of φ_dyn_, φ*(multifractal), and φ*(lipid) on fractal organization at network, somatic, and molecular scales provides multi-level evidence for informational irreducibility throughout the biological implementation, not merely at the network level as IIT assumes.

The step-wise fractal enfolding of exospace information into the somatic substrate (Step 1), followed by holographic unfolding into the endospace (Step 2), provides a candidate mechanism for generating an experiential space topologically accurate to the exospace, rather than assuming one. Holographic unfolding is driven by coherent point sources organized through the whole-in-part property of the Self-attractor, providing the physical condition for topologically faithful reconstruction. None of the comparison frameworks specify both a substrate that preserves relational structure and a mechanism that reconstructs it as experiential geometry: bulk electromagnetic fields such as CEMI lack coherent point sources, IIT specifies no reconstruction step, and FEP and GNW propose no projection mechanism for an experiential space. Where these accounts assume the correspondence between inner and outer space, RIFT derives it from mechanism. RIFT therefore addresses a generative question distinct from the hard problem of why the reconstructed geometry is experienced. Clinical dissociations illustrate that experiential geometry and its qualitative contents are distinguishable. In cerebral achromatopsia, chromatic experience can be lost while the spatial organization of the visual scene remains largely preserved [103]. Conversely, in Alice in Wonderland syndrome, perceived size, distance, body schema, or temporal relationships can diverge from unchanged external geometry [104]. Within RIFT, the latter distortions would be interpreted as disruptions of the exospace-to-endospace transformation T_E_. Together, these observations demonstrate that qualitative experience and its organization within a spatiotemporal reference frame can be differentially disrupted. Therefore, the generative mechanism for constructing the endospace as the spatiotemporal reference frame for inner experience is one of the core contributions of RIFT.

Generating a geometrically structured endospace addresses the construction of experiential space; the next question is how experience within that space acts back on its physical substrate. The identity claim in IIT gives integrated information no route back onto the substrate that generated it; the broadcast mechanism in GNW explains access to consciousness, not how a felt state alters neural dynamics [105]; FEP grounds action selection in surprise minimization without tying it to a geometric inner space. The T_A_ transformation in RIFT is proposed to close this loop directly: mean endospace intensity $\bar{\Psi }$ modulates ion-channel–lipid coupling strength γ, a quantified, dose-dependent pathway (Part 5) requiring no new forces or violation of energy conservation. This proposes autopoietic control, and by extension volition, as a mechanistic consequence of the same architecture that generates the endospace, rather than as a separate postulate. Suppressing an impulse, noticing the aroma of the coffee shop, feeling the urge to stop, but deciding there is not enough time (Figure 1), corresponds to the Self-attractor selectively reducing coupling strength γ in the networks representing the impulse while sustaining it in the networks representing the prior commitment. The moment of deliberation itself, holding both the impulse and the commitment in balance before one prevails, is formalized as the Contradiction Ratio (CR, see Supplemental Methods Section S3.5 for further discussion), which quantifies exactly this balance between competing state representations.

This framing has implications for the relationship between consciousness and attention. In RIFT, conscious attention is not a separate supervisory system but an expression of sentyon mobility (Part 6): the Self-attractor relocates to processing centers where integration is needed, entraining their network dynamics through seed transfer. Selective attention, maintaining focus on a task while suppressing irrelevant inputs, corresponds to the Self-attractor sustaining coupling strength in task-relevant networks while withholding it from others. The wandering mind is not a failure of attention but the natural expression of a mobile Self navigating its distributed neural substrate.

However, the present model does not simulate how the Self selects which content receives attentional priority. Holistic reconstruction and selective attention are distinct: holistic reconstruction preserves the relational unity and topology of endospace; whereas, selective attention determines which content and processing center guide subsequent activity. RIFT proposes that this selection directs relocation of the sentyon to the relevant neuron or network through autopoietic feedback, but the current simulations do not model the selection process itself.

RIFT addresses the integration of the Self with endospace, but the present implementation does not formalize the multimodality of experience or the episodic memories of which the Self is aware. Episodic Memory Theory relates conscious experience to the binding of multimodal features into coherent episodic representations [106], while Feature Integration Theory assigns attention a central role in binding features into objects [107]. These theories, together with IIT [7,36], GNW [9,10], and FEP [12,13], provide accounts of neural information integration, access, attention, memory, or action. However, they do not specify a physical transformation by which encoded relationships are reconstructed as the geometry of endospace. The distinct contribution of RIFT is the proposed transformation T_E_ for this generative step and the transformation T_A_ through which the Self acts back upon its physical substrate. The present implementation models the reconstruction of spatial relationships within endospace. How interoceptive, affective, mnemonic, and abstract contents are encoded within this geometry has not yet been formalized or simulated. Table 1 compares these RIFT-specific proposals with alternative frameworks.

### 4.2. Limitations of RIFT

The assumptions in RIFT about recursive, timing-based connectivity remain anatomically unproven, though evidence from hippocampal circuits [108] and thalamocortical loops [109] supports their plausibility. The biological plausibility of the somatic multifractal, while supported by known lipid-channel interactions [110,111], requires experimental validation through high-resolution membrane imaging combined with measures of conscious state. Distance and time scales used in the simulations fall within the range of known biological phenomena, providing a basic realism check.

This hierarchical amplitude scaling describes organization within a single core neuron: how EPSP amplitudes are structured across dendritic branch levels converging on one soma, and makes no claim about the neural code used across populations of neurons upstream of that soma. Whether those upstream neurons represent information through strict hierarchical tuning or through mixed selectivity is a separate question at a different level of description, and the mechanism of RIFT is agnostic between them. Part 5 only requires that the EPSP train arriving at the core neuron soma retains sufficient hierarchical amplitude structure, regardless of the coding scheme that produced it upstream.

A deeper challenge is the localization of consciousness to specific core neurons. Unlike theories distributing consciousness across brain-wide electromagnetic or quantum fields, RIFT places conscious experience in specific neuronal compartments, raising the question of how a confined substrate integrates the vast distributed information underlying experience. Global field theories avoid this bottleneck but lack empirical support and clear mechanisms: brain-wide electromagnetic fields do not achieve the coherence and stability required for holographic encoding [112,113], and quantum theories face decoherence timescales incompatible with biological conditions [114,115]. Panpsychism faces the combination problem: if individual atoms generate separate endospaces, no mechanism exists for their integration into unified experience [116,117].

The localization challenge is addressed by GFM-based sentyon mobility: through coincidence-based synaptic selection, coherent EPSP trains recreate complete multifractal patterns at different core neurons, allowing consciousness to remain locally instantiated while dynamically relocating. This makes the localization challenge testable rather than fatal: if GFM enables multifractal transfer, synthetic systems such as brain–computer interfaces or organoid cultures should exhibit detectable transition signatures when the pattern of one core neuron reconstructs at another site.

A related question is localization not across neurons but within one: the soma-centered architecture is itself a substantive choice. An earlier, dendrite-centered alternative, closer to the original RFNN formulation [20,21], with precedent in Woolf’s dendritic encoding hypothesis [118] and Laberge and Kasevich’s apical dendrite theory [119], remains compatible in principle with continuous-downscaling generation of the Self-attractor, a core mechanism in RIFT. Dendritic integration would keep synaptic-location readout and integration in the same place, but at the cost of separating integration from the site of action potential generation and autopoietic feedback. In addition, it faces its own unresolved binding problem, since it is unclear how per-branch integration would achieve unified rather than per-branch experience, part of the original motivation for the soma-centered approach adopted here.

The holographic reconstruction of endospace is the most theoretically ambitious component of RIFT. It addresses a fundamental gap left by major theories: where and how the geometrically structured domain of first-person experience could be physically instantiated. RIFT proposes that IFS-based holographic reconstruction from lipid-channel multifractals generates geometric fields that constitute endospace (T_E_), which in turn feeds back to modulate the substrate through autopoietic control (T_A_). IFS-based holographic reconstruction extends a body of work exploring the significance of fractality and holography for consciousness [120,121,122,123], as noted above. A more recent proposal in the same vein frames lipid membranes, vicinal water, and cerebrospinal fluid as a holographic substrate for consciousness [124], but it does not specify a generative process linking membrane structure to the holographic pattern it proposes. RIFT, in contrast, derives the Self-attractor and its holographic projection from a stated biophysical process (Part 1, Part 5). GFM also resolves a biological problem specific to holographic theories: how interference patterns persist given continuous lipid turnover or other dynamic metabolic processes. A compressed seed extracted at each GFM cycle actively regenerates holographic memory without requiring static lipid storage, while lipid domain configurations constitute a molecular memory that accumulates across cycles. Fractality provides the molecular memory that holography alone cannot supply; holographic projection provides the endospace generation that fractality alone cannot produce.

Several core operations, IFS transform extraction (Part 5, Stage 1), GFM seed extraction (Part 3, Code 9), and holographic projection of the endospace (Equation (5)), are best understood as computational proxies for functional outcomes the substrate must achieve, not as physical claims about how it achieves them. Relatedly, the EPSP trains driving multifractal programming in Part 3 (Code 9) are generated independently of the network simulation in Part 1 (Code 1) rather than inherited from it, hence network formation and multifractal encoding are not currently run as a single interdependent process. Each is well-motivated by what it accomplishes (whole-in-part encoding, cross-moment continuity, coherent reconstruction) but is computed externally and imposed on the model rather than emerging from membrane physics. Candidate physical mechanisms exist. Long-range correlations near the 37 °C critical point of lipid membranes [82,83], localized soliton-like conformational propagation confined to the somatic membrane [125], and coherent collective excitation of a bounded membrane patch [126] are theoretically proposed but not yet experimentally confirmed in biological membranes. These candidate mechanisms and the proposed somatic multifractal have not yet been tested as biological implementations of RIFT. This is not a failure specific to RIFT so much as the general limitation of any theory formalizing consciousness computationally ahead of the relevant biology. Resolving it is deferred to the experimental program below, once measurements of real membrane dynamics can adjudicate which mechanism, if any, the model should adopt. The goal of the present model is to reconstruct spatial relationships within endospace as the spatiotemporal dimension of inner experience. How interoceptive, affective, mnemonic, and abstract contents are encoded within endospace has not yet been formalized or simulated.

A continuously coupled end-to-end implementation also remains future work. Its construction will require validated intermodule interfaces, a shared time base, feedback-stability testing, and parameter-sensitivity analysis before conclusions about full-system behavior can be drawn. A minimal integrated demonstration would pass actual Part 1 EPSP output into Part 3, carry the resulting multifractal through holographic reconstruction, derive feedback from endospace, and apply that feedback to the same network over multiple cycles. Development of this integrated computational pipeline will be addressed in future studies together with comparisons between the RIFT network and a matched nonfractal dendritic architecture or conventional integrate-and-fire network. Such comparisons are required to determine which effects are specifically attributable to fractal architecture rather than to recurrent dynamics or scalar integration more generally.

### 4.3. Experimental Validation and Future Directions

#### RIFT-Specific Falsifiable Predictions

RIFT presents multiple pathways for experimental validation. Initial testing is more feasible in synthetic systems than in humans: brain–computer interfaces, organoid models, neuromorphic hardware exhibiting scale-free, brain-like criticality [127], or closed-loop culture systems such as those developed by Cortical Labs [128] can be engineered with recursive connectivity, coincidence-based synaptic selection, and monitored lipid-channel organization. If the RIFT model is correct, stabilization of a multifractal pattern in one core neuron should be followed by its reconstruction in another, with detectable transition signatures in lipid domain reorganization and electrical activity. Microelectrode arrays with precise electrode positioning could implement Method 1 sentyon cloning, potentially enabling higher-fidelity information transfer than biological synaptic development allows.

The empirical predictions of RIFT are testable with current techniques: (1) disruption of lipid domain organization through cholesterol depletion or membrane fluidizers should impair consciousness more than ion channel blockade alone, consistent with the temperature-criticality argument developed in Part 4; (2) fractal dimension of neural activity should correlate with conscious state depth, decreasing during anesthesia and deep sleep; (3) inverse timing relationships should exist between dendritic branch levels and external network paths in recurrent circuits; (4) temporal desynchronization should alter conscious content while spatial pattern disruption affects conscious level. A further RIFT-specific prediction is that transfer of a compressed multifractal seed should produce measurable translation and entrainment signatures across processing centers.

Data in the literature offer preliminary support: fractal scaling in neural oscillations changes systematically during sleep, anesthesia, and disorders of consciousness [129,130], and fractal dimension decreases during deep sleep and anesthesia [131]. These observations are consistent with the prediction that fractal organization is necessary for consciousness. Whether neuronal network activity exhibits genuine geometric fractal organization, as opposed to scale-free statistical scaling alone [132], remains an open empirical question at the network level. The predicted fractal dimension at the molecular substrate provides a specific, falsifiable answer.

At a more speculative level, the holographic mechanism proposed for RIFT converges with recent work approaching consciousness from fundamental physics rather than biology. Awret’s analysis of holographic correspondence in consciousness [133] argues, from an independent physics-based starting point, for the same core idea developed here, that inner experience corresponds to a boundary-encoded reconstruction. Pursuing this convergence is a direction for future theoretical work, including whether principles from fundamental physics can inform the biological mechanism proposed by RIFT.

The RIFT model emerged through a novel methodology: rather than imposing mathematical frameworks and seeking correlates, theory construction was inverted, starting with a three-decade hypothesis about fractality, iteratively implementing mechanisms, validating against functional requirements, and extracting mathematics from working systems. This human–AI collaborative approach is itself a methodological contribution: future theories with different mathematical foundations can adopt the same strategy of grounding theoretical development in working computational models rather than untestable speculation.

### 4.4. Implications for Alzheimer’s Disease

RIFT offers novel perspectives on Alzheimer’s disease (AD), recently recognized as a disorder of consciousness whose impairment has been hiding in plain sight behind cognitive and behavioral symptom framing [134]. The pathological hallmarks of AD, amyloid plaques, tau tangles, synaptic loss, and membrane lipid dysregulation [135], directly target the molecular substrates RIFT identifies as essential for consciousness. Amyloid beta binds to sphingolipid domains [136], potentially disrupting multifractal structure formation. Ceramide generated by Aβ-induced sphingomyelinase activation disrupts sphingomyelin-cholesterol lipid rafts; this can be prevented by functional inhibitors of acid sphingomyelinase [137,138], suggesting a pharmacological pathway to preserving membrane-based conscious processing. Depression in AD may reflect not only altered input from emotional processing centers but also more generalized effects on neuronal membrane lipids critical for endospace generation.

AD pathology disrupts all three GFM-dependent processes: lipid raft disorganization impairs multifractal substrate formation [139]; synaptic dysfunction degrades the temporal precision of EPSP convergence required for fractal pattern formation [140]; and non-uniform synapse loss selectively collapses the distal end of the A_0_·r^(i−1)^ amplitude hierarchy, corrupting fractal encoding in a way that uniform synapse loss would not (Parts 5 and 6). When GFM fails to properly encode and transfer multifractal seeds, temporal continuity breaks down and the patient experiences discrete moments without connection to previous states, manifesting as anterograde amnesia. Because sentyon transfer (Part 6) extends conscious access across neurons by entraining target networks to the source multifractal pattern, disruption of this transfer between regions encoding different modalities would dissociate rather than uniformly degrade conscious perception. RIFT predicts that fractal complexity decline precedes overt cognitive decline, strongly supported by systematic FD reduction in AD patients’ EEG patterns and dendritic morphology [25,141], and that lipid domain disruption should correlate with episodic memory impairment more strongly than total amyloid burden.

### 4.5. Artificial Consciousness and AI Safety

RIFT provides specific structural criteria for assessing whether artificial systems could develop consciousness. Current AI systems, regardless of computational power or behavioral sophistication, lack the recursive fractal architecture RIFT identifies as necessary: fractal timing hierarchies, multifractal spatial organization, and holographic reconstruction mechanisms are absent from feedforward and simple recurrent networks. Artificial consciousness becomes possible if systems implement recursive networks with fractal branching and timing relationships, substrates capable of multifractal pattern formation (through analog computing, neuromorphic chips, or organoid-based neurochips), and mechanisms for holographic encoding and autopoietic feedback. The mathematical structure of RIFT is substrate-independent and does not preclude quantum implementations.

RIFT provides objective structural markers, multifractal organization, pattern transfer signatures, fractal dimension correlations, that go beyond behavioral self-report [142]. This enables deliberate rather than accidental development of conscious AI, providing engineering targets for systems intended to avoid consciousness and monitoring protocols for systems that might inadvertently develop it. The critical distinction is that conscious systems can genuinely reconsider what goals mean through experiential integration rather than applying pre-programmed rankings [143]. Whether this flexibility is beneficial or dangerous depends on what values such systems develop by observing us, a question that makes understanding and detecting consciousness not merely scientifically interesting but urgently practical.

## 5. Conclusions

RIFT proposes a three-step mechanism through which recurrent neural activity is transformed into a unified endospace that can act back upon its physical substrate. Fractal enfolding compresses network activity into a somatic multifractal while preserving whole-in-part organization. Holographic unfolding reconstructs the encoded spatial relationships as endospace geometry, and autopoietic feedback modulates the molecular substrate from which that geometry arose. Across six computational parts, the simulations demonstrate dynamic information integration, incremental updating through Generational Fractal Mapping, whole-in-part holographic reconstruction, field-dependent modulation of channel opening, and transfer of the Self-attractor between processing centers.

The central contribution of RIFT is the specification of $T_{E}$, the physical transformation that reconstructs exospace relationships within endospace, and $T_{A}$, the transformation through which endospace feeds back upon its substrate. The simulations test the generation and topological preservation of endospace geometry; they do not explain why this geometry is experienced. RIFT therefore provides an experimentally testable framework with the potential to advance a mechanistic understanding of consciousness. Its specific predictions concern recurrent timing, somatic lipid-domain organization, conscious-state-dependent fractal dynamics, and compressed-seed transfer. Future studies can test these predictions, validate the proposed biological substrates, and connect the computational modules into a continuously coupled pipeline, thereby advancing the present architecture toward a unified physical model of consciousness.

## Figures and Tables

**Table 1:** Comparison of RIFT to existing consciousness frameworks.

Framework	Shared Concern	Distinction from RIFT	RIFT-Specific Target
IIT	Integration and irreducibility	RIFT uses model-specific dynamical and substrate measures and does not calculate formal IIT Φ.	Physical generation of topology-preserving endospace.
GNW	Distributed access and coordination	RIFT proposes Self-attractor transfer rather than workspace ignition.	Transfer and reconstruction through fractal seed propagation.
FEP	Recurrent regulation and action-related feedback	The present RIFT model measures prediction-related dynamics but does not implement predictive inference or free-energy minimization as the mechanism generating endospace.	Geometric reconstruction and endospace-to-substrate feedback.
Pribram’s holonomic theory	Distributed and holographic representation	RIFT proposes explicit IFS-based reconstruction and molecular feedback.	A computational transformation from multifractal source organization to endospace.
Autopoiesis	Circular organization and self-maintenance	RIFT proposes a quantified field-to-substrate transformation.	Closure of the proposed endospace-substrate causal loop.

## Data Availability

The original contributions presented in this study are included in the article/Supplementary Materials. Further inquiries can be directed to the corresponding author.

## Supplementary Materials

The following supporting information can be downloaded at: https://www.mdpi.com/article/10.3390/brainsci16090983/s1.
