# Supplementary material for: RIFT: A Fractal-Holographic Theory of Consciousness and Autopoietic Control
Source: Brain Sci. 2026 Sep 16;16(9):983. doi: 10.3390/brainsci16090983 (PMC13604132; doi:10.3390/brainsci16090983)
Supplement: Supplementary file 1 [file brainsci-16-00983-s001.zip › brainsci-4514995-supplementary.pdf]

brain sciences

MDPI

Supplemental Methods Sections

All simulation codes (Codes 1-31) are archived on Zenodo (https://doi.org/10.5281/zenodo.22707705).. The Zenodo archive also includes a code reference table cross-listing equations implemented in each code, figures, and manuscript sections, along with a pipeline diagram of code execution order and dependencies. The following sections document implementation details for each Results Part.

Section S1: RIFT Network Architecture and Simulation - Part 1

S1.1 Network simulation (Code 1)

The RIFT network was implemented in Python (Code 1; Fig. 4). The core neuron N_0 was assigned a fractal dendritic tree with scaling factor r = 0.6 and fractal dimension FD ≈ 1.36 across 4 branch levels, with branch lengths following $L_n = L_0 \times 0.6$^(n−1) (Eq. 1), computed by the compute_dendritic_distances function. Synapses were distributed across branches proportionally to relative branch length by the distribute_synapses method, allocating synapses in proportion to total branch length at each level (branch length × number of branches), so that more distal levels with greater total dendritic surface received proportionally more synaptic contacts. Twenty peripheral neurons (N_i) were generated per firing cycle at randomized external distances within a predefined range. Network development across timesteps is shown in Fig. 4A-C; the inverse external distance-branch level relationship and emergent oscillation frequency are shown in Fig. 4D-G.

S1.2 Coincidence detection algorithm (Code 1)

The simulation detected coincidence by calculating whether EPSPs from multiple randomly positioned N_i would arrive simultaneously at the soma within a 2.0 ms temporal window (check_coincidence method). For each unconnected peripheral neuron N_i, total arrival time at the soma was computed as:

Eq. S3*: $T_{\text{arrival}} = T_{\text{external}} + T_{\text{internal}} + T_{\text{soma}}$

where T_external is the action potential travel time from N_0 to N_i and back, T_internal is dendritic signal propagation time from the synapse to the soma (branch_distance / v1), and T_soma is the somatic processing delay (5.0 ms in baseline simulations), directly implementing the time constraint rule of Eq. 3, the operational, three-term form of Eq. 3 actually computed by Code 1, with somatic processing delay added as an explicit term. Two detection modes were implemented: branch-preference mode (branch_preference=True, default, biologically recommended) required coincident EPSPs from at least 2 different dendritic branches within the same arrival window, emphasizing integration across the full tree geometry and enforcing the fractal geometry of the inverse timing relationship, and relaxed mode (branch_preference=False) counted total coincident signals regardless of branch origin, serving as a comparison condition. N_i-to-branch pairings producing coincident soma arrival were recorded as permanent recurrent connections, biologically corresponding to activity-dependent synaptic strengthening in which coincident pre- and post-synaptic activity determines which connections are retained. After a somatic processing delay, N_0 fired and initiated a new round of N_i generation, iterating until all synaptic sites were occupied. This temporal windowing is not merely a detection threshold, it is the mechanism that prevents scalar collapse: by treating only EPSPs arriving within 2.0 ms as belonging to the same coincidence event, the system preserves the identity of which branches fired together rather than summing signals across arbitrary time into a running total, which would reproduce the information-

destroying scalar integration of standard integrate-and-fire models. The same 2.0 ms value reappears as the temporal grain in the φ_{dyn} calculation (S2.1), where it defines the minimum separation between branch windows required for them to remain distinct integration epochs, connecting network formation directly to information integration measurement through a single architectural parameter.

S1.3 Fractal validation metrics (Code 1)

Emergent network fractality was quantified using three independent measures applied to the inter-spike interval (ISI) series derived from connected neuron external times (analyze_fractal_properties method; Fig. 4A, B): power-law distribution analysis (confirmed scale-invariant firing statistics), Higuchi Fractal Dimension (HFD, k-values 1-10), and Detrended Fluctuation Analysis (DFA, quantifying long-range temporal correlations through scaling exponents).

Power-law distribution analysis fitted a linear regression to the log-log histogram of ISIs, yielding a power-law exponent α and goodness-of-fit R^2; scale-invariant firing statistics consistent with fractal organization produce a linear log-log relationship across multiple time scales.

Higuchi Fractal Dimension (HFD) was computed from the ISI series using k-values 1-10, where k sets the interval lengths over which curve length is estimated; smaller k captures fine-grained temporal structure while larger k captures coarser scaling behavior. HFD was extracted from linear regression on the log-log plot of k versus normalized curve length, yielding HFD = 2 + slope.

Detrended Fluctuation Analysis (DFA) quantified long-range temporal correlations in the ISI series by computing root-mean-square fluctuations after linear detrending across logarithmically spaced window scales; a scaling exponent α > 0.5 indicates long-range correlations consistent with fractal dynamics.

All three metrics showed consistent increases in fractality over the course of network formation, confirming that coincidence-based synaptic selection drives the network toward scale-invariant temporal organization (Fig. 4A, B).

Eq. S1: $L_n = L_0 \times r^{(n-1)}$ [fractal branching rule]

where L_0 = primary trunk length; L_n = branch length at level n; r = scaling factor (r = 0.6 in standard models).

Eq. S2: $FD = \frac{\log N}{\log 1/r}$ [fractal dimension]

where N = number of branches; r = scaling factor. With r = 0.6, FD ≈ 1.36, within the empirical range of layer 5 cortical (FD = 1.24-1.33) and hippocampal pyramidal neurons (FD = 1.26-1.36).

Eq. S3: $T_{\text{total}} = T_{\text{internal}} + T_{\text{external}} = \text{constant}$ [time constraint rule]

where T_internal = dendritic signal propagation time to soma; T_external = action potential travel time from N_0 to N_i and return. Implemented in Code 1 via the check_coincidence method as Eq. 3* (S1.2), with coincidence detected within a 2.0 ms temporal window (see S1.2 for full implementation details).

Section S2: Information Integration Dynamics - Part 2

S2.1 φ_{dyn} calculation (Codes 1-2)

φ_{dyn} was calculated using the sliding window approach implemented in Codes 1-2 (PhiStarCalculator.calculate_phi_star method; Fig. 5A-D show branch-level firing window results). For each branch level, connected neurons N_i have characteristic external travel times that determine when their signals arrive at the soma; these times cluster into branch-specific temporal windows defined by the median external time ± 15% error margin (calculate_time_windows method). Windows falling within the 2.0 ms temporal grain of each other are merged, since signals arriving within this interval cannot be distinguished as temporally separate by the coincidence detection mechanism (S1.2); windows separated by more than 2.0 ms remain distinct, reflecting independent branch-level processing epochs.

Integration I is computed as the sum of exponential temporal couplings between consecutive distinct windows, and φ_{dyn} follows as the operational form of Eq. 5:

Eq. S5*: $\varphi_{\text{dyn}} = \max(0, I - I^*) \times \exp(-|\langle\Delta t\rangle - \tau|/\tau)$

where I = $\Sigma_i \exp(-|\Delta t_i| / \tau)$, Δt_i is the gap between the end of window i and the start of window i+1, and τ = 2.0 ms is the temporal grain (calculate_coupling method).

Partitioned integration I* is computed as the mean of within-branch coupling sums when each branch level is treated as an isolated partition, that is, coupling is calculated only between windows belonging to the same branch, and the mean across branches gives I*. φ_{dyn} (Eq. 5*) is then normalized by a separation factor $\exp(-|\text{mean\_separation} - \tau| / \tau)$, which peaks when the mean gap between windows equals exactly the temporal grain τ. This normalization implements the hill-shaped φdyn-delay relationship shown in Fig. 6: at window separations below τ, windows begin to merge and I collapses; at separations far above τ, coupling decays exponentially and I also falls; integration is maximized at the intermediate separation where windows remain distinct yet temporally coupled. The 2.0 ms temporal grain thus serves a dual role: it defines the coincidence window for synaptic selection in S1.2 and the optimal branch window separation for maximum φ_{dyn} in S2.1, linking network formation and information integration through a single architectural parameter. This identity is mechanistically grounded: the coincidence window defines the temporal quantum of discrimination below which signals cannot be distinguished as separate events. Branch windows separated by less than this quantum merge into a single integration epoch, collapsing φ_{dyn} toward zero; windows separated by much more than this quantum lose exponential coupling, also reducing φ_{dyn}. Maximum φ_{dyn} therefore occurs precisely when mean branch window separation approximates the coincidence window width, the somatic delay is the tunable parameter that positions the network at this optimum.

While φ_{dyn} adopts the conceptual framework of "integration exceeding partition," it does not implement formal φ calculation based on causal partitions as in IIT. It is presented as a biologically tractable proxy capturing temporal dynamics of integration not addressed by static causal analysis, following the sliding window method used in recent thalamocortical studies (Munn, Muller et al. 2023, Whyte, Muller et al. 2025) but adapted to quantify integration across dendritic branch timing patterns rather than probabilistic independence between neurons.

S2.2 Delay sweep analysis (Codes 3-7)

The delay-sweep analysis extended Code 1 via the run_improved_delay_sweep function in Code 3, systematically varying somatic delay from 1-20 ms with 5 repetitions per condition. Network parameters were configured via Code 4 (parameter setup and sweep execution script), which called Code 3 with r = 0.6, coincidence_window = 2.0 ms, firing_threshold = 2, and simulation_time = 200 steps, saving complete φdyn and fractal

metric time series to CSV for downstream analysis. Three predictability metrics were computed per delay per repetition and aggregated across repetitions (mean ± std):

Volatility: std(dφ_{dyn}/d(step)), measuring step-to-step variability in φ_{dyn} changes, computed from the numerical gradient of the φ_{dyn} series after optional Savitzky-Golay smoothing (window ≤ 11, polynomial order 2); time unit is per index step, not per ms, quantifying event-to-event variability independent of the delay-scaled time axis.

Prediction error: mean|φdyn(t+1) − φ_{dyn}(t)|, the mean absolute difference between consecutive φdyn values, serving as a simple state-transition prediction-error proxy related to, but not equivalent to, Fristonian surprise (Friston 2010).

Autocorrelation: Pearson r(φ_{dyn}(t), φ_{dyn}(t+1)), measuring how strongly the next state is predicted by the current state.

Volatility declined from 0.020 at 5 ms to 0.009 at 19 ms; prediction error declined 9-fold from 0.018 at 1 ms to 0.002 at 19 ms; autocorrelation increased from r = 0.75 at 1 ms to 0.97 at 19 ms (Fig. 6E-G, generated by Code 6 compute_predictability_metrics_by_delay and plot_predictability_metrics functions). Together these metrics characterize a continuous predictability gradient from exploratory/surprise-driven dynamics at short delays to confirmatory/expectation-driven dynamics at long delays, mapping naturally onto exploration-exploitation trade-offs in active inference.

3D surface visualizations of φ_{dyn}, FD, Higuchi FD, and DFA alpha as functions of delay and real time (Fig. 6A-D) were generated by Code 5 (create_smart_surface function), which read the CSV output from Code 4, converted time steps to real time by multiplying by delay, averaged across repetitions, and applied linear interpolation within each delay row to produce smooth surfaces. Phase-space attractor trajectories (Fig. 6H) were generated by Code 7 (build_attractors, plot_overlay_attractors functions), plotting φ_{dyn}(t+1) against φ_{dyn}(t) for each delay condition with individual runs shown in gray and the across-run average trajectory overlaid; optional Savitzky-Golay smoothing was applied to average trajectories only (window ≤ 21, polynomial order 2). All delays exhibited stable attractor dynamics with trajectories closely following the identity line (φ_{dyn}(t+1) ≈ φ_{dyn}(t)), confirming convergence toward equilibrium rather than chaotic or limit-cycle behavior. Short delays showed wider excursions from the identity line consistent with higher volatility; long delays remained tightly clustered, supporting a continuous predictability gradient rather than discrete regime transitions.

S2.3 Relationship between fractal measures and φ_{dyn}

φ_{dyn} and fractal measures (FD, HFD, DFA) capture complementary aspects of network organization and are visualized as parallel 3D surfaces in Fig. 6A-D. Fractal measures reflect stable spatial connectivity structure emerging from coincidence-based synaptic selection and are computed at each timestep by the analyze_fractal_properties method called within run_improved_delay_sweep (Code 3); φ_{dyn} reflects temporal alignment of branch-specific firing windows and is more sensitive to delay. The φ_{dyn} surface (Fig. 6A) shows greater variation at short delays reflecting larger transient fluctuations, while fractal surfaces (FD in Fig. 6B, Higuchi FD in Fig. 6C, DFA in Fig. 6D) rise from initial transients and stabilize into delay-dependent plateau values. Despite these differences, both φ_{dyn} and fractal metrics ultimately stabilize regardless of delay, with delay governing the dynamics of convergence rather than its endpoint, confirming that the RIFT network converges to an integrated fractal attractor across the full delay range tested.

Section S3: Multifractal Substrate and Generational Fractal Mapping - Part 3

S3.1 Information-theoretic equivalence of infinite spatial downscaling and generational fractal mapping

GFM is informationally equivalent to infinite spatial downscaling (ISD). In fractal geometry, the spatial scaling factor r describes how much smaller each embedded pattern becomes at each level. Reinterpreting r as an information-preservation rate, for example, r = 0.6 meaning 60% of information is retained at each smaller scale, gives the total information contained across infinite spatial embedding:

Eq. S6: $I_{\text{total}}^{\text{IDS}} = \frac{I(P_0)}{1-r}$ [IDS total information]

where P_0 = original parent fractal pattern at scale level 0; r = spatial information retention rate per scale level.

Similarly, GFM uses a temporal decay factor q representing how much information transfers from one generation to the next; if q = 0.6, each new generation preserves 60% of the previous generation's information, yielding cumulative information across generational succession:

Eq. S7: $I_{\text{total}}^{\text{GFM}} = \frac{I(S_0)}{1-q}$ [GFM total information]

where S_0 = initial seed pattern (generation 0); q = temporal information transfer factor (fraction of information preserved generation-to-generation).

When both systems preserve information at the same rate (q = r):

Eq. S8: $I_{\text{total}}^{\text{IDS}} = I_{\text{total}}^{\text{GFM}}$ when $q = r$ [IDS-GFM equivalence]

This equivalence demonstrates that temporal succession achieves the same information-theoretic capacity as infinite spatial embedding. Physical substrates cannot implement ISD, since embedding indefinitely many self-similar copies within a fixed molecular structure has no physical realization at finite size. Temporal succession faces no equivalent obstacle: a biological substrate can continue generating new fractal generations indefinitely without requiring infinite spatial subdivision. GFM therefore provides the physically realizable alternative, preserving the information-theoretic capacity of the idealized infinite-downscaling limit without requiring it.

S3.2 Code 8: Fractal EPSP-to-multifractal mapping

The mapping of fractal EPSP trains onto the somatic multifractal substrate rests on a geometric correspondence between dendritic branching architecture and spatial encoding in the soma. The Sierpinski carpet was chosen as the mapping geometry because it is the canonical two-dimensional multifractal in which each spatial subdivision contains a scaled replica of the whole, directly instantiating the whole-in-part property required by RIFT. The grid size of 243×243 follows naturally: 243 = 3^5, the grid dimension produced by five iterations of the ternary subdivision rule, sufficient to resolve four branch levels with spatial hierarchies at distinct scales.

The dendritic architecture follows the fractal branching rule established in S1 (Eq. 1: $L_n = L_0 \times r^{(n-1)}$), with scaling factor r = 0.6 and four branch levels (i = 1-4). EPSP amplitudes at each branch level follow $A_0 \cdot r^{(i-1)}$ by direct application of this branching rule to signal attenuation, reflecting the cumulative signal travel distance from synapse to soma, which is itself governed by the same fractal branching structure: A_0 is the peak EPSP amplitude at the primary (level 1) branch, set to 0.15 in baseline simulations consistent with moderate subthreshold depolarization, and each successive branch level attenuates

the signal by factor r, reflecting distance-dependent attenuation in neocortical Layer 5 pyramidal neurons (Williams and Stuart 2002; A_1 = 0.15, A_2 = 0.09, A_3 = 0.054, A_4 = 0.032). This ensures that the same fractal scaling rule governing dendritic tree geometry also governs the amplitude hierarchy of EPSPs arriving at the soma.

Peak amplitudes from each branch level are hierarchically assigned to nested spatial scales using a Sierpinski-like subdivision rule: the 243×243 grid is divided by successive factors of 3 (step sizes 81, 27, 9, 3), with the amplitude of each level placed at the center cell of each 3×3 block at that scale. Level 1 amplitudes map to the coarsest spatial scale (largest subdivisions), with successive levels assigned to progressively finer scales, so that proximal branch signals dominate the large-scale spatial structure and distal branch signals modulate fine-scale detail. Amplitudes across all branch levels are then additively integrated to generate the final multifractal intensity distribution, in which the spatial pattern preserves the full hierarchical amplitude structure of the dendritic EPSP train (Fig. 7A-D). The complete pipeline is implemented in Code 8 as a standalone instructional visualization using direct fractal amplitude scaling as a conceptual simplification, with A_0 = 1000.0 in arbitrary display units for the Code 8 visualization itself while preserving the same r = 0.6 scaling ratios used in the biological amplitude values above; the biological implementation with Gaussian kernel mapping is introduced in Part 4 (Code 10).

S3.3 Code 9: Generational Fractal Mapping (GFM) cycle

Generational Fractal Mapping implements the dynamic cycle by which the somatic multifractal sustains and updates conscious content across successive moments. Rather than mapping EPSPs onto a blank substrate at each moment, GFM integrates new EPSP input with a compressed representation of the preceding multifractal state, the peripheral seed, so that each conscious moment inherits structural information from its predecessor. The full GFM cycle is implemented in Code 9 as a standalone simulation (GrowingMultifractalGenerator class) running 40 evolution steps, with key parameters set via DendriticParameters: fractal_size = 243, firing_threshold = 180.0, growth_rate = 0.2, seed inheritance weight = 0.3 (30%), tau_rise = 0.3 ms, tau_decay = 4.0 ms, total_synapses = 500. At each step, the GFM cycle first checks whether collapse has occurred by computing center-region intensity (mean of the central third of the 243×243 grid, get_center_intensity).

Two branches follow: If center intensity has not reached the firing threshold (180.0), the fractal grows: new EPSP signals are generated by EPSPGenerator with amplitudes weighted by synapse count and activation level following $A_0 \cdot r$^(i-1). The active dendritic boundary expands by one branch level per generation (_get_activation method), modeling progressive proximal-to-distal recruitment of dendritic inputs across successive conscious moments. The new EPSP signal is then mapped onto the 243×243 grid by MultifractalGenerator.generate_fractal, incorporating the peripheral seed from the previous step at 30% weight (resized_seed × 0.3), the GFM inheritance step implementing differential encoding $M(t) = q \cdot Seed(t-1) + (1-q) \cdot \Delta EPSP(t)$ with q = 0.3. The resulting fractal then undergoes autonomous morphological expansion via grow_fractal_structure (expand_active_regions), with a step-dependent expansion radius (1 + step/10) while conserving total pattern energy through normalization (Fig. 8B, C).

If center intensity has reached the firing threshold, collapse is triggered: an action potential is generated (check_action_potential; Fig. 8C), and the highest-intensity corner quadrant (1/3 × 1/3 of the grid, selected from four corners by total intensity sum via np.argmax([np.sum(q) for q in quadrants]), extract_peripheral_seed) is extracted as the compressed peripheral seed for the next generation (Fig. 8A). A new initial multifractal state is then generated from fresh EPSP input (generate_new_initial_state), with the

extracted seed carried forward into the next non-collapse cycle. Regular collapse events occur approximately every 3-4 steps.

Whole-in-part metrics quantify holographic information preservation at each step (measure_whole_in_part; visualize_parent_daughter_relationship, Fig. 8B, D). Four metrics are computed: structural similarity (Structural Similarity Index (SSIM) between upscaled daughter seed and parent multifractal, normalized to [0,1]); reconstruction accuracy (SSIM after tiling the upscaled daughter into all four corners to reconstruct parent structure, attempt_reconstruction); information density ratio (sum of daughter intensity per unit area divided by sum of parent intensity per unit area); and pattern preservation (Intersection over Union of active regions thresholded at 0.3). All four metrics oscillate systematically with each collapse cycle (Fig. 8D); averaged values across all cycles confirm robust and consistent performance (Fig. 8F).

The differential encoding in GFM structurally resembles Bayesian updating: the seed carries compressed historical context (q fraction of prior information) acting as the prior, while new EPSPs contribute delta information acting as the likelihood update. The weighted integration $M(t) = q \cdot Seed(t-1) + (1-q) \cdot \Delta EPSP(t)$ achieves this through geometric pattern combination rather than probability calculus. The compression advantage is substantial: encoding complete cortical states requires mapping 10^6-10^7 active synapses onto ~10^4 molecular sites (100-1000:1 compression), while encoding deltas requires mapping only 10^4-10^5 changed events onto ~10^3 available sites (10-100:1 compression per cycle), achieved through fractal encoding of hierarchical EPSP amplitude distributions.

S3.4 φ*(multifractal) calculation (Code 9, compute_irreducibility_fixed)

After each EPSP-driven update, the multifractal pattern is partitioned into four equal spatial quadrants (top-left, top-right, bottom-left, bottom-right, each of size grid/2 × grid/2). The whole-pattern intensity distribution is computed as a 20-bin histogram over the range (0,1) of the fractal normalized to its own maximum; Shannon entropy is derived from this distribution as a complementary complexity metric. Each quadrant is independently normalized to its own maximum before computing its 20-bin histogram over the same (0,1) range; the four quadrant distributions are then averaged into a single combined quadrant distribution. KL divergence between the whole-pattern distribution and the combined quadrant distribution is computed as KL divergence = $\Sigma_i\, p(i) \times \log_2((p(i) + \varepsilon) / (q(i) + \varepsilon))$, where p(i) is the whole-pattern probability and q(i) is the combined quadrant probability at bin i, with $\varepsilon = 10^{-10}$ for numerical stability; bins where p(i) = 0 are skipped (standard KL convention, 0 × log 0 = 0). Wasserstein distance (Earth Mover's Distance) is computed separately between the whole-pattern distribution and each of the four quadrant distributions, using the bin centers derived from the whole-pattern histogram as the shared ground metric, then averaged across the four quadrants. The combined integration measure is integration = max(0, KL divergence) + 0.1 × mean Wasserstein distance, and φ*(multifractal) is then scaled by pattern complexity: φ*(multifractal) = integration × log(1 + complexity/1000), where complexity = sum of all intensity values in the unnormalized multifractal (np.sum(current_fractal)). This formulation ensures non-negativity and sensitivity to overall pattern richness: at low complexity the scaling term suppresses φ*(multifractal) regardless of KL divergence, while at high complexity the log term amplifies it proportionally. φ*(multifractal) peaks at GFM collapse events and remains sustained across generational cycling (Fig. 8E, F).

Eq. S9*: $\varphi^*(\text{multifractal}) = \text{integration} \times \log 1 + \text{complexity}/1000$ [irreducible spatial information] *the operational form of Eq. 9 actually computed by Code 9

where integration = max(0, KL divergence) + 0.1 × mean Wasserstein distance between whole-pattern and quadrant distributions; complexity = sum of all pattern intensity

values; KL divergence and Wasserstein distance computed between the 20-bin normalized intensity histogram of the whole multifractal and the mean histogram of four spatial quadrants treated independently (compute_irreducibility_fixed function, Code 9).

S3.5 Mathematical formalization of fractal logic and self-reference in GFM (Strange loops)

GFM also implements a fractal logic gate for resolving logical paradoxes such as the Liar's paradox, enabling contradictory states to coexist through fractal embedding rather than forcing premature binary resolution (Bieberich 2001). This section provides a formal proof of principle for this mechanism, demonstrating that fractal state composition is sufficient to maintain contradictory coexistence and non-halting deliberation as abstract computational properties. The biological network implementation of this principle is described in the main text (Parts 3-4), where bandwidth constraints yield the differential encoding form M(t) = q·Seed(t-1) + (1-q)·ΔEPSP(t). The abstract operator λ corresponds to q, implemented as the adaptive weight w in Code 11, spanning CR ≈ 0.5 (w ≈ 0.5, balanced deliberation) to CR → 1 (w → 1, seed-dominated consolidation near firing threshold); M_current(t) corresponds to the delta EPSP input. The distinctions reflect implementation constraints rather than a difference in underlying logic. GFM implements fractal computational logic through state composition across temporal generations:

$$M(\mathrm{t}) = \mathrm{M\_current(t)} \oplus \sigma(\mathrm{M\_seed(t-1)})$$

where: M(t) = conscious state at time t (a probability distribution over the fractal endospace); M_current(t) = current sensory/bottom-up input; M_seed(t-1) = previous state serving as seed; ⊕ = fractal composition operator (blends current + refined seed); σ = seed compression operator.

Concrete form:

$$M(\mathrm{t}) = (1-\lambda) \cdot \mathrm{M\_current(t)} + \lambda \cdot \mathcal{T}(\sigma(\mathrm{M\_seed(t-1)}))$$

where $\lambda \in (0,1)$ is the memory blend weight and $\mathcal{T}$ is the fractal refinement operator.

Fractal Gate

Classical logic requires S ∈ {A, ¬A}. The fractal gate enables S ∈ F(A, ¬A) through subspace embedding with non-separability: $S_i \cap S_j \neq \emptyset$ for all i ≠ j. This fractal logic gate was introduced in Bieberich (2001), where it was originally framed around a quantum-computational realization; the underlying logic, developed here in its classical form, requires no quantum mechanism. Each part of the fractal contains a rescaled copy of the whole, so any localized region of the fractal endospace simultaneously contains subregions supporting A (True) and subregions supporting ¬A (False); both truth states coexist within any single part.

Contradiction Ratio (CR)

$$CR = \frac{|M_1|}{|M_1| + |M_2|}$$

where M_1 = probability mass supporting proposition A; M_2 = probability mass supporting ¬A. CR determines computational mode: CR ≈ 0.5 → balanced contradiction → conscious deliberation; CR → 0 or 1 → asymmetric → automated processing. Paradox awareness score: $P = 4 \times M_1 \times M_2$ (with $|M_1| + |M_2| = 1$, ensuring $P \in [0,1]$). Maximum P = 1 when $M_1 = M_2 = 0.5$, corresponding to Hofstadter's strange loop: the system achieves

maximal self-reference by holding contradictory states in perfect balance, sustaining conscious deliberation rather than resolving the contradiction.

Non-Halting Property

$$\lim_{t\to\infty} CR(\mathrm{t}) \neq 0$$

The system never fully collapses because each time step re-injects both alternatives through seed refinement. Since $S_i \cap S_j \neq \emptyset$ by construction, no finite sequence of seed updates can fully evacuate probability mass from either subspace. The whole-in-part property guarantees that both A and ¬A retain minimum probability mass in every part of the fractal, making complete resolution structurally impossible, and conscious deliberation an ongoing, self-sustaining process.

Section S4: Biological Multifractal Implementation - Part 4

S4.1 Gaussian kernel EPSP-to-spatial-field mapping (Code 10; implemented in Code 11 as generate_epsp_field, _get_level_kernel)

Sierpinski-carpet subdivision in Part 3 (S3.2) was a representational convenience for the theoretical model, a simple way to assign hierarchical EPSP amplitudes to nested spatial scales on an idealized lattice, not a physical claim about how depolarization propagates. Real membrane depolarization does not subdivide a grid by discrete factors of three; it spreads continuously from each synaptic input source, attenuating with distance (Williams and Stuart 2002). A biologically grounded model therefore needs a spatial transformation with continuous falloff rather than discrete subdivision, which is what a Gaussian kernel provides. Part 4 departs from geometry in Part 3 at exactly this point: the transition from theoretical demonstration (idealized lattice, arbitrary subdivision) to biological implementation (physically motivated diffusion of depolarization).

Branch-level kernels $K_l(x,y)$ (Eq. 10) are constructed by _get_level_kernel, with proximal branch level 1 centered at the lattice center and successive levels offset to increasing radial distances. generate_epsp_field sums each level's kernel, weighted by its instantaneous EPSP amplitude, into the composite spatial field E(x,y,t) used by the channel update step (S4.2). The 100×100 Ising lattice (scalable to 243×243) used approximately 400 ion channels (4% density) with 3-pixel influence radius and 10% mobility.

Eq. S10: $E(\mathrm{x,y,t}) = \sum_l A_l(\mathrm{t}) \cdot K_l(\mathrm{x,y})$ [Gaussian kernel spatial mapping]

where $A_l(t)$ = EPSP amplitude from dendritic branch level l at time t; $K_l(x,y)$ = Gaussian kernel mapping this temporal signal to a spatial region on the somatic membrane, $K_l(x,y) = \exp(-\mathrm{dist}(x,y)^2/(2\times \mathrm{radius}_l^2))$, with dist computed to the level-specific center coordinate and $\mathrm{radius}_l = \mathrm{size}/4 - (l-1)\times 5$ (_get_level_kernel, Code 11).

S4.2 Ion channel opening probability (Eq. 11; implemented in Code 11 as IonChannel.update)

Channel opening probability is computed by IonChannel.update at each simulation step (Eq. 11). EPSP temporal signals used τ_rise = 0.3 ms and τ_decay = 4.0 ms.

Eq. S11: $P_{\mathrm{open}} = S(E + \lambda_{\mathrm{lipid}} + C_{\mathrm{coop}})$ [channel opening probability]

where S = sigmoid activation function with 0.5 baseline offset on the local EPSP field value E; λ_lipid = 0.25×lipid_state represents the local lipid bias term; C_coop = cooperative term summed over nearby open channels weighted by distance within the influence radius (IonChannel.update, Code 11).

S4.3 Ising lipid dynamics and the continuous-versus-blocked EPSP comparison (Codes 11-12)

Lipid dynamics evolved via Metropolis algorithm. At each Monte Carlo step, approximately 500 sites (5% of lattice) were randomly selected. Energy change (Eq. 19, computed within update_lipids, Code 11) determined acceptance: ΔE < 0 accepted unconditionally; otherwise accepted with probability exp(−ΔE×0.5).

Eq. S19: $\Delta E = 2 \times state \times \sum$ neighbors [Ising energy change]

where state = current binary lipid state (+1/−1) of the selected site; neighbors = the four lattice-adjacent lipid states summed with sign, so that ties with like-state neighbors lower energy and flipping against the local consensus raises it, the standard nearest-neighbor Ising interaction term. Action potentials were generated when center region activity exceeded threshold (30-40 units). Refractory period: refractory_steps = int(consciousness_level × 25), with consciousness_level = 0.5 in baseline simulations (~12 ms). The two integration modes were implemented via block_epsp_during_refractory (False = continuous, True = blocked). The _redistribute_channels function resets channel positions at each action potential, while the lipid composition matrix undergoes continuous Ising updates via update_lipids and is never reset, implementing molecular memory through persistent lipid configurations.

The logic of the continuous-versus-blocked comparison rests on this asymmetry. In continuous mode, channel states are repeatedly redriven by fresh EPSP input throughout the refractory period, so any organization observed in the substrate is confounded with ongoing synaptic drive and cannot be attributed to the lipid lattice alone. In blocked mode, EPSP input is withheld for the remainder of the refractory period after the initial programming step, removing the EPSP-driven term from channel opening probability (Eq. 11); channels remain active, but their opening is now governed only by local lipid bias and cooperative interactions with neighboring channels, both of which are in turn shaped by the ongoing Ising dynamics of the lipid lattice (update_lipids biases lipid states toward the preference of nearby open channels, and channel opening is biased toward local lipid state). Channels and lipids therefore continue to co-evolve in blocked mode rather than one substrate freezing while the other moves; what changes is that channel state is no longer set by an external EPSP signal but becomes a downstream consequence of lipid organization. This is why the blocked condition is diagnostic rather than merely a parameter variation: any further pattern development in this mode reflects the lipid lattice's autonomous, self-organizing dynamics propagating through to channel state, rather than the reverse, directly testing whether RIFT's claim of autopoietic membrane dynamics has a physical basis in the model. The component-contribution analysis underlying Fig. 9J is computed by analyze_component_contributions (Code 11).

S4.4 φ*(lipid) calculation (Code 13)

An initial φ*(lipid) implementation following S3.4's KL-divergence, quadrant-partition method was found to be insensitive to spatial reorganization of the lipid substrate: because the lipid composition lattice takes only two values (+1/−1), every spatial quadrant spans the identical full value range, so per-quadrant normalization collapses every quadrant's histogram onto the same two bins as the whole-pattern histogram regardless of actual domain organization, leaving KL divergence pinned at essentially zero for both Normal and Blocked conditions. This motivated a substrate-specific replacement, described below.

φ*(lipid) extended the integration-versus-partition principle of φ*(multifractal) (Eq. 9*, S3.4) to the molecular level. φ* is computed by calculate_phi_lipid_multiscale: integration is the mean of three non-negative terms, each comparing the full-resolution

lipid pattern to a 4×4-tile downsampled (coarser-grained) version of itself, the difference in low-frequency spectral power fraction (how much of the structure of a pattern is large-scale rather than fine-grained, measured via 2D Fourier transform), the difference in largest-connected-component fraction (how much of the pattern forms one continuous domain rather than many small fragments), and the difference in coarse-versus-fine-grained spatial mutual information (how predictable fine-scale structure is from coarse-scale structure), scaled by a saturating function of domain boundary density. In plain terms, this asks: does the lipid pattern have large-scale, connected, internally predictable structure that is lost when the pattern is coarsened, or is its apparent structure already present at the coarse scale (and therefore not genuinely whole-pattern information)? Lipid domain fractal dimension (FD, Fig. 9K) is computed separately by calculate_fractal_dimension using standard box-counting on the gradient-binarized (domain-boundary) lipid pattern.

Eq. S20: $\varphi^*(\text{lipid}) = \text{mean}(\text{lowk\_diff}, \text{lcc\_diff}, \text{mi\_diff}) \times \tanh \text{perimeter}/800$ [lipid integration, calculate_phi_lipid_multiscale]

where lowk_diff = difference in low-frequency spectral power fraction between full-resolution and tile-downsampled lipid pattern (loss of large-scale structure upon coarsening); lcc_diff = difference in largest-connected-component fraction, normalized by total active lipid sites (loss of domain connectedness upon coarsening); mi_diff = difference in mutual information between coarse- and fine-grained resamplings of the binarized domain pattern, computed at full resolution versus averaged across spatial tiles (loss of cross-scale predictability upon coarsening); perimeter = domain boundary length from the gradient magnitude of the lipid pattern (Code 13).

φ*(lipid) retains the same underlying logic as φ*(multifractal) in S3.4, quantifying information present in the whole pattern beyond what independent partitions or scales can capture. This analysis is restricted to the lipid substrate because the component-contribution analysis (S4.3, Fig. 9J) already establishes, independently of any φ* calculation, that lipid domains rather than ion channels drive multifractal evolution in this model; an information-integration measure for ion channel activity was not pursued further once this was established. Code 13 (RIFT_Code13_Lipid_FD_Phi_Unified.py) consolidates what were previously two separately-maintained lipid φ* implementations (one for the two-model Normal-versus-Blocked comparison, one for the three-model comparison including Temperature) into a single function used for both, correcting a coarse-graining error in the mutual information term (integer truncation of continuous block-averaged density values, which discarded intermediate density gradations) that was present in the three-model implementation.

FD and φ* are computed at every simulation step (n = 100 steps per condition, one continuous run each for normal and blocked mode), so reported means, standard deviations, and t-test comparisons summarize the within-run temporal trajectory rather than independent replicate simulations; the very small p-values reflect the large effect size and step count rather than independent-sample power, and should be read alongside the trajectories in Fig. 9K-L rather than in isolation.

Section S5: Holographic Endospace and Autopoietic Control - Part 5

S5.1 Stage 1: IFS extraction from EPSPs (Code 14)

IFS transforms were extracted from EPSP amplitude hierarchies using the EPSPToIFSDerivation.derive_ifs_from_epsp function in Code 14:

Eq. S12: $\{\text{EPSP}(t)\} \rightarrow \{\text{IFS\_transforms}(a, b, c, d, e, f)\} \rightarrow T(x, y)$

Each EPSP pattern yielded six affine geometric transforms $T_i(x,y) = (a_i \cdot x + b_i \cdot y + e_i, c_i \cdot x + d_i \cdot y + f_i)$ encoding spatiotemporal relationships: scaling factors (a, d) capture magnitude, rotation components (b, c) preserve hierarchical structure, and translation terms (e, f) encode dendritic integration patterns. Transform selection probabilities were weighted by determinant magnitudes.

S5.2 IFS temperature field generation (Codes 15-16)

The IFS-derived temperature field was generated in Codes 15-16:

Eq. S13: $Temp(\mathrm{x}, \mathrm{y}) = Temp_{\mathrm{base}} + \sum_i \mathrm{IFS\_influence}(T_i, \mathrm{x}, \mathrm{y})$

where Temp_base = baseline temperature parameter (0.5) and each IFS transform T_i contributes regional modifications based on geometric patterns derived from EPSP hierarchies.

The chaos game algorithm iteratively applied EPSP-derived IFS transforms, accumulating point visits: high-visit regions became warm (Temp → 1.0), sparse regions remained cool (Temp → 0.0). The IFS-derived temperature field Temp(x,y) drives lipid domain reorganization during the refractory period through a two-step process implementing holographic pattern recording (Fig. 10B, Stage 2) implemented in Codes 15 and 16. First, the chaos game algorithm (Codes 15 and 16, functions create_ifs_temperature_field and apply_ifs_to_temperature_region) iteratively applies the EPSP-derived IFS transforms to generate the spatial temperature distribution across the membrane grid. The algorithm accumulates multiple point visits: where IFS transforms repeatedly direct points creates high-density regions (appearing as warm red/orange), while regions receiving fewer visits remain low-density (appearing as cool blue). Second, the Metropolis algorithm uses this pre-computed temperature field to guide lipid reorganization: at each step, a lipid molecule is selected and a flip is proposed (from promoting channel opening, state +1/red, to inhibiting, state -1/blue, or vice versa). See S5.3 for details comparing three models with or without temperature modulation.

S5.3 Stage 2: Temperature-modulated lipid dynamics (Code 16)

The Metropolis algorithm was applied with the IFS-derived temperature field governing local flip acceptance via apply_temperature_modulated_lipid_dynamics (Code 16):

Eq. S14: $P_{\mathrm{flip}} = \exp\left(\frac{-\Delta E}{Temp_{\mathrm{local}}(\mathrm{x,y})}\right)$ for $\Delta \mathrm{E} > 0$; $P_{\mathrm{flip}} = 1$ for $\Delta \mathrm{E} \leq 0$

where Temp_local(x,y) = temperature at that lipid's position and ΔE = energy change from neighboring lipid interactions.

Three models are compared: Normal (continuous EPSPs), Blocked (autonomous dynamics), and Temperature (EPSPs blocked but IFS temperature modulation active). FD and φ*(lipid) for all three models are computed by the same unified analysis used for the two-model Normal-versus-Blocked comparison (Code 13, S4.4), extended to a third model condition; lipid φ* and FD values are therefore directly comparable in absolute magnitude across Fig. 9K-L and Fig. 11F-G. The temperature-dependent shift in lipid domain organization is directly visible in the evolved lipid pattern itself (Fig. 11D).

S5.4 Stage 3: Differential IFS extraction (Codes 19-20)

IFS transforms were extracted from final lipid domain patterns of both Temperature and Blocked models using extract_ifs_transforms_to_csv (Code 19), reverse-engineering affine parameters (a, b, c, d, e, f) from observed spatial organization. Differential

transforms computed via calculate_and_save_differential_transforms (Code 20) and saved as differential_transforms.csv:

Eq. S15: $H_{\text{RIFT}} = T_{\text{temperature}} - T_{\text{blocked}}$

S5.5 Stage 4: Self-attractor generation and endospace reconstruction (Codes 21-25)

The fractal Self-attractor was generated by applying the chaos game algorithm (generate_2d_attractor_data, Code 21) to H_RIFT, discarding first 500 iterations as transients and distributing 5,000 interference sources. Coherent phases follow Eq. 21:

Eq. S21: $\varphi_i = \left[\sum_j w_j \cdot \theta_j + 2\pi \cdot d_j/s_j\right] \bmod 2\pi$

where θ_j is the angle from source i to IFS center j, d_j is distance, s_j is IFS scaling factor, and $w_j = |\det_j| \cdot s_j$. Wave numbers derived from IFS scaling: f_i = 50/(1 + s_dominant) Hz (~40 Hz base frequency corresponding to somatic delay-determined network oscillation).

These wave numbers and distances are mathematical scaling factors operating in abstract transform parameter space, not physical membrane coordinates; the ~40 Hz figure reflects the GFM cycle rate at which geometric information is uploaded into the IFS transforms (Results, Parts 1-2), and should be read as a property of the encoding step rather than a real-time oscillation occurring during endospace projection itself. The 3D endospace field Ψ(x,y,z) is reconstructed on a 24×24×12 grid via project_to_endospace_coherent (Code 23):

Eq. S16 (T_E transformation): $\Psi(\mathrm{x,y,z}) = \sum_i \frac{A_i(H_{\text{RIFT}}) \cdot \exp[i \cdot (k_i \cdot r_i + \varphi_i)]}{1 + {r_i}^2}$

where A_i(H_RIFT) = amplitude from differential determinant values at source i; k_i = wave number derived from IFS scaling; r_i = Euclidean distance in transform space from source i to reconstruction coordinate; φ_i = coherent phase from geometric relationships to IFS transform centers (Eq. 21); the $1/(1+r_i^2)$ term implements distance-dependent decay.

Regional attractors from 8 spatial regions compared using Pearson correlation, SSIM, and Jensen-Shannon divergence. Three source positioning strategies are compared (Codes 24-25): chaos game (mean similarity = 0.96), regular grid (lower), and random placement (lowest).

Attractor dissimilarity (Fig. 13B, Code 24) was quantified via three metrics: Jensen-Shannon divergence (JSD; spatial distribution dissimilarity from pairwise 2D histograms, range [0,1], 0 = identical), Wasserstein distance (point-cloud earth mover's distance), and geometric feature dissimilarity (centroid, spread, eigenvalues, axis angle, eccentricity, convex hull area). Overall dissimilarity is the mean of JSD and geometric dissimilarity; Wasserstein distance is reported separately due to its different scale.

Endospace similarity (Fig. 13D-F, Code 25) was quantified via six metrics: Pearson correlation, SSIM, 3D shape similarity (sphericity, eigenvalues, Hausdorff distance), Jensen-Shannon divergence, mutual information, and fractal dimension similarity. The overall similarity score is a weighted composite (weights = 0.10, 0.15, 0.20, 0.25, 0.20, 0.10, respectively), with Jensen-Shannon divergence and mutual information weighted most heavily.

S5.6 Stage 5: Autopoietic feedback (Codes 26-27)

Mean endospace field intensity was extracted via Code 26 as spatial average of |Ψ(x,y,z)| per region; this computation is inline within extraction script of Code 26 rather than encapsulated in a dedicated function, for each region, psi_bar_mean = mean(field), read from Code 23's saved field arrays:

Eq. S17: $\bar{\Psi} = \langle|\Psi(x, y, z)|\rangle$

Autopoietic coupling modulates γ as a function of this mean field intensity, implemented in integrate_ifs_temperature_modulation (Code 16) when Ψ is supplied without an explicit coupling_gain override (effective_coupling_gain = 1.0 + alpha * psi_bar in the code):

Eq. S18 (T_A transformation): $\gamma(t) = \gamma_0(1 + \alpha \cdot \bar{\Psi}(t))$

where γ_0 = baseline coupling strength and α = feedback strength.

This is one of three branches in integrate_ifs_temperature_modulation, selected by which parameters are supplied: if Ψ is given without a manual coupling_gain, Ψ modulates γ directly as in Eq. 18 above; if both coupling_gain and Ψ are given, γ is instead held fixed and Ψ modulates IFS temperature-field strength (effective_ifs_strength = ifs_strength × (1 + $\alpha \times \Psi$)) rather than γ; if only coupling_gain is given, neither Ψ branch is used. The +6.5% channel-opening result reported below (γ = 3.0 vs. γ = 1.0) was obtained via the manual coupling_gain sweep (Code 27, run_coupling_final_state_test), a direct parameter variation rather than the Ψ-driven Eq. 18 pathway; the two are mechanistically related (both scale the same coupling parameter) but are distinct code paths and should not be read as the same experiment.

γ and α govern distinct aspects of the autopoietic feedback. γ (coupling strength) determines how strongly each ion channel weights its local lipid domain state when computing opening probability, implemented by scaling the lipid composition value passed to each channel's update function before the sigmoid calculation (Code 16; baseline lipid effect ±0.25, enhanced to ±0.25γ at coupling gain γ). α (feedback strength) determines the sensitivity of γ to the holographic field: in the $\Psi \rightarrow$ coupling mode, effective $\gamma = 1 + \alpha \cdot \Psi$, so a larger α amplifies the same Ψ into a stronger coupling modulation. The separation between γ and α means substrate sensitivity (γ) and feedback sensitivity (α) can be independently tuned, which is what coupling sweep in Fig. 14D tests: varying γ directly, without invoking Ψ or α, demonstrates the dose-response of channel behavior to lipid-channel coupling strength. Coupling strength varied across $\gamma \in$ {1.0-3.0}, nine values, n = 10 trials per condition (Code 27). One-way ANOVA confirmed significant effect; maximum coupling (γ = 3.0) achieved 95.5% channel opening fraction vs. 89.0% at γ = 1.0 (+6.5%). Temperature field spatial heterogeneity ($\sigma(T) \approx 0.15$) remained independent of coupling strength, confirming mechanistic separation.

Section S6: Sentyon Cloning Validation - Part 6

S6.1 Method 1: External position copying (Codes 30, 31)

Method 1 replicated the complete 3D spatial coordinates of all peripheral neurons connected to the source core neuron Nt into the target Nt+1 (Code 30, validated by Code 31). Source: somatic delay 6 ms, 435 connections. Method 1 achieved 100% connection utilization; target established 568 total connections (additional connections generated by target network's 15 ms somatic delay shifted normalized EPSP distribution, yielding 91.9% EPSP distribution similarity).

S6.2 Method 2: Synaptic position copying (Codes 29, 30)

Method 2 transferred connectivity at the dendritic level by copying branch-level synaptic assignments, then using coincidence detection to guide new connections to preoccupied positions (Code 29). Random peripheral neuron placement resulted in only 31.0% of reserved slots receiving appropriately timed coincident signals, yielding 135 total connections. However, these connections preserved branch-level proportionality, achieving 95.0% EPSP amplitude distribution similarity. Both methods converged on similar oscillation frequencies, confirming successful network entrainment.
